# Studies on the synthesis of peptides containing dehydrovaline and dehydroisoleucine based on copper-mediated enamide formation

**DOI:** 10.3762/bjoc.12.55

**Published:** 2016-03-22

**Authors:** Franziska Gille, Andreas Kirschning

**Affiliations:** 1 Institute of Organic Chemistry and Center of Biomolecular Drug Research (BMWZ), Leibniz University Hannover, Schneiderberg 1b, 30167 Hannover, Germany

**Keywords:** catalysis, dehydroamino acids, Hartwig–Buchwald reaction, myxovalargin, peptides

## Abstract

The preparation of peptide fragments containing dehydrovaline and dehydroisoleucine moieties present in the antibiotic myxovalargin is reported. Peptide formation is based on a copper-mediated C–N cross-coupling protocol between an acyl amide and a peptidic vinyl iodide. The presence of a neighboring arginine in the vinyl iodide posed a challenge with respect to the choice of the protecting group and the reaction conditions. It was found that ornithine – a suitable precursor – is better suited than arginine for achieving good yields for the C–N cross-coupling reaction. The optimized conditions were utilized for the synthesis of peptides **32**, **33**, **39** and **40** containing a neighboring ornithine as well as for the tripeptide **44** containing dehydroisoleucine with the correct stereochemistry.

## Introduction

**Scheme 1:**
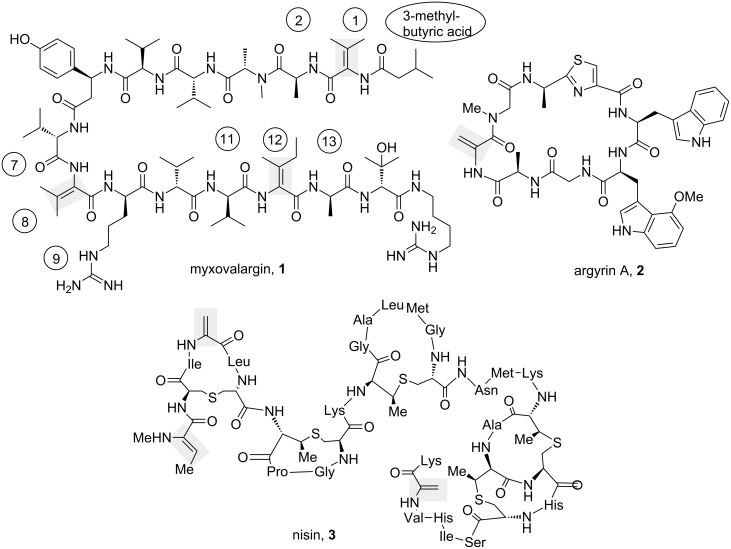
Selected examples of oligopeptides bearing dehydroamino acid moieties: myxovalargin (**1**), argyrin A (**2**) and nisin (**3**) (in myxovalargin dehydroamino acids and neighboring amino acids are numbered).

Dehydroamino acids [1] are rare amino acids that are constituents of many oligopeptides from microbial sources. Typical examples are myxovalargin (**1**), argyrin (**2**) and nisin (**3**, Scheme 1).

Dehydroamino acids and peptides are characterized by the presence of an olefinic double bond conjugated with the carboxyl or peptidic carbonyl group. Besides being α,β-unsaturated acids or amides, respectively, they can also be regarded as enamines. Due to the lack of reactivity of the amino group as well as the carboxylate, dehydroamino acids have hardly employed as building blocks in peptide synthesis. Therefore, the olefinic double bond is commonly introduced after the peptide backbone is assembled and typically this is achieved by elimination when a leaving group occupies the β-position [2]. Especially peptides containing dehydroalanine as found in argyrin (**2**) can be prepared from a precursor that contains a selenide substituent in the β-position [3]. Peptides that bear the doubly branched dehydroamino acids dehydrovaline or dehydroisoleucine, e.g., found in myxovalargin (**1**), are much more challenging to prepare due to steric hindrance in the β-position and the issue of regiocontrol during elimination [4–5], as β-elimination of a tertiary alcohol group often leads to the terminal instead of the conjugated alkene.

**Scheme 2:**
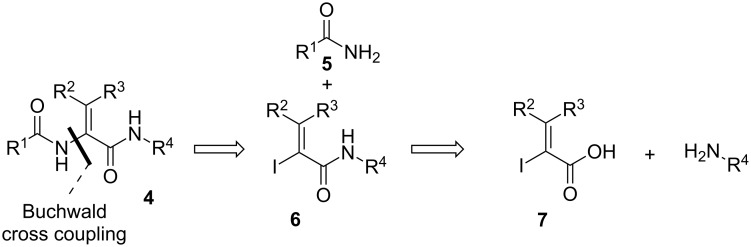
The Buchwald cross-coupling reaction in the preparation of peptides containing dehydroamino acids **4**.

Principally, enamides can also be prepared by the copper-mediated C–N coupling between a vinyl halide **6** and an amide **5** as reported by Ogawa and co-workers in 1991 [6]. Later, the group of Porco showed that copper(I) thiophencarboxylate is a suitable catalyst to promote this reaction in the presence of cesium carbonate as base and *N*-methylpyrrolidone (NMP) as solvent [7–8]. Buchwald et al. [9] simplified the conditions by demonstrating that copper(I) iodide, potassium carbonate and the ligand *N*,*N*-dimethylethylenediamine can be used instead. Finally, Ma and co-workers [10] further modified the conditions using copper(I) iodide, cesium carbonate and *N*,*N*-dimethylglycine in 1,4-dioxane. Recently, Inoue applied this cross-coupling protocol to the synthesis of peptides (Scheme 2) [11–12] as demonstrated in the total synthesis of Yaku'amide A [13].

As part of our study on the total synthesis of myxovalargin (**1**), a secondary metabolite from *Myxococcus fulvus* with antibacterial activity [14–15], we report on copper-mediated cross-coupling chemistry to create peptide fragments bearing dehydrovaline and dehydroisoleucine. We faced difficulties to use the reported conditions for the C–N cross coupling in the preparation of myxovalargin peptide fragments mainly because of the steric hindrance of β,β’-disubstituted dehydroamino acids created. Additionally, the neighboring amino acid, especially arginine or ornithine, a precursor for preparing arginine (amino acid number 9 in **1**), can be made responsible. Thus, this report covers our efforts to optimize the copper-mediated cross-coupling reaction in the preparation of dehydroamino acid containing peptide fragments present in myxovalargin (peptides containing amino acids 3-methylbutyric acid 1, 2, 7–9 and 11–13).

## Results and Discussion

**Scheme 3:**
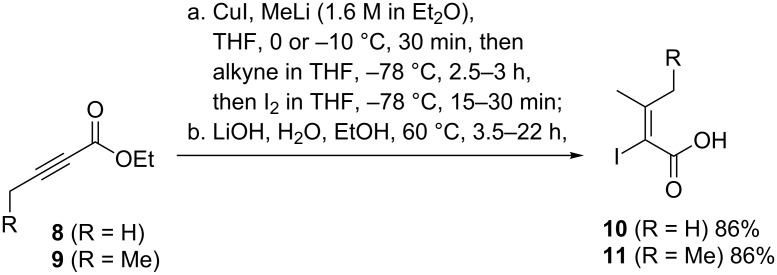
Syntheses of vinyl iodides **10** and **11**.

First vinyl iodide **10**, representing dehydrovaline, was prepared by an established sequence starting from alkynyl ester **8**. Under similar conditions vinyl iodide **11** which resembles dehydroisoleucine was obtained as a single diastereoisomer starting from alkynyl ester **9** (Scheme 3). The stereochemistry of the ethyl ester of **11** was determined NMR spectroscopically including nOe experiments (see Supporting Information File 1). Next, vinyl iodide **10** was subjected to amidation with L-alanine methylate (resembling position 2 in myxovalargin) using the reagent system PyAOP, HOAt, DIPEA to yield amide **12** (Table 1). Now, the stage was set to optimize the C–N coupling conditions using amide **13** as coupling partner, the 3-methylbutyric acid located at the terminus of myxovalargin (**1**).

**Table 1:** Formation of amide **12** and optimization of C–N cross coupling reaction.

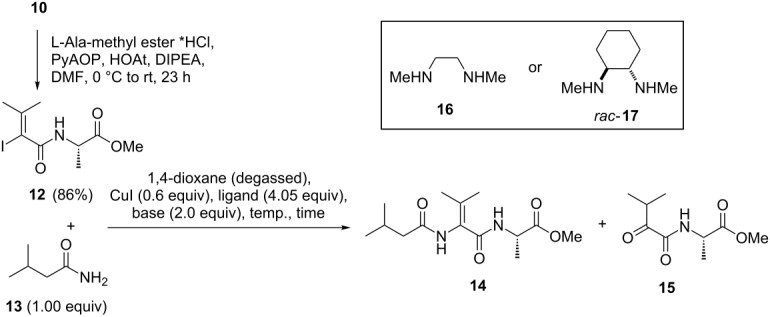					

entry	conditions	**12** (equiv)	**12** (% reisolated)	**14** (% yield)	**15** (% yield)

1	**16**, Cs_2_CO_3_, 16 h, 50 °C	1.5	–	31	–
2	**16**, Cs_2_CO_3_, 26 h, 50 °C	1.5	–	0	nd
3	**16**, Cs_2_CO_3_, 24 h, rt	1.5	60	~15	0
4^a^	TMEDA, Cs_2_CO_3_, 20 h, rt	1.5	no reaction		
5^a^	piperidine-2-carboxylate, K_2_CO_3_, 20 h, rt	1.5	no reaction		
6	**17**, K_2_CO_3_, 22 h, 50 °C	2.0	43	35	0
7	**17**, K_2_CO_3_, 20 h, 70 °C	2.0	57	35	0
8	**17**, Na_2_CO_3_, 20 h, 70 °C	2.0	66	25	0

^a^CuI (1.0 equiv) was used instead of 0.6 equiv of CuI.

Under the published conditions [13] (vinyl iodide (1.5 equiv), amide (1.0 equiv), CuI (0.6 equiv), Cs_2_CO_3_ (2.0 equiv), *N*,*N*-dimethylethylenediamine **16** (4.05 equiv), 1,4-dioxane, 90 °C) we did not encounter the formation of enamide **14** but instead only the hydrolysis product **15** was isolated which might have resulted from the presence of oxygen in the solvent. Therefore, we decreased the temperature and only degassed 1,4-dioxane was used in order to avoid oxidation and formation of copper(II) which can act as a Lewis acid. These changes provided peptide **14** (Table 1, entry 1) but this result turned out not to be reproducible. Instead, when the reaction time was extended, only the formation of the α-ketoamide **15** was encountered (Table 1, entry 2). Change of the solvent to THF or toluene as well as the use of palladium catalysts [16] or the use of additives such as HMPA mainly led to substantial decomposition of vinyl iodide **12**. At room temperature only small amounts of product were formed but the vinyl iodide was stable (Table 1, entry 3). The presence of the ligand was essential and the choice of other amines such as tetramethylethylendiamine (TMEDA) (Table 1, entry 4) or piperidine-2-carboxylate did not lead to product formation (Table 1, entry 5).

However, (*rac*)-*trans*-*N*,*N*-dimethyl-1,2-cyclohexanediamine (**17**) [9] in combination with cesium carbonate or potassium carbonate, the latter being superior to the former base, in 1,4-dioxane provided conditions that allowed us to prepare the coupling product **14** at room temperature. When raising the temperature to 50 °C the desired coupling product was isolated in 35% yield along with a substantial amount of starting vinyl iodide (Table 1, entry 6). When *N*,*N*-dimethylethylenediamine (**16**) was employed under these conditions instead, the coupling product formed only in traces. When potassium carbonate was exchanged by sodium carbonate the yield dropped to 25% under the optimized conditions (Table 1, entry 8).

**Scheme 4:**
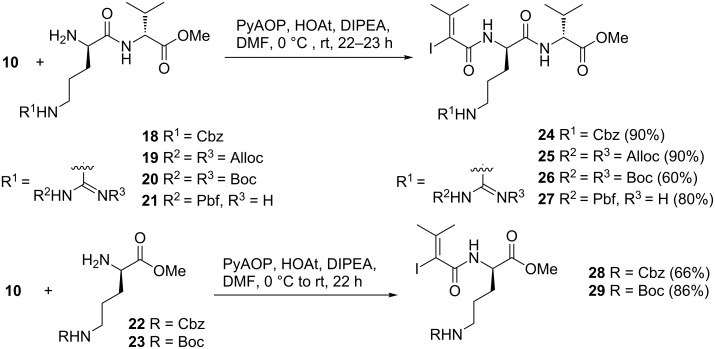
Preparation of vinyl iodides **24**–**29** (Cbz = benzyloxycarbonyl, Alloc = allyloxycarbonyl, Boc = *tert*-butyloxycarbonyl, Pbf = 2,2,4,6,7-pentamethyldihydrobenzofurane-5-sulfonyl).

Next, we tested these reaction conditions for the preparation of the other dehydrovaline bearing peptide fragment of myxovalargin **1** (amino acids 7–9). First, dipeptides **18**–**21** and methyl esters **22** and **23** were *N*-acylated with vinyl iodide **10** to yield peptidic vinyl iodides **24**–**29** in commonly good yields (Scheme 4). The coupling partners **18**–**23** (synthesis see Supporting Information File 1) all contain arginine or the precursor amino acid ornithine and they differ in the choice of the protecting group. These variations are important for gaining flexibility towards the end of the total synthesis when the guanidine group has either to be liberated by the removal of the protecting groups or used for the introduction to the corresponding ornithine residue.

These results demonstrate that the copper-mediated cross-coupling reaction depends on the functional groups present in the peptide containing the vinyl iodide moiety and on the chosen protecting groups. Vinyl iodides bearing an arginine moiety gave a significantly reduced yield of the desired cross-coupling product compared to the corresponding ornithine derivatives.

**Scheme 5:**
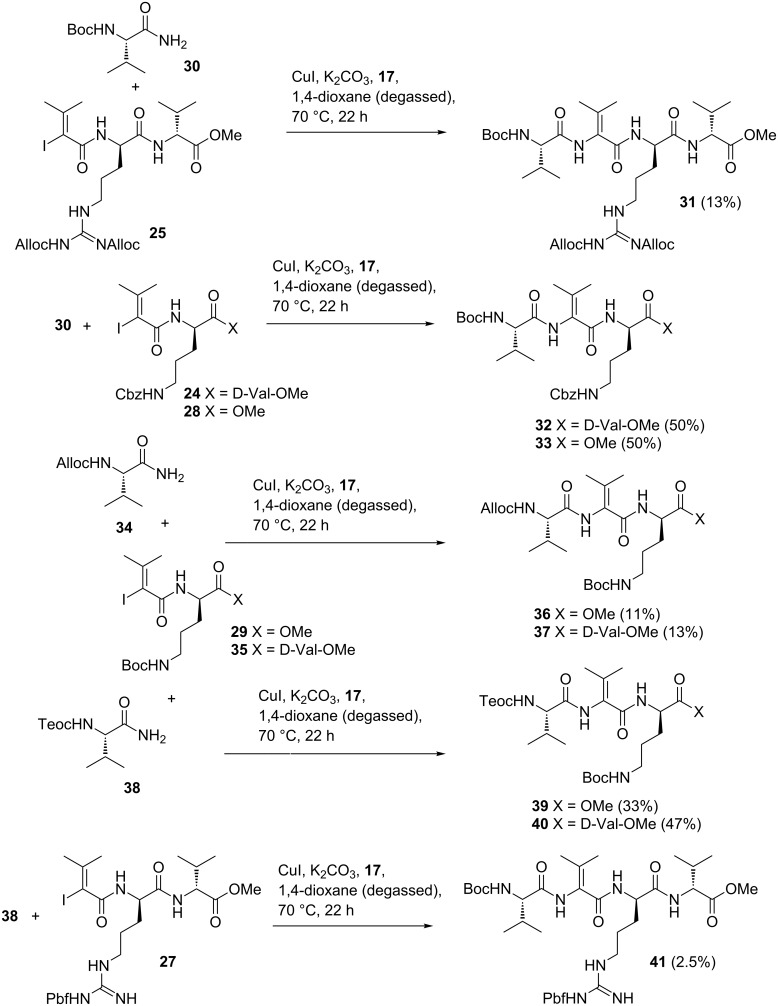
Copper-mediated C–N cross-coupling of dehydropeptides **31–33**, **36**, **37**, and **39**–**41**.

Furthermore, it turned out that the Alloc and Pbf protecting groups are not compatible for this enamide forming protocol. The best results were obtained by using the Boc-protected amide **30** and ornithine-containing vinyl iodides **24** and **28** (Scheme 5).

It needs to be noted that the presence of a Cbz protecting group in dehydrooligopeptides is problematic, because it cannot be cleaved without simultaneously reducing the acyl enamide. We found that the Teoc protecting group is better suited to be removed from oligoamides **39** and **40**, respectively, which were formed from amide **38** and vinyl iodides **29** and **35** (Scheme 5) [17]. Facile removal of the Teoc group was achieved within 24 h at room temperature in quantitative yield using a 1 M solution in TBAF in THF.

**Scheme 6:**
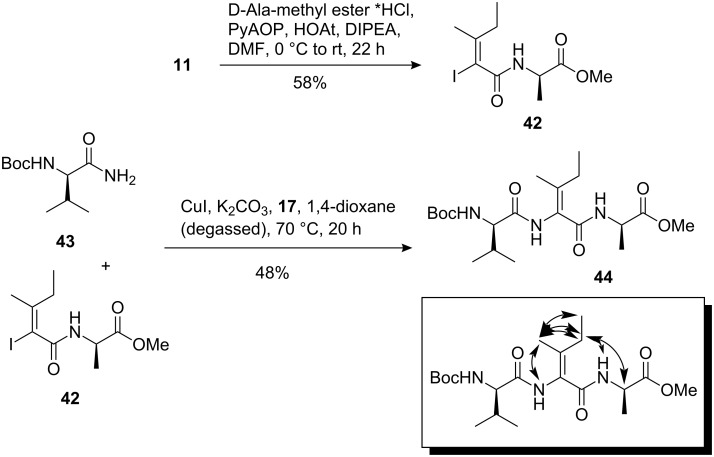
C–N coupling reaction between amide **43** and vinyl iodide **42**; formation of dehydroisoleucine containing peptide **44**. nOe assignments (tripeptide corresponds to amino acids 11–13 in myxovalargin (**1**)).

With these results in hand we next coupled amide **42** with Boc-protected valine-derived amide **43**. To our delight, the coupling proceeded in an improved yield of 48% without scrambling of the stereochemistry of the olefinic double bond (Scheme 6). We based the stereochemical assignment on NMR spectroscopy which included nOe experiments.

## Conclusion

In conclusion, we report on the synthesis of dehydrovaline and dehydroisoleucine-containing oligopeptides as found in the peptide antibiotic myxovalargin using a C–N cross-coupling approach. Substantial optimization of the reaction conditions and the choice of the protecting group became necessary when the dehydrovaline-containing oligopeptide based on ornithine was synthesized. For the copper-mediated Buchwald C–N coupling reaction, (*rac*)-*trans*-*N*,*N*-dimethyl-1,2-cyclohexandiamine was the ligand of choice in combination with potassium carbonate as base. In our case, the usage of Boc and Teoc protection groups in the cross-coupling reaction gave the best results. By using the optimized reaction conditions the dehydroisoleucine peptide was synthesized without scrambling of stereochemistry. In summary, we showed that the C–N coupling reaction is a powerful tool to straight forwardly build-up of sterically hindered dehydroamino acid-containing peptide fragments.

## Supporting Information

File 1 Syntheses and analytical descriptions of reagents and peptides and copies of ^1^H and ^13^C NMR spectra.
