# Supplementary material for: Studies on the synthesis of peptides containing dehydrovaline and dehydroisoleucine based on copper-mediated enamide formation
Source: Beilstein J Org Chem. 2016 Mar 22;12:564–70. doi: 10.3762/bjoc.12.55 (PMC4902033; doi:10.3762/bjoc.12.55)
Supplement: File 1 — Syntheses and analytical descriptions of reagents and peptides and copies of 1H and 13C NMR spectra. [file Beilstein_J_Org_Chem-12-564-s001.pdf]

Supporting Information

for

Studies on the synthesis of peptides containing dehydrovaline and dehydroisoleucine based on copper-mediated enamide formation

Franziska Gille and Andreas Kirschning*

Address: Institute of Organic Chemistry and Center of Biomolecular Drug Research (BMWZ), Leibniz University Hannover, Schneiderberg 1b, 30167 Hannover, Germany

Email: Andreas Kirschning - andreas.kirschning@oci.uni-hannover.de

* Corresponding author

Syntheses and analytical descriptions of reagents and peptides and copies of ^{1}H and ^{13}C NMR spectra

1. **General information**
2. **Description of synthetic procedures**
3. **References**

---

1. General information

All reactions were performed in oven-dried glassware under an atmosphere of nitrogen or argon, respectively. Tetrahydrofuran (THF) was distilled under nitrogen from sodium/benzophenone. Diethyl ether (Et_2O), methylene chloride (CH_2Cl_2) and *N*,*N*-dimethylformamide (DMF) were dried using a Braun Solvent Purification System (SPS). Commercially available reagents were used as supplied. Anhydrous magnesium sulfate and sodium sulfate were used as drying agents during work up. Unless otherwise noted, all

aqueous solutions used are saturated. If necessary, solvents were degassed prior to use by pump freeze-thaw technique.
Analytical thin-layer chromatography was performed using precoated silica gel ALUGRAM®Xtra SIL G/UV_{254} plates (Macherey-Nagel) and the spots were visualized with UV light at 254 nm or alternatively by staining with potassium permanganate or ninhydrin solutions.
Flash column chromatography was performed on Merck silica gel 60 (230–400 mesh). Eluents used for flash chromatography were distilled prior to use.
^{1}H NMR spectra were recorded at 400 MHz with a Bruker AVS-400 or Bruker DRX-400 as well as at 500 MHz with a Bruker DRX-500. Chemical shift values of ^{1}H NMR spectra are commonly reported as values in ppm relative to residual solvent signal as internal standard[S1]. The coupling constants are commonly reported in Hertz (Hz). Multiplicities are described using the following abbreviations: s = singlet, d = doublet, t = triplet, q = quartet, quin = quintet, sext = sextet, m = multiplet, b = broad.
^{13}C NMR spectra were recorded at 100 MHz with a Bruker AVS-400 or Bruker DRX-400 and at 125 MHz with a Bruker DRX-500. Chemical shift values of ^{13}C NMR spectra are commonly reported as values in ppm relative to residual solvent signal as internal standard[S1]. Multiplicities are reported using the following abbreviations: p = primary (methyl), s = secondary (methylene), t = tertiary (methine), q = quaternary (quaternary carbon). For full characterization ^{1}H ^{1}H correlation spectra (COSY) and ^{1}H ^{13}C correlation spectra (HMBC, HSQC) were recorded.
Mass spectra (EI) were obtained at 70 eV with a type VG Autospec spectrometer (Micromass), with a type LCT (ESI) (Micromass) or with a type Q-TOF (Micromass) spectrometer in combination with a Waters Aquity Ultraperformance LC system.

Preparative high performance liquid chromatography was performed by using a Merck Hitachi LaChrom system [pump L- 7150, interface D-7000, diode array detector L-7450 (λ = 220–400 nm, preferred monitoring at λ = 230 nm)] with the following columns: Trentec Reprosil-Pur 120 C18 ISIS AQ 5 µm, 250 × 8 mm, with guard column Trentec Reprosil-Pur 120 C18 AQ 5 µm, 40 × 8 mm. Alternatively, a Varian HPLC system [pump Prepstar Model 218, variable wavelength detector Prostar (λ = 248 nm)] with parallel mass detection (Mircomass typ ZMD ESI quad spectrometer) was used in combination with column: Trentec Reprosil-Pur 120 C18 AQ 5 µm, 250 mm × 25 mm, with guard column Trentec Reprosil-Pur 120 C18, AQ 10 µm, 30 mm × 20 mm. Membrane filtered, bidistilled water as well as commercial available HPLC quality solvents were used, which were degassed prior to use by using a ultrasonic bath.

Optical rotations [α] were measured on a Polarimeter 341 (Perkin Elmer) at a wavelength of 589 nm and are given in 10^{-1} deg cm^2 g^{-1}.
Melting points were measured using a SRS OptiMelt apparatus (Stanford research system).

2. Description of synthetic procedures

Table of content:

Synthesis of compound **10**
Synthesis of compound **11** through intermediate **S1**
Synthesis of compound **12**
Synthesis of compound **13**
Synthesis of compound **14**
Synthesis of compound **24** through intermediate **S2**
Synthesis of compound **25** through intermediates **S3**-**S5**
Synthesis of compound **20** through intermediates **S6-S9**
Synthesis of compound **26**
Synthesis of compound **21** through intermediate **S10**
Synthesis of compound **27**
Synthesis of compound **23** through intermediate **S11**
Synthesis of compound **29**
Synthesis of compound **35**
Synthesis of compound **28**
Synthesis of compound **30**
Synthesis of compound **31**
Synthesis of compound **32**
Synthesis of compound **33**
Synthesis of compound **34** through intermediate **S12**
Synthesis of compound **36**
Synthesis of compound **37**
Synthesis of compound **38** through intermediates **S13-S15**
Synthesis of compound **39**
Synthesis of compound **40**
Synthesis of compound **41**
Synthesis of compound **42**
Synthesis of compound **43**
Synthesis of compound **44**

Synthesis of 2-iodo-3-methylbut-2-enoic acid (10)

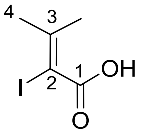

To a solution of CuI (2.46 g, 12.9 mmol, 1.50 equiv) in THF (42.5 mL) at −10 °C MeLi (1.6 M in Et_2O; 16.2 mL, 25.9 mmol, 3.00 equiv) was added. After 30 min, the reaction mixture was cooled to −78 °C and charged dropwise with ethyl 2-butynoate (**8**; 967 mg, 8.62 mmol, 1.00 equiv) in THF (5.00 mL). The reaction mixture was stirred for 2.5 h at −78 °C and subsequent a solution of iodine (6.57 g, 25.9 mmol, 3.00 equiv) in THF (5.00 mL) was added dropwise. The reaction mixture was stirred for additional 30 min at −78 °C and then terminated by addition of a saturated $Na_2S_2O_3$ solution. The layers were separated and the aqueous layer was extracted with Et_2O (4×). The combined organic layers were washed with a saturated NH_4Cl solution, followed by brine. The combined organic layers were dried over $MgSO_4$, filtered and concentrated under reduced pressure. The crude product was directly used in the next step without further purification.

The ethyl ester was dissolved in ethanol (2.00 mL) at rt. Then, LiOH (826 mg, 34.5 mmol, 4.00 equiv) in H_2O (15.0 mL) was added and the reaction mixture was heated to 60 °C. After 3.5 h, the reaction mixture was cooled to rt and EtOAc was added and the layers were separated. The aqueous layer was acidified with HCl (1.0 M) to pH ~ 1 and subsequently extracted with EtOAc (4×). The combined organic layers were washed with brine and then dried over $MgSO_4$, filtered and concentrated under reduced pressure. Acid **10** (1.68 g, 7.41 mmol, 86% over two steps) was obtained as a yellow solid. The analytical and physical data were in accordance with those reported in the literature[S2].

mp = 74 – 78 °C (mp = 79 – 80 °C ref.[S3]); ^{1}H-NMR (400 MHz, $CDCl_3$, $CHCl_3$ = 7.26 ppm): δ = 2.25 (3H, s, H-4), 2.17 (3H, s, H-4) ppm; ^{13}C-NMR (100 MHz, $CDCl_3$, $CDCl_3$ = 77.16 ppm): δ = 170.5 (q, C-1), 157.1 (q, C-3), 84.5 (q, C-2), 33.5 (p, C-4), 23.5 (p, C-4) ppm; HRMS (ESI): *m/z* calculated for $C_5H_6IO_2[M - H]^-$: 224.9413 found 224.9416.

Synthesis of compound 11 through intermediate S1

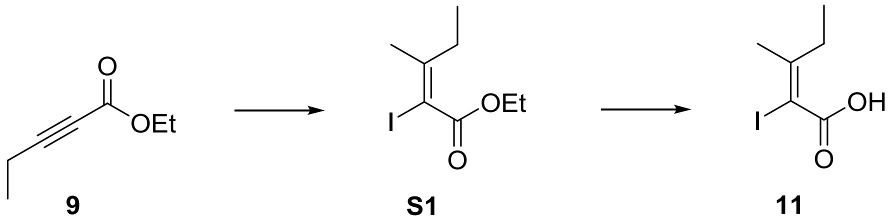

9 **S1** **11**

Synthesis of ethyl (*E*)-2-iodo-3-methylpent-2-enoate (S1)

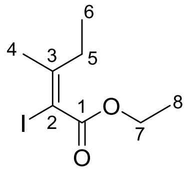

To a solution of CuI (2.86 g, 15.0 mmol, 1.50 equiv) in THF (42.5 mL) MeLi (1.6 M in Et_2O; 18.8 mL, 30.0 mmol, 3.00 equiv) was added at 0 °C. After 30 min, the reaction mixture was cooled to −78 °C and charged dropwise with ethyl pent-2-ynoate (**9**; 1.26 g, 10.0 mmol, 1.00 equiv) in THF (10 mL). The reaction mixture was stirred for 3 h at −78 °C and subsequently a solution of iodine (7.61 g, 30.0 mmol, 3.00 equiv) in THF (10 mL) was added dropwise. The reaction mixture was stirred for additional 15 min at −78 °C and then terminated by addition of a saturated $Na_2S_2O_3$ solution. The layers were separated and the aqueous layer was extracted with Et_2O (4×). The combined organic phases were washed with a saturated NH_4Cl solution, followed by brine. The combined organic phases were dried over $MgSO_4$, filtered and concentrated under reduced pressure. Product **S1** was directly used without further purification.

^{1}H-NMR (400 MHz, $CDCl_3$, $CHCl_3$ = 7.26 ppm): δ = 4.25 (2H, q, *J* = 7.2 Hz, H-7), 2.45 (2H, q, *J* = 7.5 Hz, H-5), 2.05 (3H, s, H-4), 1.32 (3H, t, *J* = 7.2 Hz, H-8), 1.08 (3H, t, *J* = 7.5 Hz, H-6) ppm; ^{13}C-NMR (100 MHz, $CDCl_3$, $CDCl_3$ = 77.16 ppm): δ = 165.9 (q, C-1), 155.6 (q, C-3), 84.8 (q, C-2), 62.0 (s, C-7), 30.1 (p, C-4), 28.8 (s, C-5), 14.2 (p, C-8), 13.0 (p, C-6) ppm; HRMS (ESI): *m/z* calculated for $C_8H_{14}IO_2$ $[M + H]^+$: 269.0039; found 269.0038.

nOe (400 MHz, $CDCl_3$):

proton	nOe contact
H-4	H-5, H-6
H-5	H-4, H-6
H-6	H-5
H-7	H-8
H-8	H-5, H-6, H-7

Synthesis of (*E*)-2-iodo-3-methylpent-2-enoic acid (11)

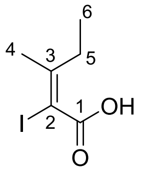

Ethyl ester **S1** (348 mg, 1.30 mmol, 1.00 equiv) was dissolved in a mixture of ethanol/H_2O (degassed; 1:7.5; 2.50 mL) at rt. Then, LiOH (125 mg, 5.21 mmol, 4.00 equiv) was added and the reaction mixture was heated to 60 °C. After 22.5 h, the reaction mixture was cooled to rt and EtOAc was added. The layers were separated. The aqueous layer was acidified with HCl (1.0 M) to pH ~ 1 and then extracted with EtOAc (4x). The combined organic layers were washed with brine and then dried over $MgSO_4$, filtered and concentrated under reduced pressure. Acid **11** (268 mg, 1.12 mmol, 86% over two steps) was obtained as a yellow solid which was directly used without further purification.

Synthesis of methyl (2-iodo-3-methylbut-2-enonyl)-L-alaninate (12)

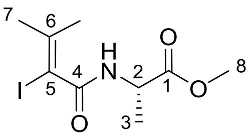

Acid **10** (600 mg, 2.65 mmol, 1.00 equiv) and L-alanine methylester hydrochloride (**11**; 482 mg, 3.45 mmol, 1.30 equiv) were dissolved in DMF (14.4 mL) at 0 °C. Then, HOAt (387 mg, 2.84 mmol, 1.07 equiv) and PyAOP (1.45 g, 2.79 mmol, 1.05 equiv) were added. Afterwards, DIPEA (1.57 g, 12.2 mmol, 4.60 equiv) was added dropwise. The reaction mixture was warmed up to rt. After 23 h, the reaction was stopped by adding saturated NH_4Cl solution. The layers were separated and the aqueous layer was extracted with EtOAc (7×). The combined organic layers were washed with brine, dried over Na_2SO_4, filtered and finally concentrated under reduced pressure. Purification by flash column chromatography on silica (petroleum ether/ethyl acetate 3:1) yielded the desired product **12** (710 mg, 2.28 mmol, 86%) as a colourless solid.

R_f = 0.64 (CH_2Cl_2/CH_3OH 98:2); mp= 74 – 76 °C; $[\alpha]_D^{20}$ = -10.8 (*c* 1.18, CH_2Cl_2); ^{1}H-NMR (400 MHz, $CDCl_3$, $CHCl_3$ = 7.26 ppm): δ = 6.26 (1H, brm, NH), 4.61 (1H, dq, *J* = 7.2 Hz, H-2), 3.77 (3H, s, H-8), 2.06 (3H, s, H-7), 2.01 (3H, s, H-7), 1.45 (3H, d, *J* = 7.2 Hz, H-3) ppm; ^{13}C-NMR (100 MHz, $CDCl_3$, $CDCl_3$ = 77.16 ppm): δ = 173.4 (q, C-1), 168.8 (q, C-4), 146.3 (q, C-6), 86.7 (q, C-5), 52.7 (p, C-8), 48.7 (t, C-2), 30.2 (p, C-7), 22.1 (p, C-7), 18.3 (p, C-3) ppm; HRMS (ESI): *m/z* calculated for $C_9H_{15}INO_3$ $[M + H]^+$: 312.0097 found 312.0096.

Synthesis of 3-methylbutanamide (13)

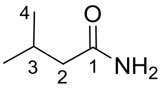

3-Methylbutanoic acid (6.00 g, 58.7 mmol, 1.00 equiv) and *N*-methylmorpholine (6.42 g, 63.4 mmol, 1.08 equiv) were dissolved in 1,2-dimethoxyethane (300 mL) at 0 °C. Then, isobutylchloroformiate (8.66 g, 63.4 mmol, 1.08 equiv) was added dropwise. The reaction mixture was stirred for 15 min at 0 °C. Afterwards, an aqueous ammonia solution (25%;

26.0 mL, 386 mmol, 6.57 equiv) was added dropwise. Now, the reaction mixture was stirred at 0 °C → rt. After 14 h, the reaction was terminated by adding HCl (1.0 M). The aqueous layer was extracted with EtOAc (4×). The combined organic layers were washed with HCl (0.1 M) and subsequently dried over $MgSO_4$, filtered and concentrated under reduced pressure. Amide **13** (4.14 g, 40.8 mmol, 70%) was isolated as a colourless solid.

mp = 123 – 126 °C; ^{1}H-NMR (400 MHz, DMSO-d_6, DMSO-d_5 = 2.50 ppm):δ = 7.22 (1H, s, NH), 6.70 (1H, s, NH), 2.00-1.88 (3H, m, H-2, H-3), 0.96-0.89 (6H, m, H-4) ppm; ^{13}C-NMR (100 MHz, DMSO-d_6, DMSO-d_6 = 39.52 ppm): δ = 173.8 (q, C-1), 44.4 (s, C-2), 25.3 (t, C-3), 22.3 (2C, p, C-4) ppm; HRMS (ESI): *m/z* calculated for $C_5H_{12}NONa$ $[M + H]^+$: 102.0919; found 102.0923.

Synthesis of methyl [3-methyl-2-(3-methylbutanamido)but-2-enoyl]-L-alaninate (14)

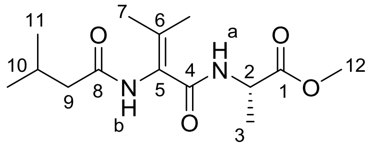

3-Methylbutanamide (**13**; 20.3 mg, 0.20 mmol, 1.00 equiv) and vinyl iodide **12** (125 mg, 0.40 mmol, 2.00 equiv) were dissolved in 1,4-dioxane (degassed; 0.25 mL) at rt. Then, K_2CO_3 (125 mg, 0.40 mmol, 2.00 equiv), CuI (23.0 mg, 0.12 mmol, 0.60 equiv) and *trans*-*N*,*N*-dimethyl-1,2-cyclohexanediamine (116 mg, 0.81 mmol, 4.05 equiv) were added. The reaction mixture was heated to 70 °C. After 20 h, the solvent was removed under reduced pressure. The resulting residue was diluted with a saturated NH_4Cl solution. Then, the aqueous layer was extracted with EtOAc (4×). The combined organic layers were washed with brine and subsequently dried over $MgSO_4$, filtered and concentrated under reduced pressure. Purification by flash chromatography on silica (100% CH_2Cl_2 → CH_2Cl_2/CH_3OH 99:1 → CH_2Cl_2/CH_3OH 98:2) gave the desired α,β-dehydropeptide **14** (20.0 mg, 70.3 µmol, 35%) as a colourless solid.

R_f = 0.37 (CH_2Cl_2/CH_3OH= 98:2); mp = 126 – 129 °C; $[\alpha]_D^{20}$ = -2.1 (*c* 0.81, CH_2Cl_2); ^{1}H-NMR (400 MHz, $CDCl_3$, $CHCl_3$ = 7.26 ppm): δ = 7.15 (1H, s, NH-b), 6.97 (1H, d, *J* = 7.2 Hz, NH-a), 4.58 (1H, dq, *J* = 7.2 Hz, H-2), 3.73 (3H, s, H-12), 2.15 (3H, m, H-9, H-10), 1.98 (3H, s, H-7), 1.73 (3H, s, H-7), 1.42 (3H, d, *J* = 7.2 Hz, H-3), 0.99-0.97 (6H, d, *J* = 6.1 Hz, H-11) ppm; ^{13}C-NMR (100 MHz, $CDCl_3$, $CDCl_3$ = 77.16 ppm): δ = 173.3 (q, C-1), 172.8 (q, C-8), 166.7 (q, C-4), 137.9 (q, C-5), 124.9 (q, C-6), 52.5 (p, C-12), 48.3 (t, C-2), 45.6 (s, C-9), 26.4 (t, C-10), 22.61 (p, C-11), 22.56 (p, C-11), 20.6 (p, C-7), 20.4 (p, C-7), 18.2 (p, C-3) ppm; HRMS (ESI): *m/z* calculated for $C_{14}H_{24}N_2O_4Na$ $[M + Na]^+$: 307.1634; found 307.1633.

Analytical data for the decomposition product 15:

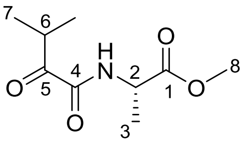

^{1}H-NMR (400 MHz, $CDCl_3$, $CHCl_3$ = 7.26 ppm): δ = 7.44 (1H, m, NH), 4.55 (1H, dq, J = 7.5 Hz, H-2), 3.76 (3H, s, H-8), 3.56 (1H, sept, J = 6.8 Hz, H-6), 1.45 (3H, d, J = 7.4 Hz, H-3), 1.12 (6H, d, J = 6.8 Hz, H-7) ppm; ^{13}C-NMR (100 MHz, $CDCl_3$, $CDCl_3$ = 77.16 ppm): δ = 201.7 (q, C-5), 172.5 (q, C-1), 159.4 (q, C-4), 52.7 (p, C-8), 48.1 (t, C-2), 34.2 (t, C-6), 18.2 (p, C-3 o. C-7), 17.82 (p, C-3 o. C-7), 17.80 (p, C-3 o. C-7) ppm; HRMS (ESI): m/z calculated for $C_9H_{15}NO_4Na$ $[M + Na]^+$: 224.0899; found 224.0902.

Synthesis of compound 24 through intermediate S2

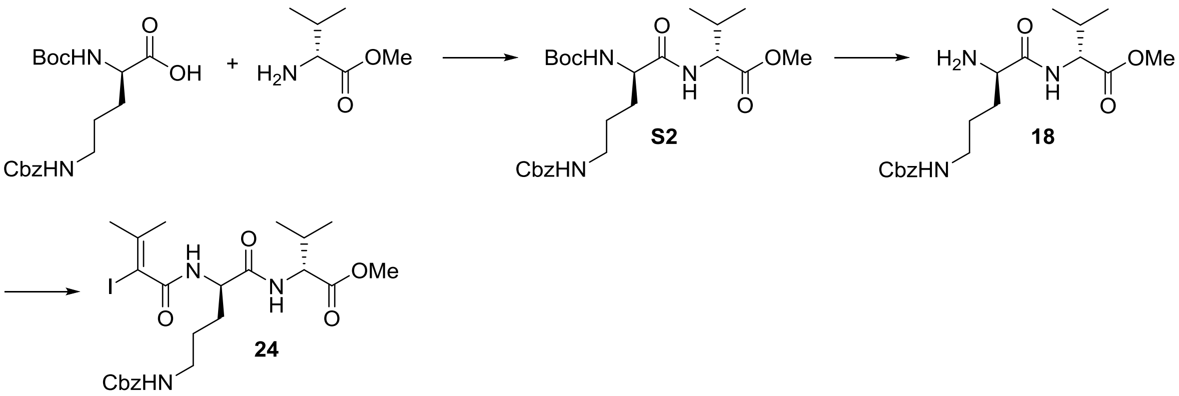

Synthesis of methyl [(*R*)-5-{[(benzyloxy)carbonyl]amino}-2-[(*tert*-butoxyvarbonyl) aminopentanoyl]-D-valinate (S2)

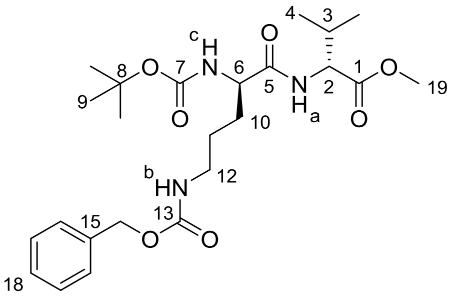

Boc-D-Orn(Cbz)-OH (200 mg, 0.55 mmol, 1.0 equiv) and D-valine methylester hydrochloride (101 mg, 0.60 mmol, 1.10 equiv) were dissolved in CH_2Cl_2 (64.0 mL) at rt. The reaction mixture was cooled to 0 °C and HOBt (111 mg, 0.82 mmol, 1.50 equiv) was added. Then, DIPEA (106 mg, 0.82 mmol, 1.50 equiv) was added dropwise followed by EDC·HCl (131 mg, 0.68 mmol, 1.25 equiv). The reaction mixture was stirred for 16 h at 0 °C → rt. The solvent

was removed under reduced pressure. Purification by flash chromatography on silica (CH_2Cl_2/CH_3OH = 99:1) gave dipeptide **S2** (260 mg, 0.54 mmol, 99%) as colourless oil.

R_f = 0.23 (CH_2Cl_2/CH_3OH = 99:1); $[\alpha]_D^{20}$ = -3.9 (*c* 1.30, CH_2Cl_2); ^{1}H-NMR (400 MHz, $CDCl_3$, $CHCl_3$ = 7.26 ppm): δ = 7.35 (5H, m, ArH), 6.88 (1H, brd, *J* = 7.9 Hz, NH-a), 5.17 (1H, brd, *J* = 7.2 Hz, NH-c), 5.11 (2H, m, H-14), 4.96 (1H, brt, *J* = 4.1 Hz, NH-b), 4.52 (1H, dd, *J* = 4.1, 7.9 Hz, H-2), 4.34 (1H, m, H-6), 3.71 (3H, s, H-19), 3.45 (1H, brs, H-12), 3.16 (1H, brs, H-12'), 2.19 (1H, sept, *J* = 7.9 Hz, H-3), 1.88 (1H, brs, H-10), 1.74 (1H, brs, H-10'), 1.59 (2H, m, H-11), 1.44 (9H, s, H-9), 0.94 (6H, d, *J* = 7.9 Hz, H-4) ppm; ^{13}C-NMR (100 MHz, $CDCl_3$, $CDCl_3$ = 77.16 ppm): δ = 172.4 (2C, q, C-1, C-5), 157.2 (q, C-13), 156.0 (q, C-7), 136.7 (q, C-15), 128.7 (2C, t, C-17), 128.3 (t, C-18), 128.3 (2C, t, C-16), 80.0 (q, C-8), 66.9 (s, C-14), 57.3 (t, C-2), 53.1 (t, C-6), 52.3 (p, C-19), 39.8 (s, C-12), 31.1 (t, C-3), 30.1 (s, C-10), 28.5 (3C, p, C-9), 26.4 (s, C-11), 19.2 (p, C-4), 17.8 (p, C-4) ppm; HRMS(ESI): *m/z* calculated for $C_{24}H_{37}N_3O_7Na$ $[M + Na]^+$: 502.2529; found 502.2524.

Synthesis of methyl [(*R*)-5{[(benzyloxy)carbonyl]amino}-2-(2-iodo-3-methylbut-2-enamido)pentanoyl]-D-valinate (24)

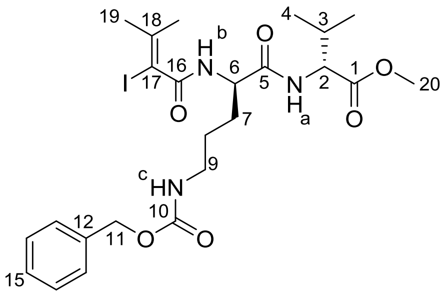

Boc-protected dipeptide **S2** (118 mg, 0.25 mmol, 1.00 equiv) was dissolved in CH_2Cl_2 (3.60 mL) at 0 °C. Afterwards, TFA (1.41 g, 12.3 mmol, 50.0 equiv) was added dropwise. After 2.5 h the reaction was terminated by addition of a saturated sodium bicarbonate solution. The layers were separated and the aqueous layer was extracted with EtOAc (4×). The combined organic layers were dried over $MgSO_4$, filtered and concentrated under reduced pressure. The title compound **18** (93.6 mg, 0.25 mmol, quantitative) was obtained as a colourless oil. The product was used without further purification.

Acid **10** (50.7 mg, 0.22 mmol, 1.00 equiv) and dipeptide **18** (93.6 mg, 0.25 mmol, 1.10 equiv) were dissolved in DMF (1.20 mL) at 0 °C. Then, HOAt (32.7 mg, 0.24 mmol, 1.07 equiv) and PyAOP (123 mg, 0.24 mmol, 1.05 equiv) were added. Afterwards, DIPEA (133 mg, 1.03 mmol, 4.60 equiv) was added dropwise. The reaction mixture was warmed up to rt. After 22 h, the reaction was terminated by addition of a saturated NH_4Cl solution. The layers were separated and the aqueous layer was extracted with EtOAc (7×). The combined organic

layers were washed with brine and subsequently dried over Na_2SO_4, filtered as well as concentrated under reduced pressure. Purification by flash column chromatography over silica (petroleum ether/ ethyl acetate = 3:1 → petroleum ether/ ethyl acetate = 1:1) gave the desired vinyl iodide **24** (119 mg, 0.20 mmol, 90%) as a colourless solid.

R_f = 0.70 (CH_2Cl_2/CH_3OH 98:2); mp = 143 – 146 °C; $[\alpha]_D^{20}$ = +4.2 (*c* = 1.73, CH_2Cl_2); ^{1}H-NMR (400 MHz, $CDCl_3$, $CHCl_3$ = 7.26 ppm): δ = 7.37-7.28 (5H, m, ArH), 7.05 (1H, d, *J* = 8.9 Hz, NH-a), 6.49 (1H, d, *J* = 8.2 Hz, NH-b), 5.12 (2H, t, *J* = 6.1 Hz, H-11), 5.03 (1H, m, NH-c), 4.80 (1H, m, H-6), 4.51 (1H, dd, *J* = 4.9, 8.8 Hz, H-2), 3.71 (3H, s, H-20), 3.57 (1H, m, H-9), 3.15 (1H, m, H-9‘), 2.19 (1H, m, H-3), 2.02 (3H, s, H-19), 1.99 (3H, s, H-19), 1.95 (1H, m, H-7‘), 1.69-1.53 (3H, m, H-7‘, H-8), 0.97-0.94 (6H, m, H-4) ppm; ^{13}C-NMR (100 MHz, $CDCl_3$, $CDCl_3$ = 77.16 ppm): δ = 172.4 (q, C-1), 171.8 (q, C-5), 167.5 (q, C-16), 157.5 (q, C-10), 145.8 (q, C-18), 136.6 (q, C-12), 128.7 (2C, t, C-14,), 128.29 (t, C-15), 128.28 (2C, t, C-13), 86.8 (q, C-17), 67.0 (s, C-11), 57.5 (t, C-2), 52.3 (p, C-20), 51.9 (t, C-6), 39.5 (s, C-9), 30.9 (s, C-7), 30.4 (t, C-3), 30.1 (p, C-19), 26.6 (s, C-8), 22.1 (p, C-19), 19.2 (p, C-4), 17.9 (p, C-4) ppm; HRMS (ESI): *m/z* calculated for $C_{24}H_{35}IN_3O_6$ $[M + H]^+$: 588.1571; found 588.1579.

Synthesis of compound 25 through intermediates S3-S5

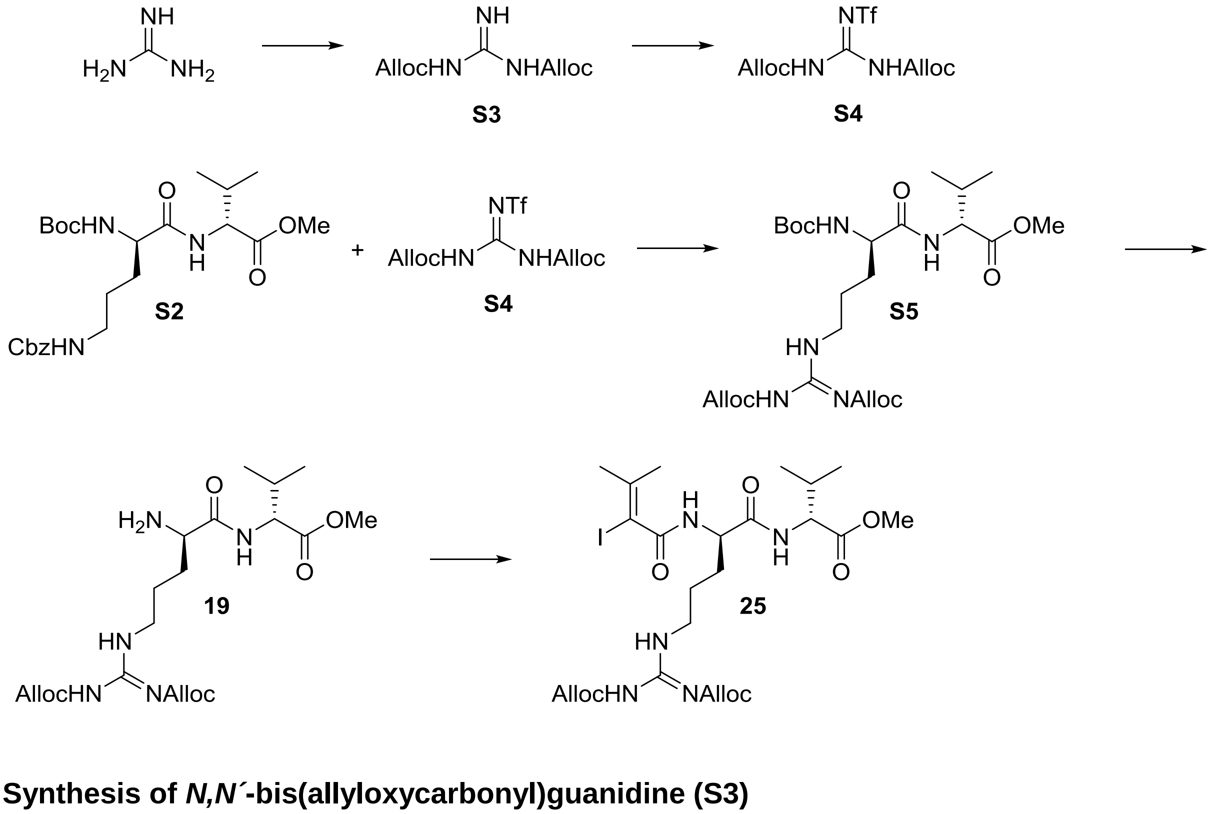

Synthesis of *N,N´*-bis(allyloxycarbonyl)guanidine (S3)

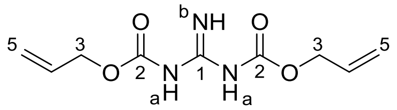

Guanidine hydrochloride (4.00 g, 41.9 mmol, 1.00 equiv) was dissolved in CH_2Cl_2 (80.0 mL) at rt. Then, NaOH (6.0 M; 28.0 mL) and $BnEt_3NCl$ (191 mg, 0.84 mmol, 20 mol %) were added. The reaction mixture was cooled to 0 °C and stirred for additional 15 min. Afterwards, allylchloroformiate (20.2 g, 167.5 mmol, 4.00 equiv) was added at 0 °C. The reaction mixture was stirred for additional 6.5 h at 0 °C. The resulting colourless solid was filtered and the filtrate was diluted with H_2O. The layers were separated and the aqueous phase was extracted with CH_2Cl_2 (3×). The combined organic layers were dried over $MgSO_4$, filtered and concentrated under reduced pressure. Purification by flash column chromatography on silica (CH_2Cl_2/Et_2O = 98:2 → CH_2Cl_2/Et_2O = 95:5 → CH_3OH/CH_2Cl_2 = 1:1) furnished guanidine **S3** (7.24 g, 31.8 mmol, 76%) as a colourless solid.

The spectroscopic and physical data were in accordance with those reported in the literature[S4].

R_f = 0.32 (CH_2Cl_2/Et_2O= 9:1); mp = 109 – 114 °C; ^{1}H-NMR (400 MHz, $CDCl_3$, $CHCl_3$ = 7.26 ppm): δ = 8.32 (2H, brs, NH-a), 5.91 (2H, ddt, *J* = 5.1, 11.0, 17.1 Hz, H-4), 5.33 (2H, dd, *J* = 11.0, 17.1 Hz, H-5), 5.25 (2H, dd, *J* = 11.0, 17.1 Hz, H-5), 4.61 (4H, d, *J* = 5.1 Hz, H-3) ppm; ^{13}C-NMR (100 MHz, $CDCl_3$, $CDCl_3$ = 77.16 ppm): δ = 158.9 (2C, q, C-2), 132.0 (2C, t, C-4), 118.7 (2C, s, C-5), 66.7 (2C, s, C-3) ppm; HRMS(ESI): *m/z* calculated for $C_9H_{14}N_3O_4$ $[M + H]^+$: 228.0984; found 228.0983.

The quaternary carbon atom C-1 was not detectable in the ^{13}C NMR spectra.

Synthesis of *N,N´*-bis(allyloxycarbonyl)-*N´´*-trifluoromethylsulfonylguanidine (S4)

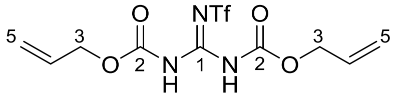

N,N´-Bis(allyloxycarbonyl)guanidine (**S3**; 1.64 g, 7.23 mmol, 1.0 equiv) was dissolved in CH_2Cl_2 (34.0 mL) at rt. The reaction mixture was cooled to −78 °C and stirred for 10 min. Then, freshly distilled Et_3N (1.10 g, 10.8 mmol, 1.50 equiv) was added dropwise and the mixture was stirred for additional 10 min at −78 °C. Subsequently, trifluoromethanesulfonic anhydride (3.05 g, 10.8 mmol, 1.50 equiv) was added dropwise while vigorously stirring over a period of 15 min at −78 °C. The reaction mixture was warmed up to rt and after 7 h the mixture was diluted with CH_2Cl_2 and washed with HCl (1.0 M; 3×). The combined organic layers were dried over $MgSO_4$, filtered and concentrated under reduced pressure. Purification by flash column chromatography over silica (CH_2Cl_2 = 100%) furnished title compound **S4** (1.32 g, 3.69 mmol, 51%) as a colourless oil.

The spectroscopic and physical data were in accordance with those reported in the literature[S4].

R_f = 0.31 (CH_2Cl_2= 100%); ^{1}H-NMR (400 MHz, $CDCl_3$, $CHCl_3$ = 7.26 ppm): δ = 10.30 (2H, brs, NH), 5.92 (2H, ddt, *J* = 6.0, 10.4, 17.1 Hz, H-4), 5.41 (2H, dd, *J* = 1.0, 1.4, 10.4 Hz, H-5),

5.36 (2H, dd, J = 1.0, 1.4, 10.4 Hz, H-5), 4.74 (4H, d, J = 6.0 Hz, H-3) ppm; ^{13}C-NMR (100 MHz, $CDCl_3$, $CDCl_3$ = 77.16 ppm): δ = 151.0 (2C, q, C-2), 150.0 (q, C-1), 130.2 (2C, t, C-4), 120.9 (2C, s, C-5), 117.7 (q, SO_2CF_3), 68.7 (2C, s, C-3) ppm; HRMS (ESI): *m/z* calculated for $C_{10}H_{12}F_3N_3O_6SNa$ $[M + Na]^+$: 382.0297; found 382.0298.

Synthesis of methyl (*E*)-*N*$^{\omega}$,*N*$^{\omega'}$-bis[(allyloxy)carbonyl]-N^2-(*tert*-butoxycarbonyl)-D-arginyl-D-valinate (S5)

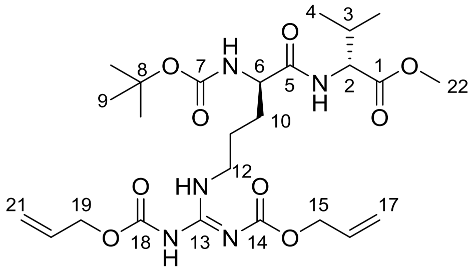

Dipeptide **S2** (225 mg, 0.47 mmol, 1.0 equiv) was dissolved in CH_3OH (18.0 mL) at rt. Then, palladium on charcoal (10%; 27.5 mg, 0.55 equiv) was added. The reaction mixture was flashed with hydrogen gas (3x) and stirred under an H_2 atmosphere at rt. After 20 h the reaction mixture was filtered over a pad of Celite™ and washed with CH_3OH. The solvent was removed under reduced pressure. The resulting residue was dissolved in CH_2Cl_2 (7.60 mL) at rt. Freshly distilled Et_3N (47.5 mg, 0.47 mmol, 1.00 equiv) was added dropwise and followed by compound **S4** (185 mg, 0.52 mmol, 1.10 equiv). After 21 h stirring at rt the solvent was removed under reduced pressure. Purification by flash chromatography (CH_2Cl_2/CH_3OH = 99:1) afforded title compound **S5** (248 mg, 0.45 mmol, 95% over two steps) as a colourless oil.

R_f = 0.28 (CH_2Cl_2/CH_3OH = 99:1); $[\alpha]_D^{20}$ = +4.5 (*c* 1.21, CH_2Cl_2); ^{1}H-NMR (400 MHz, CD_3OD, CD_3OH = 3.31 ppm): δ = 6.03-5.93 (2H, m, H-16, H-20), 5.40-5.19 (4H, m, H-17, H-21), 4.70 (2H, d, J = 5.8 Hz, H-19), 4.58 (2H, d, J = 5.8 Hz, H-15), 4.33 (1H, d, J = 6.4 Hz, H-2), 4.11 (1H, m, H-6), 3.71 (3H, s, H-22), 3.43 (2H, m, H-12), 2.15 (1H, sept, J = 6.4 Hz, H-3), 1.82-1.59 (4H, m, H-10, H-11), 1.44 (9H, s, H-9), 0.95 (6H, d, J = 6.4 Hz, H-4) ppm; ^{13}C-NMR (100 MHz, CD_3OD, CD_3OD = 49.00 ppm): δ = 175.1 (q, C-5), 173.4 (q, C-1), 164.8 (q, C-13), 157.6 (2C, q, C-7, C-14), 154.7 (q, C-18), 134.4 (t, C-16), 132.8 (t, C-20), 119.5 (s, C-21), 117.9 (s, C-17), 80.6 (q, C-8), 68.1 (s, C-19), 67.3 (s, C-15), 59.0 (t, C-2), 55.5 (t, C-6), 52.5 (p, C-22), 41.6 (s, C-12), 31.9 (t, C-3), 30.4 (s, C-10), 28.7 (3C, p, C-9), 26.6 (s, C-11), 19.4 (p, C-4), 18.4 (p, C-4) ppm; HRMS(ESI): *m/z* calculated for $C_{25}H_{41}N_5O_9Na$ $[M + Na]^+$: 578.2802; found 578.2796.

Synthesis of methyl (*E*)-N^{ω},$N^{\omega'}$-bis[(allyloxy)carbonyl)-N^{2}-(2-iodo-3-methylbut-2-enoyl)-D-arginyl-D-valinate (25)

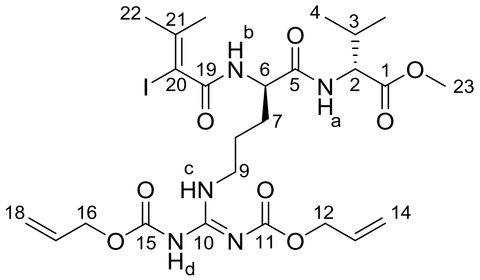

Boc-protected dipeptide **S5** (611 mg, 1.10 mmol, 1.0 equiv) was dissolved in CH_2Cl_2 (13.5 mL) at 0 °C. Afterwards, TFA (5.31 g, 46.6 mmol, 50.0 equiv) was added dropwise. After 2.5 h the reaction was terminated by addition of a saturated sodium bicarbonate solution. The layers were separated and the aqueous layer was extracted with EtOAc (5×). The combined organic layers were dried over $MgSO_4$, filtered and concentrated under reduced pressure. The desired product **19** (501 mg, 1.10 mmol, quantitative) was obtained as a colourless foam.

^{1}H-NMR (200 MHz, CD_3OD, CD_3OH = 3.31 ppm): δ = 5.99 (2H, m, H-13, H-17), 5.43-5.18 (4H, m, H-14, H-18), 4.70 (2H, dt, *J* = 1.3, 5.8 Hz, H-16), 4.59 (2H, dt, *J* = 1.4, 5.6 Hz, H-12), 4.36 (1H, d, *J* = 5.7 Hz, H-6), 3.79 (1H, t, *J* = 6.6 Hz, H-2), 3.71 (3H, s, OCH_3), 3.43 (2H, m, H-9), 2.19 (1H, m, H-3), 1.85-1.69 (4H, m, H-7, H-8), 0.97 (6H, d, *J* = 6.6 Hz, H-4) ppm; HRMS (ESI): *m/z* calculated for $C_{20}H_{34}N_5O_7$ $[M + H]^+$: 456.2458; found 456.2451.

Acid **10** (219 mg, 0.97 mmol, 1.0 equiv) and dipeptide **19** (486 mg, 1.07 mmol, 1.10 equiv) were dissolved in DMF (5.30 mL) at 0 °C. Then, HOAt (141 mg, 1.04 mmol, 1.07 equiv) and PyAOP (530 mg, 1.02 mmol, 1.05 equiv) were added. Afterwards, DIPEA (575 mg, 4.45 mmol, 4.60 equiv) was added dropwise. The reaction mixture was warmed up to rt. After 23 h, the reaction was terminated by addition of a saturated NH_4Cl solution. The layers were separated and the aqueous layer was extracted with EtOAc (7×). The combined organic layers were washed with brine and subsequently dried over Na_2SO_4, filtered and concentrated under reduced pressure. Purification by flash column chromatography over silica (petroleum ether/ ethyl acetate = 3:1 → petroleum ether/ ethyl acetate = 1:1) gave the desired vinyl iodide **25** (576 mg, 0.87 mmol, 90%) as a colourless solid.

R_f = 0.24 (CH_2Cl_2/CH_3OH 98:2); mp = 127 – 132 °C; $[\alpha]_D^{20}$ = +11.7 (*c* = 2.47, CH_2Cl_2); ^{1}H-NMR (400 MHz, $CDCl_3$, $CHCl_3$ = 7.26 ppm): δ = 11.71 (1H, brs, NH-d), 8.38 (1H, t, *J* = 5.5 Hz, NH-c), 6.62 (1H, d, *J* = 8.5 Hz, NH-a), 6.56 (1H, d, *J* = 8.2 Hz, NH-b), 6.01-5.85 (2H, m, H-13, H-17), 5.38-5.21 (4H, m, H-14, H-18), 4.64 (2H, m, H-16), 4.58 (2H, m, H-12), 4.55-4.48 (2H, m, H-2, H-6), 3.72 (3H, s, H-23), 3.49 (2H, m, H-9), 2.16 (1H, m, H-3), 2.01 (3H, s, H-22), 1.99 (3H, s, H-22), 1.95 (1H, m, H-7), 1.79-1.68 (3H, m, H-7‘, H-8), 0.93 (3H, d, *J* =

6.8 Hz, H-4), 0.90 (3H, d, *J* = 6.8 Hz, H-4) ppm; ^{13}C-NMR (100 MHz, $CDCl_3$, $CDCl_3$ = 77.16 ppm): δ = 172.0 (q, C-1), 171.0 (q, C-5), 167.7 (q, C-19), 163.6 (q, C-10), 156.4 (q, C-11), 153.9 (q, C-15), 146.3 (q, C-21), 133.1 (t, C-13), 131.1 (t, C-17), 119.6 (s, C-18), 118.1 (s, C-14), 86.1 (q, C-20), 67.2 (s, C-16), 66.4 (s, C-12), 57.5 (t, C-2), 53.5 (t, C-6), 52.3 (p, C-23), 40.5 (s, C-9), 31.2 (t, C-3), 30.1 (p, C-22), 29.1 (s, C-7), 25.5 (s, C-8), 22.1 (p, C-22), 19.1 (p, C-4), 17.9 (p, C-4) ppm; HRMS (ESI): *m/z* calculated for $C_{25}H_{39}IN_5O_8$ [M + H]$^+$: 664.1843; found 664.1842.

Synthesis of compound 20 through intermediates S6-S9

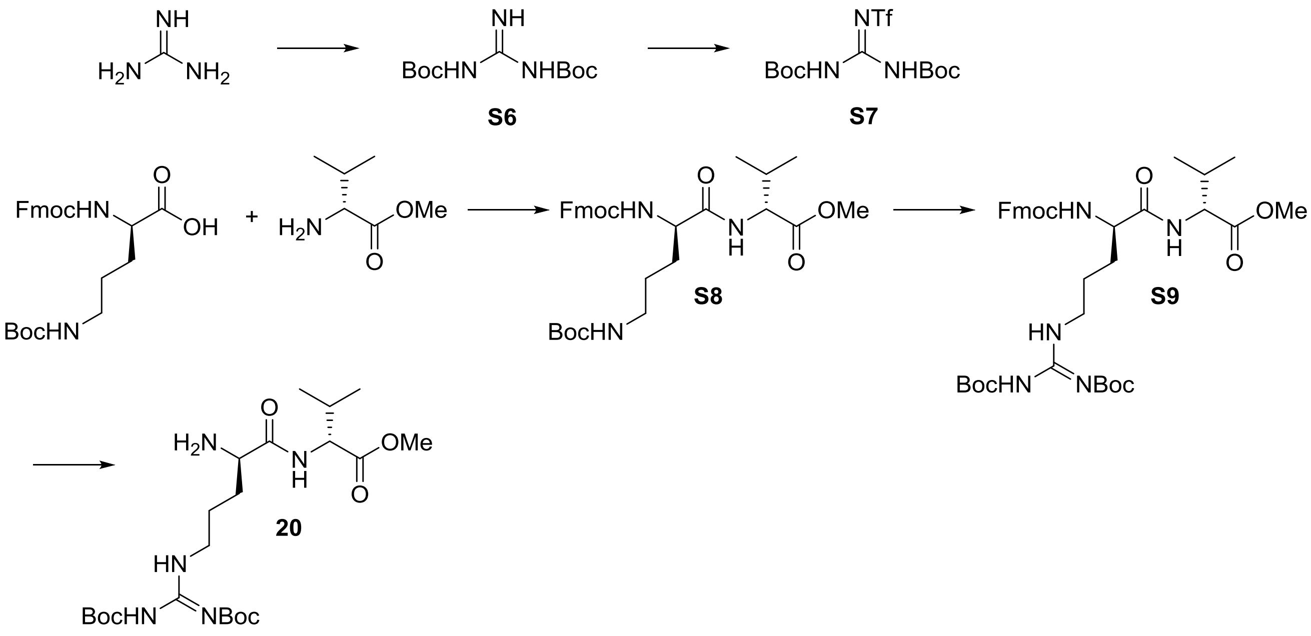

Synthesis of *N,N´*-bis(*tert*-butoxycarbonyl)guanidine (S6)

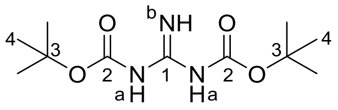

Guanidine hydrochloride (4.00 g, 41.9 mmol, 1.00 equiv) and NaOH (6.70 g, 167 mmol, 4.00 equiv) were dissolved in a mixture of 1,4-dioxane/H_2O (2:1; 126 mL) at 0 °C. Afterwards, $(Boc)_2O$ (20.1 g, 92.1 mmol, 2.20 equiv) was added. The reaction mixture was warmed to rt and stirred for 21 h. Then, the solvent was removed under reduced pressure. The resulting residue was diluted with H_2O and extracted with EtOAc (3×). The combined organic layers were washed with citric acid (10%) as well as H_2O and brine. Subsequently, the combined organic layers were dried over $MgSO_4$ and filtered. The solvent was removed under reduced pressure. Purification by flash column chromatography over silica (CH_2Cl_2/CH_3OH = 97:3) gave the desired product **S6** (5.56 g, 21.4 mmol, 51%) as colouress solid. The spectroscopic and physical data were in accordance with those reported in the literature[S5].

R_f = 0.29 (CH_2Cl_2/MeOH 97:3); mp= 135 °C (lit.[S5]: mp= 144 °C); ^{1}H-NMR (400 MHz, DMSO-d_6, DMSO-d_5 = 2.50 ppm): δ = 10.42 (1H, brs, NH-b), 8.47 (2H, brs, NH-a), 1.41 (18H, s, H-4) ppm; ^{13}C-NMR (100 MHz, DMSO-d_6, DMSO-d_6 = 39.52 ppm): δ = 158.5 (3C, q, C-1, C-2), 79.6 (2C, q, C-3), 27.8 (6C, p, C-4) ppm; HRMS (ESI): *m/z* calculated for $C_{11}H_{22}N_3O_4$ [M + H]$^+$: 260.1610; found 260.1609.

Synthesis of *N,N´*-bis(*tert*-butoxycarbonyl)-*N´´*-trifluoromethansulfonylguanidine (S7)

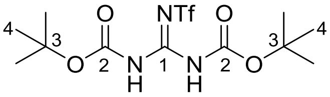

N,N´-Bis(*tert*-butoxycarbonyl)guanidine (**S6**; 4.38 g, 16.9 mmol, 1.00 equiv) was dissolved in CH_2Cl_2 (84.4 mL) at rt. The reaction mixture was cooled to −78 °C and stirred for 10 min. Then, freshly distilled Et_3N (1.79 g, 17.7 mmol, 1.05 equiv) was added dropwise. Subsequently, trifluoromethanesulfonic anhydride (5.00 g, 17.7 mmol, 1.05 equiv) was added dropwise while vigorously stirring over a period of 15 min at −78 °C. The reaction mixture was warmed to rt. After 6 h, the mixture was washed with a $KHSO_4$ solution (1.0 M), followed by H_2O. The combined organic layers were dried over Na_2SO_4 and filtered. The solvent was removed under reduced pressure. Purification by flash column chromatography over silica (CH_2Cl_2 = 100%) furnished the title compound **S7** (5.51 g, 14.1 mmol, 83%) as a colourless solid. The spectroscopic and physical data were in accordance with those reported in the literature[S5].

R_f = 0.74 (100% CH_2Cl_2); mp = 115 °C (lit.[S5]: mp = 115 °C); ^{1}H-NMR (400 MHz, DMSO-d_6, DMSO-d_5 = 2.50 ppm): δ = 11.06 (2H, brs, NH), 1.46 (18H, s, H-4) ppm; ^{13}C-NMR (100 MHz, DMSO-d_6, DMSO-d_6 = 39.52 ppm): δ = 152.3 (2C, q, C-2), 150.1 (q, C-1), 120.6 (q, d, *J* = 320.2 Hz, SO_2CF_3), 83.4 (2C, q, C-3), 27.5 (6C, p, C-4) ppm; HRMS (ESI): *m/z* calculated for $C_{12}H_{20}F_3N_3O_6SNa$ [M + Na]$^+$: 414.0923; found 414.0923.

Synthesis of methyl [(*R*)-2-{[((9*H*-fluorene-9-yl)methoxy)carbonyl]amino}-5-[(*tert*-butoxycarbonyl)amino]pentanoyl]-D-valinate (S8)

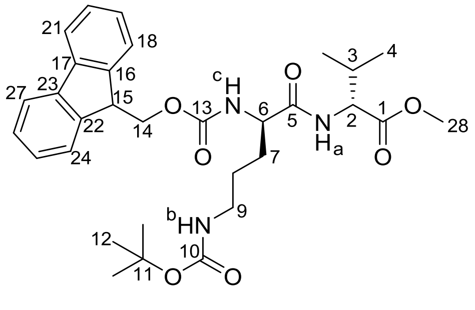

Fmoc-D-Orn(Boc)-OH (500 mg, 1.10 mmol, 1.0 equiv) and D-valine methylester hydrochloride (221 mg, 1.32 mmol, 1.20 equiv) were dissolved in CH_2Cl_2 (50.0 mL) at 0 °C. Then, HOAt (225 mg, 1.65 mmol, 1.50 equiv), EDC·HCl (316 mg, 1.65 mmol, 1.50 equiv) and

$NaHCO_3$ (490 mg, 5.50 mmol, 5.0 equiv) were added. The reaction mixture was stirred at 0 °C → rt. After 22 h, the solvent was removed under reduced pressure. Purification by flash chromatography (CH_2Cl_2/CH_3OH 99:1 → CH_2Cl_2/CH_3OH 97:3) furnished the desired dipeptide **S8** (615 mg, 1.08 mmol, 98%) as colourless foam.

R_f = 0.24 (CH_2Cl_2/CH_3OH 99:1); $[\alpha]_D^{20}$ = -1.8 (*c* = 1.44, CH_2Cl_2); ^{1}H-NMR (400 MHz, $CDCl_3$, $CHCl_3$ = 7.26 ppm): δ = 7.76 (2H, d, *J* = 7.5 Hz, H-21, H-27), 7.60 (2H, d, *J* = 7.5 Hz, H-18, H-24), 7.40 (2H, t, *J* = 7.5 Hz, H-20, H-26), 7.30 (2H, t, *J* = 7.5 Hz, H-19, H-25), 6.69 (1H, brs, NH-a), 5.62 (1H, brs, NH-c), 4.70 (1H, brs, NH-b), 4.51 (1H, dd, *J* = 6.5, 8.9 Hz, H-2), 4.41-4.35 (3H, m, H-6, H-14), 4.22 (1H, t, *J* = 7.00 Hz, H-15), 3.72 (3H, s, H-28), 3.28 (1H, m, H-9), 3.11 (1H, m, H-9'), 2.19 (1H, m, H-3), 1.90 (1H, m, H-7), 1.69-1.52 (3H, m, H-7', H-8), 1.44 (9H, s, H-12), 0.92 (6H, d, *J* = 6.5 Hz, H-4) ppm; ^{13}C-NMR (100 MHz, $CDCl_3$, $CDCl_3$ = 77.16 ppm): δ = 172.3 (q, C-1), 172.0 (q, C-5), 156.7 (q, C-13), 156.4 (q, C-10), 144.0 (q, C-15 o. C-22), 143.9 (q, C-15 o. C-22), 141.5 (q, C-17 o. C-23), 141.4 (q, C-17 o. C-23), 127.9 (2C, t, C-19 o. C-20 o. C-25 o. C-26), 127.2 (2C, t, C-19 o. C-20 o. C-25 o. C-26), 125.3 (2C, t, C-18, C-24), 120.12 (t, C-21 o. C-27), 120.10 (t, C-21 o. C-27), 79.5 (q, C-11), 67.2 (s, C-14), 57.5 (t, C-2), 54.0 (t, C-6), 52.3 (p, C-28), 47.3 (t, C-15), 31.0 (t, C-3), 30.1 (s, C-7), 28.6 (3C, p, C-12), 26.5 (s, C-8), 19.2 (p, C-4), 18.0 (p, C-4) ppm; HRMS (ESI): *m/z* calculated for $C_{31}H_{41}N_3O_7Na$ $[M + Na]^+$: 590.2842; found 590.2841.

The carbon atom C-9 could not be detected in the ^{13}C NMR spectrum.

Synthesis of methyl (*E*)-*N*^{2}-{[(9*H*-fluorene-9-yl)methoxy]carbonyl}-*N*$^\omega$,*N*$^{\omega'}$-bis(*tert*-butoxycarbonyl)-D-arginyl-D-valinate (S9)

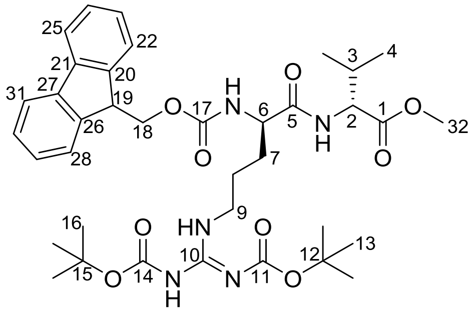

Boc-protected dipeptide **S8** (260 mg, 0.46 mmol, 1.00 equiv) was dissolved in CH_2Cl_2 (4.60 mL) at 0 °C. Afterwards, TFA (2.61 g, 22.9 mmol, 50.0 equiv) was added dropwise. After 2.5 h, the reaction was terminated by addition of a saturated sodium bicarbonate solution. The layers were separated and the aqueous layer was extracted with EtOAc (6×). The combined organic layers were washed with brine and then dried over $MgSO_4$ as well as filtered. The solvent was removed under reduced pressure. The product was used without further purification.

The crude product was dissolved in CH_2Cl_2 (4.60 mL) at rt. Then, $NaHCO_3$ (204 mg, 2.29 mmol, 5.0 equiv) and guanidine derivative **S7** (179 mg, 0.46 mmol, 1.00 equiv) were

added. The reaction mixture was stirred for 5 d at rt. Afterwards, the solvent was removed under reduced pressure. Purification by flash chromatography over silica (CH_2Cl_2/CH_3OH 99:1 → CH_2Cl_2/CH_3OH 98:2) gave the desired product **S9** (175 mg, 0.25 mmol, 54% over two steps) as colourless foam.

R_f = 0.65 (CH_2Cl_2/CH_3OH 95:5); $[\alpha]_D^{20}$ = -2.2 (*c* = 0.87, CH_2Cl_2); ^{1}H-NMR (400 MHz, CD_3OD, CD_3OH = 3.31 ppm): δ = 7.75 (2H, d, *J* = 7.5 Hz, H-25, H-31), 7.63 (2H, t, *J* = 7.5 Hz, H-22, H-28), 7.36 (2H, t, *J* = 7.5 Hz, H-24, H-30), 7.27 (2H, t, *J* = 7.5 Hz, H-23, H-29), 4.40-4.31 (3H, m, H-2, H-20), 4.24-4.17 (2H, m, H-6, H-19), 3.68 (3H, s, H-32), 3.37 (2H, m, H-9), 2.13 (1H, m, H-3), 1.80 (1H, m, H-7), 1.70-1.59 (3H, m, H-7‘, H-8), 1.49 (9H, s, H-13 o. H-16), 1.46 (9H, s, H-13 o. H-16), 0.93 (6H, d, *J* = 6.6 Hz, H-4) ppm; ^{13}C-NMR (100 MHz, CD_3OD, CD_3OD = 49.00 ppm): δ = 174.8 (q, C-1), 173.3 (q, C-5), 164.4 (q, C-10), 158.3 (q, C-17), 157.6 (q, C-11 o. C-14), 154.1 (q, C-11 o. C-14), 145.3 (q, C-20 o. C-26), 145.1 (q, C-20 o. C-26), 142.6 (q, C-21 o. C-27), 142.5 (q, C-21 o. C-27), 128.7 (2C, t, C-23 o. C-24 o. C-29 o. C-30), 128.1 (2C, t, C-23 o. C-24 o. C-29 o. C-30), 126.2 (2C, t, C-22, C-28), 120.9 (2C, t, C-25, C-31), 84.4 (q, C-12 o. C-15), 80.4 (q, C-12 o. C-15), 67.8 (s, C-18), 59.1 (t, C-2), 55.8 (t, C-6), 52.5 (p, C-32), 48.4 (t, C-19), 41.3 (s, C-9), 31.7 (t, C-3), 30.5 (s, C-7), 28.6 (3C, p, C-13 o. C-16), 28.2 (3C, p, C-13 o. C-16), 26.7 (s, C-8), 19.5 (p, C-4), 18.5 (p, C-4) ppm; HRMS (ESI): *m/z* calculated for $C_{37}H_{52}N_5O_9$ $[M + H]^+$: 710.3765; found 710.3764.

Synthesis of methyl (*E*)-N^{ω},$N^{\omega'}$-bis(*tert*-butoxycarbonyl)-D-arginyl-D-valinate (20)

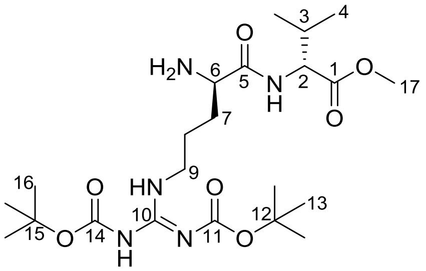

Dipeptide **S9** (106 mg, 0.15 mmol, 1.00 equiv) was dissolved in CH_2Cl_2 (11.0 mL) at 0 °C. Afterwards, tris(2-aminoethyl)amine (1.10 mL) was added dropwise. The reaction mixture was warmed to rt and stirred for additional 3 h. The reaction was terminated by addition of H_2O. The layers were separated and the organic layer was washed with H_2O again. The combined organic layers were washed with brine, dried over $MgSO_4$ and filtered. The solvent was removed under reduced pressure. Purification by flash chromatography over silica (CH_2Cl_2/CH_3OH 98:2 → CH_2Cl_2/CH_3OH 96:4 → CH_2Cl_2/CH_3OH 94:6) yielded amine **20** (65.5 mg, 0.13 mmol, 90%) as a colourless oil.

R_f = 0.58 (CH_2Cl_2/CH_3OH 9:1); $[\alpha]_D^{20}$ = -1.4 (*c* = 1.31, CH_3OH); ^{1}H-NMR (400 MHz, CD_3OD, CD_3OH = 3.31 ppm): δ = 4.33 (1H, d, *J* = 6.6 Hz, H-2), 3.72 (3H, s, H-17), 3.45-3.37 (3H, m, H-6, H-9), 2.17 (1H, m, H-3), 1.76-1.59 (4H, m, H-7, H-8), 1.52 (9H, s, H-13 o. H-16), 1.47 (9H, s, H-13 o. H-16), 0.97 (6H, d, *J* = 6.6 Hz, H-4) ppm; ^{13}C-NMR (100 MHz, CD_3OD,

CD_3OD = 49.00 ppm): δ = 177.6 (q, C-5), 173.4 (q, C-1), 164.6 (q, C-10), 157.6 (q, C-11 o. C-14), 154.1 (q, C-11 o. C-14), 84.4 (q, C-12 o. C-15), 80.3 (q, C-12 o. C-15), 59.1 (t, C-2), 55.4 (t, C-6), 52.5 (p, C-17), 41.4 (s, C-9), 33.7 (t, C-3), 31.7 (s, C-7), 28.6 (3C, p, C-13 o. C-16), 28.2 (3C, p, C-13 o. C-16), 26.3 (s, C-8), 19.5 (p, C-4), 18.5 (p, C-4) ppm; HRMS (ESI): *m/z* calculated for $C_{22}H_{42}N_5O_7$ [M + H]$^+$: 488.3084; found 488.3081.

Synthesis of methyl (*E*)-*N*$^{\omega}$,*N*$^{\omega'}$-bis(*tert*-butoxycarbonyl)-*N*^{2}-(2-iodo-3-methylbut-2-enoyl)-D-arginyl-D-valinate (26)

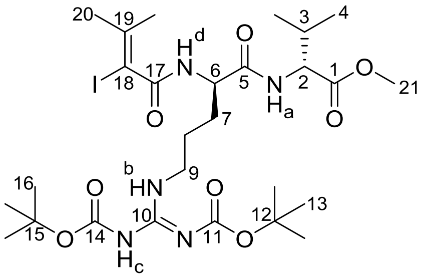

Acid **10** (64.2 mg, 0.28 mmol, 1.20 equiv) and amine **20** (100 mg, 0.26 mmol, 1.0 equiv) were dissolved in DMF (2.60 mL) at 0 °C. Then, HOAt (37.6 mg, 0.28 mmol, 1.07 equiv) and PyAOP (141 mg, 0.27 mmol, 1.05 equiv) were added. Afterwards, DIPEA (153 mg, 1.18 mmol, 4.60 equiv) was added dropwise. The reaction mixture was stirred at 0 °C → rt. After 22 h, the reaction was terminated by addition of a saturated NH_4Cl solution. The layers were separated and the aqueous layer was extracted with EtOAc (7×). The combined organic layers were washed with brine and subsequently dried over $MgSO_4$, filtered and concentrated under reduced pressure. Purification by flash column chromatography over silica (petroleum ether/ ethyl acetate = 3:1 → petroleum ether/ethyl acetate = 1:1) gave the title compound **26** (108 mg, 0.15 mmol, 60%) as a colourless oil.

^{1}H-NMR (400 MHz, $CDCl_3$, $CHCl_3$ = 7.26 ppm): δ = 11.47 (1H, s, NH-c), 8.41 (1H, s, NH), 6.62 (1H, d, *J* = 8.5 Hz, NH), 6.43 (1H, d, *J* = 7.2 Hz, NH), 4.51 (2H, m, H-2, H-6), 3.74 (3H, s, H-21), 3.48 (2H, m, H-9), 2.18 (1H, m, H-3), 2.03 (3H, s, H-20), 2.00 (3H, s, H-20), 1.98 (1H, m, H-7), 1.77-1.59 (3H, m, H-7‘, H-8), 1.49 (18H, s, H-13, H-16), 0.95-0.92 (6H, m, H-4) ppm; HRMS (ESI): *m/z* calculated for $C_{27}H_{47}IN_5O_8$ [M + H]$^+$: 696.2469; found 696.2458.

Synthesis of compound 21 through intermediate S10

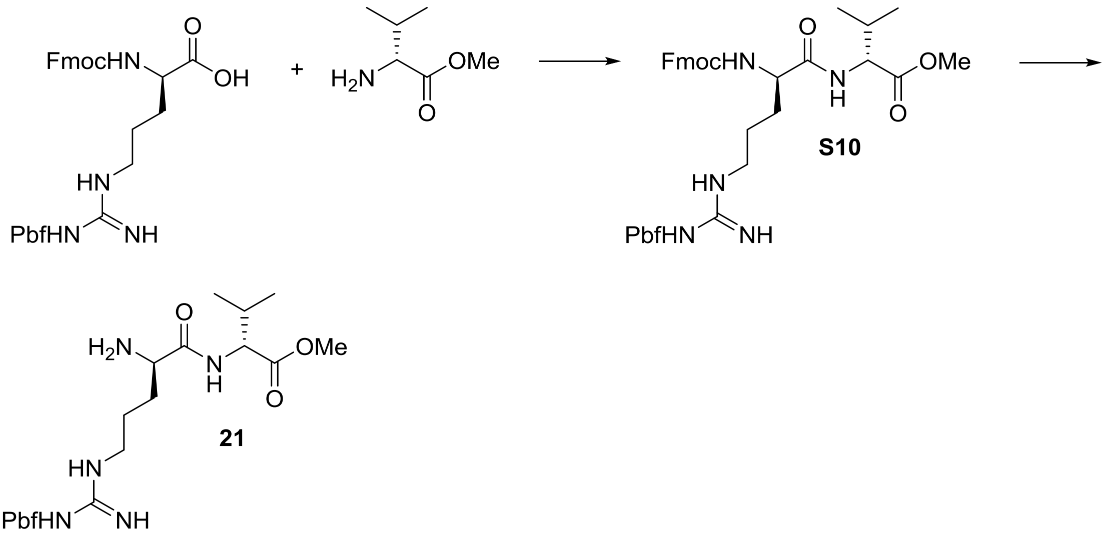

Synthesis of methyl N^2-{[(9*H*-fluorene-9-yl)methoxy]carbonyl}-N^ω-[(2,2,4,6,7-pentamenthyl-2,3-dihydrobenzofurane-5-yl)sulfonyl]-D-arginyl-D-valinate (S10)

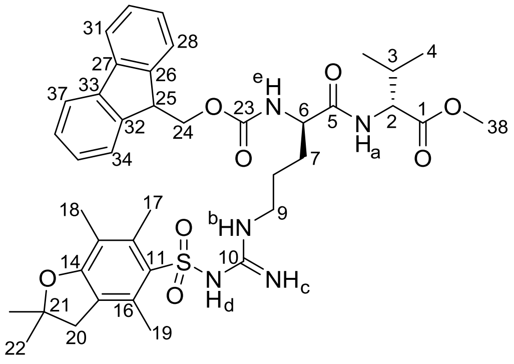

Fmoc-D-Arg(Pbf)-OH (2.00 g, 3.08 mmol, 1.00 equiv) und D-valine methylester hydrochloride (620 mg, 3.70 mmol, 1.20 equiv) were dissolved in CH_2Cl_2 (140 mL) at 0 °C. Then, HOAt (629 mg, 4.62 mmol, 1.50 equiv), EDC·HCl (886 mg, 4.62 mmol, 1.50 equiv) and $NaHCO_3$ (1.37 g, 15.4 mmol, 5.00 equiv) were added. The reaction mixture was stirred at 0 °C → rt. After 22 h, the solvent was removed under reduced pressure. Purification by flash chromatography over silica (CH_2Cl_2/CH_3OH 99:1 → CH_2Cl_2/CH_3OH 97:3 → CH_2Cl_2/CH_3OH 96:4) gave the desired dipeptide **S10** (2.53 g, with impurities of D-Val-OMe) as a colourless foam. The product was used without further purification.

R_f = 0.20 (CH_2Cl_2/CH_3OH 98:2); ^{1}H-NMR (400 MHz, $CDCl_3$, $CHCl_3$ = 7.26 ppm): δ = 7.71 (2H, d, *J* = 7.3 Hz, H-31, H-37), 7.54 (2H, d, *J* = 7.3 Hz, H-28, H-34), 7.34 (3H, t, *J* = 7.3 Hz, H-30, H-36, NH-a), 7.21 (2H, t, *J* = 7.3 Hz, H-29, H-35), 6.40 (2H, s, NH), 6.24 (1H, d, *J* = 7.2 Hz, NH-e), 4.40 (2H, m, H-2, H-6), 4.30 (2H, m, H-24), 4.12 (1H, m, H-25), 3.64 (3H, s, H-38), 3.24 (2H, m, H-9), 2.88 (2H, s, H-20), 2.58 (3H, s, H-17 o. H-19), 2.50 (3H, s, H-17 o. H-19), 2.11 (1H, m, H-3), 2.05 (3H, s, H-18), 1.90 (1H, m, H-7), 1.75 (1H, m, H-7‘), 1.61 (2H, m, H-

8), 1.40 (6H, s, H-22), 0.86 (6H, m, H-4) ppm; HRMS (ESI): *m/z* calculated for $C_{40}H_{51}N_5O_8SNa$ [M + Na]$^+$: 784.3356; found 784.3350.

Synthesis of methyl *N*$^{\omega}$-[(2,2,4,6,7-pentamethyl-2,3-dihydrobenzofurane-5-yl)sulfonyl]-D-arginyl-D-valinate (21)

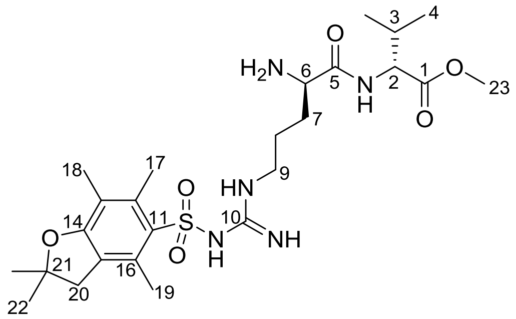

Dipeptide **S10** (800 mg, 1.05 mmol, 1.0 equiv) was dissolved in CH_2Cl_2 (70.0 mL) at 0 °C. Then, tris(2-aminoethyl)amine (7.00 mL) was added dropwise. The reaction mixture was warmed to rt and stirred for 2.5 h. The reaction was terminated by adding H_2O. The layers were separated and the organic layer was washed with H_2O (2×). Afterwards, the combined organic layers were washed with brine and then dried over $MgSO_4$ as well as filtered. The solvent was removed under reduced pressure. Purification by flash chromatography over silica (CH_2Cl_2/CH_3OH 98:2 → CH_2Cl_2/CH_3OH 96:4 → CH_2Cl_2/CH_3OH 94:6 → CH_2Cl_2/CH_3OH 92:8 → CH_2Cl_2/CH_3OH 9:1) gave the desired amine **21** (535 mg, 0.99 mmol, 94% over two steps) as a colourless foam.

R_f = 0.33 (CH_2Cl_2/CH_3OH 9:1); $[\alpha]_D^{20}$ = +1.4 (*c* = 1.03, CH_3OH); ^{1}H-NMR (400 MHz, CD_3OD, CD_3OH = 3.31 ppm): δ = 4.31 (1H, d, *J* = 6.6 Hz, H-2), 3.70 (3H, s, H-23), 3.40 (1H, m, H-6), 3.17 (2H, m, H-9), 2.99 (3H, s, H-20), 2.57 (3H, s, H-17 o. H-19), 2.51 (3H, s, H-17 o. H-19), 2.15 (1H, m, H-3), 2.08 (3H, s, H-18), 1.67 (1H, m, H-7), 1.54 (3H, m, H-7‘, H-8), 1.45 (6H, s, H-22), 0.95 (6H, d, *J* = 6.6 Hz, H-4) ppm; ^{13}C-NMR (100 MHz, CD_3OD, CD_3OD = 49.0 ppm): δ = 177.5 (q, C-5), 173.5 (q, C-1), 159.8 (q, C-14), 158.0 (q, C-10), 139.4 (q, C-11), 134.4 (q, C-12 o. C-13 o. C-16), 133.5 (q, C-15), 126.0 (q, C-12 o. C-13 o. C-16), 118.4 (q, C-12 o. C-13 o. C-16), 87.6 (q, C-21), 59.1 (t, C-2), 55.3 (t, C-6), 52.5 (p, C-23), 43.9 (s, C-20), 41.7 (s, C-9), 33.5 (t, C-3), 31.7 (s, C-7), 28.7 (2C, p, C-22), 26.6 (s, C-8), 19.6 (p, C-4 o. C-17 o. C-19), 19.5 (p, C-4 o. C-17 o. C-19), 18.5 (p, C-4 o. C-17 o. C-19), 18.4 (p, C-4 o. C-17 o. C-19), 12.5 (p, C-18) ppm; HRMS (ESI): *m/z* calculated for $C_{25}H_{42}N_5O_6S$ [M + H]$^+$: 540.2856; found 540.2855.

Synthesis of methyl *N*²-(2-iodo-3-methylbut-2-enoyl)-*N*ω-[(2,2,4,6,7-pentamethyl-2,3-dihydrobenzofurane-5-yl)sulfonyl]-D-arginyl-D-valinate (27)

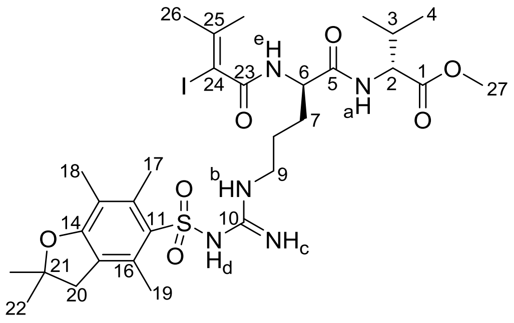

Acid **10** (6114 mg, 0.50 mmol, 1.20 equiv) and amine **21** (227 mg, 0.42 mmol, 1.0 equiv) were dissolved in DMF (4.20 mL) at 0 °C. Then, HOAt (61.2 mg, 0.45 mmol, 1.07 equiv) and PyAOP (230 mg, 0.44 mmol, 1.05 equiv) were added. Afterwards, DIPEA (249 mg, 1.93 mmol, 4.60 equiv) was added dropwise. The reaction mixture was stirred at 0 °C → rt. After 22 h, the reaction was terminated by addition of a saturated NH_4Cl solution. The layers were separated and the aqueous layer was extracted with EtOAc (6×). The combined organic layers were washed with brine and subsequently dried over $MgSO_4$, filtered as well as concentrated under reduced pressure. Purification by flash column chromatography over silica (petroleum ether/ ethyl acetate = 2:1 → petroleum ether/ethyl acetate = 1:1 → 100% ethyl acetate → ethyl acetate/CH_3OH = 20:1) gave the title compound **27** (250 mg, 0.33 mmol, 80%) as a colourless oil.

R_f = 0.11 (CH_2Cl_2/CH_3OH 98:2); $[\alpha]_D^{20}$ = +10.8 (*c* = 1.02, CH_2Cl_2); ^{1}H-NMR (400 MHz, $CDCl_3$, $CHCl_3$ = 7.26 ppm): δ = 7.37 (1H, d, *J* = 7.9 Hz, NH-a), 7.07 (1H, d, *J* = 7.9 Hz, NH-e), 6.34 (2H, s, NH-b, NH-d), 4.62 (1H, m, H-6), 4.38 (1H, dd, *J* = 5.5, 7.9 Hz, H-2), 3.69 (3H, s, H-27), 3.27 (2H, brm, H-9), 2.94 (2H, s, H-20), 2.56 (3H, s, H-17 o. H-19), 2.49 (3H, s, H-17 o. H-19), 2.14 (1H, m, H-3), 2.08 (3H, s, H-18), 1.99 (1H, m, H-7), 1.96 (3H, s, H-26), 1.91 (3H, s, H-26), 1.79 (1H, m, H-7‘), 1.63 (2H, m, H-8), 1.45 (6H, s, H-22), 0.90 (6H, m, H-4) ppm; ^{13}C-NMR (100 MHz, $CDCl_3$, $CDCl_3$ = 77.16 ppm): δ = 172.4 (q, C-1), 171.9 (q, C-5), 168.2 (q, C-23), 158.9 (q, C-14), 156.5 (q, C-10), 145.2 (q, C-25), 138.5 (q, C-11), 132.9 (q, C-12 o. C-13 o. C-15 o. C-16), 132.4 (q, C-12 o. C-13 o. C-15 o. C-16), 124.7 (q, C-12 o. C-13 o. C-16), 117.6 (q, C-12 o. C-13 o. C-16), 86.5 (q, C-21), 86.3 (q, C-24), 58.1 (t, C-2), 53.3 (t, C-6), 52.3 (p, C-27), 43.4 (s, C-20), 40.8 (s, C-9), 30.7 (t, C-3), 29.71 (s, C-7), 29.66 (p, C-26), 28.7 (2C, p, C-22), 25.5 (s, C-8), 22.0 (p, C-26), 19.5 (p, C-17 o. C-19), 19.2 (p, C-4), 18.3 (p, C-4), 18.1 (p, C-17 o. C-19), 12.6 (p, C-18) ppm; HRMS (ESI): *m/z* calculated for $C_{30}H_{46}IN_5O_7SNa$ $[M + Na]^+$: 770.2060; found 770.2052.

Signals of NH^c-proton could not be detected in the ^{1}H NMR spectrum.

Synthesis of compound 23 through intermediate S11

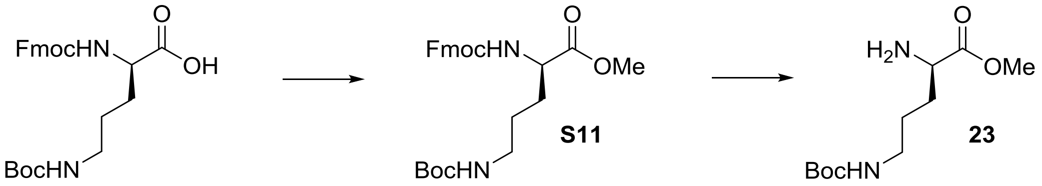

Synthesis of methyl (*R*)-2-{[((9*H*-fluorene-9-yl)methoxy)carbonyl]amino}-5-[(*tert*-butoxycarbonyl)amino]pentanoate (S11)

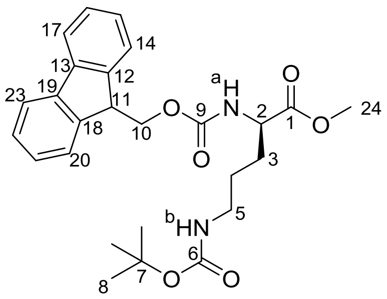

Fmoc-D-Orn(Boc)-OH (2.00 g, 5.50 mmol, 1.00 equiv) was dissolved in toluene (22.0 mL) and CH_3OH (8.80 mL) at 0 °C. Then, $TMSCHN_2$ (2.0 M in Et_2O; 3.00 mL, 7.15 mmol, 1.30 equiv) was added dropwise. Now, the reaction mixture was stirred at 0 °C → rt. After 22 h, the solvent was removed under reduced pressure. The resulting residue was diluted with CH_2Cl_2 and afterwards washed with a $NaHCO_3$ solution (5%). The layers were separated and the aqueous layer was washed with CH_2Cl_2 (4×). The combined organic layers were dried over $MgSO_4$, filtered and concentrated under reduced pressure. Methyl ester **S11** (2.58 g, 5.50 mmol, quantitative) was obtained as a colourless solid.

R_f = 0.60 (CH_2Cl_2/CH_3OH 98:2); mp = 123 – 124 °C; $[\alpha]_D^{20}$ = +5.8 (*c* = 1.04, DMF); ^{1}H-NMR (400 MHz, $CDCl_3$, $CHCl_3$ = 7.26 ppm): δ = 7.77 (2H, d, *J* = 7.5 Hz, H-17, H-23), 7.61 (2H, m, H-14, H-20), 7.40 (2H, m, H-16, H-22), 7.32 (2H, m, H-15, H-21), 5.47 (1H, brm, NH-a), 4.59 (1H, brm, NH-b), 4.40 (3H, m, H-2, H-10), 4.22 (1H, t, *J* = 7.0 Hz, H-11), 3.76 (3H, s, H-24), 3.15 (2H, brm, H-5), 1.88 (1H, m, H-3), 1.68 (1H, m, H-3‘), 1.60-1.48 (2H, m, H-4), 1.45 (9H, s, H-8) ppm; ^{13}C-NMR (100 MHz, $CDCl_3$, $CDCl_3$ = 77.16 ppm): δ = 172.9 (q, C-1), 156.11 (q, C-6), 156.05 (q, C-9), 144.0 (q, C-12 o. C-18), 143.9 (q, C-12 o. C-18), 141.5 (q, C-13 o. C-19), 141.4 (q, C-13 o. C-19), 129.2 (t, C-15 o. C-16 o. C-21 o. C-22), 128.4 (t, C-15 o. C-16 o. C-21 o. C-22), 127.8 (t, C-15 o. C-16 o. C-21 o. C-22), 127.2 (t, C-15 o. C-16 o. C-21 o. C-22), 125.4 (t, C-14 o. C-20), 125.2 (t, C-14 o. C-20), 120.11 (t, C-17 o. C-23), 120.09 (t, C-17 o. C-23), 79.4 (q, C-7), 67.1 (s, C-10), 53.7 (t, C-2), 52.6 (p, C-24), 47.3 (t, C-11), 40.1 (s, C-5), 30.0 (s, C-3), 28.5 (3C, p, C-8), 26.2 (s, C-4) ppm; HRMS (ESI): *m/z* calculated for $C_{26}H_{32}N_2O_6Na$ $[M + Na]^+$: 491.2158; found 491.2160.

Synthesis of methyl (*R*)-2-amino-5-[(*tert*-butoxycarbonyl)amino]pentanoate (23)

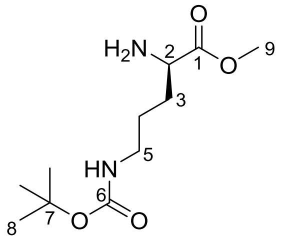

Fmoc-D-Orn(Boc)-OMe (**S11**; 1.00 g, 2.13 mmol, 1.00 equiv) was dissolved in CH_2Cl_2 (150 mL) at 0 °C. Then, tris(2-aminoethyl)amine (15.0 mL) was added dropwise. The reaction mixture was warmed to rt and stirred for 3 h. Afterwards, H_2O was added. The layers were separated and the organic layer was washed with H_2O (2×). The combined organic layers were dried over $MgSO_4$, filtered and concentrated under reduced pressure. Purification by flash column chromatography over silica (100% CH_2Cl_2 → CH_2Cl_2/CH_3OH = 99:1 → CH_2Cl_2/CH_3OH = 98:2 → CH_2Cl_2/CH_3OH = 96:4) gave the title compound **23** (484 mg, 1.96 mmol, 92%) as a colourless oil.

R_f = 0.24 (CH_2Cl_2/CH_3OH 98:2); $[\alpha]_D^{20}$ = -17.9 (*c* = 1.83, CH_3OH); ^{1}H-NMR (400 MHz, CD_3OD, CD_3OH = 3.31 ppm): δ = 3.72 (3H, s, H-9), 3.45 (1H, t, *J* = 6.3 Hz, H-2), 3.05 (2H, t, *J* = 6.7 Hz, H-5), 1.72 (1H, m, H-3), 1.61 (1H, m, H-3‘), 1.56-1.49 (2H, m, H-4), 1.43 (9H, s, H-8) ppm; ^{13}C-NMR (100 MHz, CD_3OD, CD_3OD = 49.00 ppm): δ = 177.0 (q, C-1), 158.4 (q, C-6), 79.8 (q, C-7), 54.8 (t, C-2), 52.4 (p, C-9), 41.0 (s, C-5), 32.8 (s, C-3), 28.8 (3C, p, C-8), 27.0 (s, C-4) ppm; HRMS (ESI): *m/z* calculated for $C_{11}H_{22}N_2O_4Na$ $[M + Na]^+$: 269.1477; found 269.1476.

Synthesis of methyl (*R*)-5-[(*tert*-butoxycarbonyl)amino]-2-(2-iodo-3-methylbut-2-enamido)pentanoate (29)

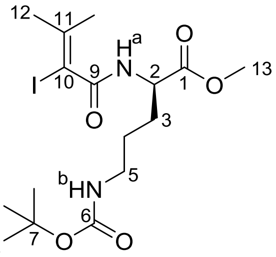

Acid **10** (371 mg, 1.64 mmol, 1.00 equiv) and amine **23** (445 mg, 1.81 mmol, 1.10 equiv) were dissolved in DMF (9.80 mL) at 0 °C. Then, HOAt (239 mg, 1.76 mmol, 1.07 equiv) and PyAOP (897 mg, 1.72 mmol, 1.05 equiv) were added. Afterwards, DIPEA (974 mg, 7.53 mmol, 4.60 equiv) was added dropwise. The reaction mixture was warmed to rt. After 22 h of stirring, the reaction was terminated by addition of a saturated NH_4Cl solution. The layers were separated and the aqueous layer was extracted with EtOAc (7×). The combined organic layers were washed with brine and subsequently dried over Na_2SO_4, filtered and concentrated under reduced pressure. Purification by flash column chromatography over

silica (petroleum ether/ ethyl acetate = 4:1 → petroleum ether/ ethyl acetate = 3:1 → petroleum ether/ ethyl acetate = 1:1) gave the title compound **29** (641 mg, 1.41 mmol, 86%) as a colourless solid.

R_f = 0.83 (CH_2Cl_2/CH_3OH 98:2); mp = 112 – 113 °C; $[\alpha]_D^{20}$ = +3.5 (*c* = 1.04, CH_2Cl_2); ^{1}H-NMR (400 MHz, $CDCl_3$, $CHCl_3$ = 7.26 ppm): δ = 6.38 (1H, d, *J* = 6.1 Hz, NH-a), 4.61 (2H, m, H-2, NH-b), 3.77 (3H, s, H-13), 3.15 (2H, m, H-5), 2.05 (3H, s, H-12), 2.01 (3H, s, H-12), 1.94 (1H, m, H-3), 1.73 (1H, m, H-3‘), 1.57 (2H, m, H-4), 1.43 (9H, s, H-8) ppm; ^{13}C-NMR (100 MHz, $CDCl_3$, $CDCl_3$ = 77.16 ppm): δ = 172.6 (q, C-1), 167.1 (q, C-9), 156.3 (q, C-6), 146.2 (q, C-11), 86.3 (q, C-10), 79.4 (q, C-7), 52.7 (t, C-2), 52.4 (p, C-13), 40.1 (s, C-5), 30.2 (p, C-12), 29.7 (s, C-3), 28.5 (3C, p, C-8), 26.3 (s, C-4), 22.1 (p, C-12) ppm; HRMS (ESI): *m/z* calculated for $C_{16}H_{27}IN_2O_5Na$ $[M + Na]^+$: 477.0862; found 477.0858.

Synthesis of methyl [(*R*)-5-[(*tert*-butoxycarbonyl)amino]-2-(2-iodo-3-methylbut-2-enamido)pentanoyl]-D-valinate (35)

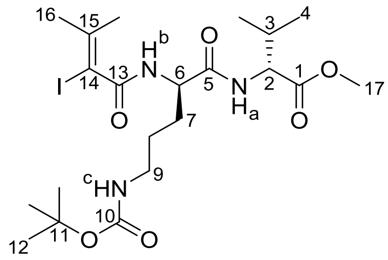

Dipeptide **29** (262 mg, 0.58 mmol, 1.00 equiv) was dissolved in THF (3.20 mL) at 0 °C. Then, LiOH (1.0 M; 3.20 mL) was added. The reaction mixture was warmed up to rt and stirred for 16 h. The reaction mixture was acidified with HCl (1.0 M) to pH ~ 2 and extracted with EtOAc (6×). The combined organic layers were washed with brine and then dried over $MgSO_4$, filtered and concentrated under reduced pressure. The product was used without further purification.

The resulting crude product (254 mg, 0.58 mmol, 1.00 equiv) and D-valine methylester hydrochloride (126 mg, 0.75 mmol, 1.30 equiv) were dissolved in DMF (3.60 mL) at rt. The reaction mixture was cooled to 0 °C. Then, HOAt (84.1 mg, 0.62 mmol, 1.05 equiv) and PyAOP (316 mg, 0.61 mmol, 1.07 equiv) were added. Afterwards, DIPEA (343 mg, 2.65 mmol, 4.60 equiv) was added dropwise. The reaction mixture was stirred at 0 °C → rt. After 23 h, a saturated NH_4Cl solution was added. The layers were separated and the aqueous layer was extracted with EtOAc (8×). The combined organic layers were washed with brine and subsequently dried over $MgSO_4$, filtered and concentrated under reduced pressure. Purification by flash column chromatography over silica (petroleum ether/ ethyl acetate = 3:1 → petroleum ether/ethyl acetate = 2:1) yielded the title compound **35** (279 mg, 0.50 mmol, 87% over two steps) as a colourless solid.

R_f = 0.67 (CH_2Cl_2/CH_3OH 98:2); mp = 123 – 126 °C; $[\alpha]_D^{20}$ = +7.2 (*c* = 1.39, CH_2Cl_2); ^{1}H-NMR (400 MHz, $CDCl_3$, $CHCl_3$ = 7.26 ppm): δ = 6.96 (1H, brm, NH-a), 6.64 (1H, brm, NH-b), 4.83 (1H, brm, NH-c), 4.64 (1H, m, H-6), 4.46 (1H, dd, *J* = 5.3, 8.4 Hz, H-2), 3.70 (3H, s, H-17), 3.27 (1H, m, H-9), 3.09 (1H, m, H-9'), 2.16 (1H, m, H-3), 1.99 (3H, s, H-16), 1.96 (3H, s, H-16), 1.92 (1H, m, H-7), 1.67 (1H, m, H-7'), 1.56 (2H, m, H-8), 1.40 (9H, s, H-12), 0.93-0.90 (6H, m, H-4) ppm; ^{13}C-NMR (100 MHz, $CDCl_3$, $CDCl_3$ = 77.16 ppm): δ = 172.1 (q, C-1), 171.6 (q, C-5), 167.5 (q, C-13), 156.6 (q, C-10), 145.6 (q, C-15), 86.6 (q, C-14), 79.3 (q, C-11), 57.6 (t, C-2), 52.7 (t, C-6), 52.2 (p, C-17), 39.6 (s, C-9), 31.4 (t, C-3), 30.9 (p, C-16), 30.0 (s, C-7), 28.5 (3C, p, C-12), 26.4 (s, C-8), 22.0 (p, C-16), 19.2 (p, C-4), 18.0 (p, C-4) ppm; HRMS (ESI): *m/z* calculated for $C_{21}H_{36}IN_3O_6Na$ $[M + Na]^+$: 576.1547; found 576.1548.

Synthesis of methyl (*R*)-5-{[(benzyloxy)carbonyl]amino}-2-(2-iodo-3-methylbut-2-enamido)pentanoate (28)

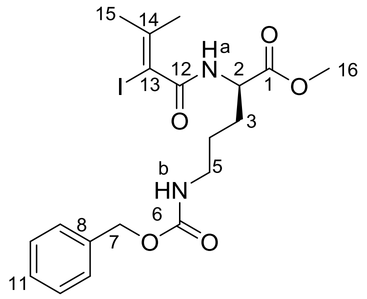

Boc-D-Orn(Cbz)-OH (400 mg, 1.09 mmol, 1.00 equiv) was dissolved in CH_3OH (2.30 mL) at 0 °C. Then, freshly distilled thionylchloride (773 mg, 6.50 mmol, 5.95 equiv) was added dropwise. The reaction mixture was stirred at 0 °C → rt. After 18 h, the solvent was removed under reduced pressure. The resulting residue was charged with Et_2O and the solvent was removed under reduced pressure. This procedure was repeated several times. The crude product **22** was used without further purification.

Acid **10** (138 mg, 0.61 mmol, 1.00 equiv) und amine **22** (222 mg, 0.79 mmol, 1.30 equiv) were dissolved in DMF (3.30 mL) at 0 °C. Then, HOAt (88.7 mg, 0.65 mmol, 1.07 equiv) and PyAOP (333 mg, 0.64 mmol, 1.05 equiv) were added. Afterwards, DIPEA (361 mg, 2.79 mmol, 4.59 equiv) was added dropwise. The reaction mixture was stirred at 0 °C → rt. After 22 h, the reaction was terminated by adding a saturated NH_4Cl solution. The layers were separated and the aqueous layer was extracted with EtOAc (7×). The combined organic layers were washed with brine and subsequently dried over $MgSO_4$ as well as filtered. The solvent was removed under reduced pressure. Purification by flash chromatography over silica (petroleum ether/ethyl acetate = 3:1 → petroleum ether/ethyl acetate = 1:1) yielded the desired vinyl iodide **28** (197 mg, 0.40 mmol, 66% over two steps) as a colourless solid.

R_f = 0.65 (CH_2Cl_2/CH_3OH 98:2); mp = 122 – 124 °C; $[\alpha]_D^{20}$ = +3.2 (*c* = 1.11, CH_2Cl_2); ^{1}H-NMR (400 MHz, $CDCl_3$, $CHCl_3$ = 7.26 ppm): δ = 7.37-7.28 (5H, m, ArH), 6.39 (1H, d, *J* = 7.5 Hz, NH-a), 5.07 (2H, s, H-7), 4.98 (1H, brm, NH-b), 4.60 (1H, m, H-2), 3.74 (3H, s, H-16), 3.22 (2H, q, *J* = 6.4 Hz, H-5), 2.02 (3H, s, H-15), 1.99 (3H, s, H-15), 1.94 (1H, m, H-3), 1.76 (1H, m, H-3‘), 1.55 (2H, m, H-4) ppm; ^{13}C-NMR (100 MHz, $CDCl_3$, $CDCl_3$ = 77.16 ppm): δ = 172.4 (q, C-1), 167.1 (q, C-12), 156.6 (q, C-6), 146.2 (q, C-14), 136.6 (q, C-8), 128.6 (3C, t, C-10, C-11), 128.2 (2C, t, C-9), 86.4 (q, C-13), 66.8 (s, C-7), 52.7 (p, C-16), 52.3 (t, C-2), 40.5 (s, C-5), 30.1 (p, C-15), 29.6 (s, C-3), 26.1 (s, C-4), 22.1 (p, C-15) ppm; HRMS (ESI): *m/z* calculated for $C_{19}H_{25}IN_2O_5Na$ $[M + Na]^+$: 511.0706; found 511.0704.

Synthesis of *tert*-butyl (*S*)-1-(1-amino-3-methyl-1-oxobutan-2-yl)carbamate (30)

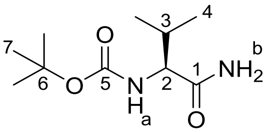

Boc-L-valine (4.00 g, 18.4 mmol, 1.00 equiv) and *N*-methylmorpholine (2.01 g, 19.9 mmol, 1.08 equiv) were dissolved in 1,2-dimethoxyethane (94.0 mL) at 0 °C. Then, isobutylchloroformiate (2.72 g, 19.9 mmol, 1.08 equiv) was added dropwise. The reaction mixture was stirred for 15 min at 0 °C. Afterwards, an aqueous ammonia solution (25%; 8.20 mL, 121 mmol, 6.57 equiv) was added dropwise. The temperature was raised to rt while stirring. After 22.5 h, the reaction was terminated by adding HCl (1.0 M). The aqueous layer was extracted with EtOAc (4×). The combined organic layers were washed with HCl (0.1 M) and subsequently dried over $MgSO_4$, filtered and concentrated under reduced pressure. Amide **30** (3.91 g, 18.1 mmol, 98%) was obtained as a colourless solid.

The spectroscopic data matches those reported in the literature[S6].

R_f = 0.20 (CH_2Cl_2/CH_3OH = 98:2); mp = 155 – 157 °C (mp = 155 – 157 °C, ref.[S7]); $[\alpha]_D^{20}$ = -2.4 (*c* 1.16, CH_3OH) {$[\alpha]_D^{20}$ = -0.4 (*c* 1.46, CH_3OH) ref.[S6]}; ^{1}H-NMR (400 MHz, $CDCl_3$, $CHCl_3$ = 7.26 ppm): δ = 6.08 (1H, brs, NH-b), 5.67 (1H, brs, NH-b), 5.08 (1H, brm, NH-a), 3.97 (1H, t, *J* = 7.0 Hz, H-2), 2.14 (1H, m, H-3), 1.44 (9H, s, H-7), 0.99 (3H, d, *J* = 6.8 Hz, H-4), 0.94 (3H, d, *J* = 6.8 Hz, H-4) ppm; ^{13}C-NMR (100 MHz, $CDCl_3$, $CDCl_3$ = 77.16 ppm): δ = 174.2 (q, C-1), 156.1 (q, C-5), 80.2 (q, C-6), 59.6 (t, C-2), 30.8 (t, C-3), 28.5 (3C, p, C-7), 19.4 (2C, p, C-4) ppm; HRMS (ESI): *m/z* calculated for $C_{10}H_{20}N_2O_3Na$ $[M + Na]^+$: 239.1372; found 239.1372.

Synthesis of methyl (*E*)-*N*$^{\omega}$,*N*$^{\omega'}$-bis[(allyloxyl)carbonyl]-*N*^{2}-{2-[(*S*)-2-[(*tert*-butoxy-carbonyl)amino]-3-methylbutanamido]-3-methylbut-2-enoyl}-D-arginyl-D-valinate (31)

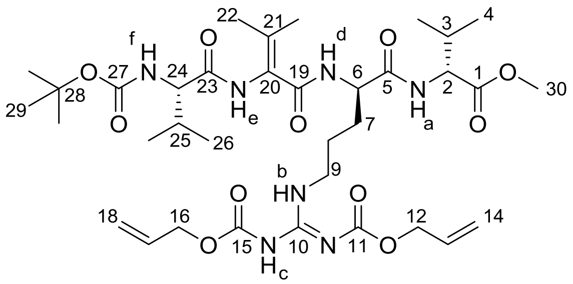

Boc-L-Val-NH_2 (**30**; 32.6 mg, 0.15 mmol, 1.00 equiv) and vinyl iodide **25** (205 mg, 0.31 mmol, 2.00 equiv) were dissolved in 1,4-dioxane (degassed; 0.15 mL) at rt. Then, K_2CO_3 (41.7 mg, 0.31 mmol, 2.0 equiv), CuI (17.2 mg, 90.0 µmol, 0.60 equiv) and *trans*-*N*,*N*-dimethyl-1,2-cyclohexanediamine (86.8 mg, 0.61 mmol, 4.05 equiv) were added. The reaction mixture was heated to 70 °C. After 22 h, the solvent was removed under reduced pressure. The resulting residue was dissolved in CH_3OH (1.8 mL) and purified by preparative HPLC (C18) (gradient CH_3OH/H_2O = 20:80 {5 min} → CH_3OH/H_2O = 50:50 {25 min} → CH_3OH/H_2O = 50:50 {15 min} → CH_3OH/H_2O = 100:0 {40 min} → CH_3OH/H_2O = 100:0 {20 min} 15 mL/min.) (t_R = 69.5 min.). The desired tetrapeptide **31** (13.6 mg, 18.1 µmol, 13%) was obtained as a colourless oil.

^{1}H-NMR (400 MHz, $CDCl_3$, $CHCl_3$ = 7.26 ppm): δ = 11.71 (1H, brs, NH-c), 8.34 (1H, t, *J* = 5.1 Hz, NH-b), 7.34 (1H, s, NH-e), 7.05 (1H, m, NH-a o. NH-d), 6.97 (1H, d, *J* = 8.5 Hz, NH-a o. NH-d), 5.94 (2H, m, H-13, H-17), 5.38-5.20 (4H, m, H-14, H-18), 5.02 (1H, m, NH-f), 4.64-4.58 (4H, m, H-12, H-16), 4.45 (2H, m, H-2, H-6), 3.87 (1H, t, *J* = 5.6 Hz, H-24), 3.71 (3H, s, H-30), 3.47 (2H, m, H-9), 2.16 (2H, m, H-3, H-25), 2.09 (3H, s, H-22), 2.01 (1H, m, H-7), 1.78 (3H, s, H-22), 1.71 (3H, m, H-7‘, H-8), 1.43 (9H, s, H-29), 1.02 (6H, d, *J* = 6.8 Hz, H-4 o. H-26), 0.98 (3H, d, *J* = 6.8 Hz, H-4 o. H-26), 0.90 (3H, d, *J* = 6.8 Hz, H-4 o. H-26) ppm; HRMS (ESI): *m/z* calculated for $C_{35}H_{57}N_7O_{11}Na$ $[M + Na]^+$: 774.4014; found 774.4015.

Synthesis of methyl [(*R*)-5-{[(benzyloxy)carbonyl]amino}-2-{2-[(*S*)-2[(*tert*-butoxy-carbonyl)amino]-3-methylbutanamido]-3-methylbut-2-enamido}pentanoyl]-D-valinate (32)

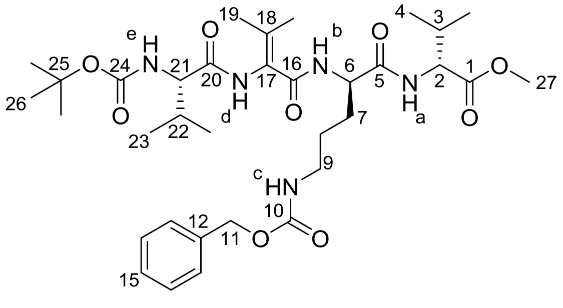

Boc-L-Val-NH_2 (**30**; 14.5 mg, 67.0 µmol, 1.00 equiv) and vinyl iodide **24** (78.8 mg, 0.13 mmol, 2.00 equiv) were dissolved in 1,4-dioxane (degassed; 0.13 mL) at rt. Then, K_2CO_3 (18.5 mg, 0.13 mmol, 2.00 equiv), CuI (7.66 mg, 40.2 µmol, 0.60 equiv) and *trans*-*N*,*N*-dimethyl-1,2-cyclohexanediamine (38.6 mg, 0.27 mmol, 4.05 equiv) were added. The reaction mixture was heated to 70 °C. After 22 h, the solvent was removed under reduced pressure. The resulting residue was charged with a saturated NH_4Cl solution. Then, the aqueous layer was extracted with EtOAc (4×). The combined organic layers were washed with brine and subsequently dried over $MgSO_4$ as well as filtered. The solvent was removed under reduced pressure. Purification by flash chromatography on silica (100% CH_2Cl_2 → CH_2Cl_2/CH_3OH 99:1 → CH_2Cl_2/CH_3OH 98:2) yielded the desired product **32** (22.5 mg, 33.3 µmol, 50%) as a colourless solid.

R_f = 0.21 (CH_2Cl_2/CH_3OH 98:2); $[\alpha]_D^{20}$ = +21.3 (*c* = 0.97, CH_2Cl_2); ^{1}H-NMR (400 MHz, $CDCl_3$, $CHCl_3$ = 7.26 ppm): δ = 7.80 (1H, s, NH-d), 7.37-7.28 (5H, m, ArH), 7.17 (2H, m, NH-a, NH-b), 5.32 (1H, t, *J* = 4.1 Hz, NH-c), 5.20 (1H, d, *J* = 6.2 Hz, NH-e), 5.07 (2H, s, H-11), 4.60 (1H, m, H-6), 4.45 (1H, dd, *J* = 6.0, 8.0 Hz, H-2), 3.90 (1H, m, H-21), 3.70 (3H, s, H-27), 3.27 (1H, m, H-9), 3.15 (1H, m, H-9‘), 2.14 (2H, m, H-3, H-22), 2.06 (3H, s, H-19), 1.93 (1H, m, H-7), 1.76 (3H, s, H-19), 1.70-1.53 (3H, m, H-7‘, H-8), 1.42 (9H, s, H-26), 0.98 (6H, d, *J* = 6.8 Hz, H-4), 0.94-0.90 (6H, m, H-23) ppm; ^{13}C-NMR (100 MHz, $CDCl_3$, $CDCl_3$ = 77.16 ppm): δ = 172.3 (q, C-1), 172.0 (q, C-5 o. C-20), 171.6 (q, C-5 o. C-20), 166.4 (q, C-16), 157.1 (q, C-10), 156.6 (q, C-24), 140.7 (q, C-17), 136.7 (q, C-12), 128.6 (3C, t, C-14, C-15), 128.2 (2C, t, C-13), 123.5 (q, C-18), 80.7 (q, C-25), 66.8 (s, C-11), 60.8 (t, C-21), 57.5 (t, C-2), 52.5 (t, C-6), 52.2 (p, C-27), 40.2 (s, C-9), 31.0 (t, C-3 o. C-22), 30.3 (t, C-3 o. C-22), 28.6 (s, C-7), 28.4 (3C, p, C-26), 25.9 (s, C-8), 21.5 (p, C-19), 21.0 (p, C-19), 19.6 (p, C-4), 19.1 (p, C-4), 18.2 (p, C-23), 18.0 (p, C-23) ppm; HRMS (ESI): *m/z* calculated for $C_{34}H_{53}N_5O_9Na$ $[M + Na]^+$: 698.3741; found 698.3740.

Synthesis of methyl (6*S*,12*R*)-12-(3-{[(benzyloxy)carbonyl]amino}propyl)-6-isopropyl-2,2-dimethyl-4,7,10-trioxo-9-(propan-2-ylidene)-3-oxa-5,8,11-triazatridecan-13-oate (33)

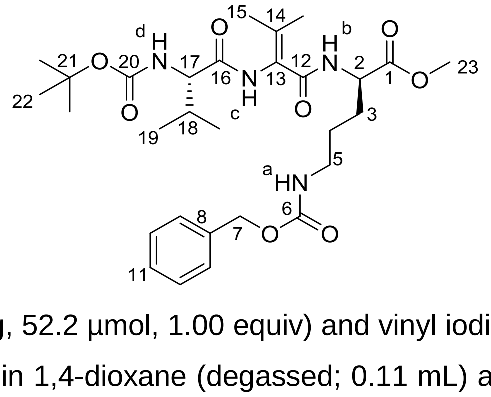

Boc-L-Val-NH_2 (**30**; 11.3 mg, 52.2 µmol, 1.00 equiv) and vinyl iodide **28** (50.8 mg, 0.10 mmol, 2.00 equiv) were dissolved in 1,4-dioxane (degassed; 0.11 mL) at rt. Then, K_2CO_3 (14.4 mg, 0.10 mmol, 2.00 equiv), CuI (5.94 mg, 31.1 µmol, 0.60 equiv) and *trans*-*N*,*N*-dimethyl-1,2-

cyclohexanediamine (30.0 mg, 0.21 mmol, 4.05 equiv) were added. The reaction mixture was heated to 70 °C. After 22 h, the solvent was removed under reduced pressure. The resulting residue was charged with a saturated NH_4Cl solution. Then, the aqueous layer was extracted with EtOAc (4×). The combined organic layers were washed with brine and subsequently dried over $MgSO_4$ as well as filtered. The solvent was removed under reduced pressure. Purification by flash chromatography on silica (100% CH_2Cl_2 → CH_2Cl_2/CH_3OH 99:1 → CH_2Cl_2/CH_3OH 98:2) yielded the desired product **33** (15.1 mg, 26.2 µmol, 50%) as a colourless solid.

R_f = 0.21 (CH_2Cl_2/CH_3OH 98:2); ^{1}H-NMR (400 MHz, $CDCl_3$, $CHCl_3$ = 7.26 ppm): δ = 7.71 (1H, s, NH-c), 7.36-7.28 (5H, m, ArH), 7.04 (2H, d, *J* = 7.5 Hz, NH-b), 5.45 (1H, t, *J* = 5.0 Hz, NH-a), 5.17 (1H, m, NH-d), 5.08 (2H, s, H-7), 4.61 (1H, m, H-2), 3.89 (1H, t, *J* = 4.4 Hz, H-17), 3.70 (3H, s, H-23), 3.19 (2H, m, H-5), 2.11 (1H, m, H-18), 2.06 (3H, s, H-15), 1.96 (1H, m, H-3), 1.85 (1H, m, H-3‘), 1.75 (3H, s, H-15), 1.68-1.53 (2H, m, H-4), 1.44 (9H, s, H-22), 0.95-0.93 (6H, m, H-19) ppm; HRMS (ESI): *m/z* calculated for $C_{29}H_{44}N_4O_8Na$ $[M + Na]^+$: 599.3057; found 599.3503.

Synthesis of compound 34 through intermediate S12

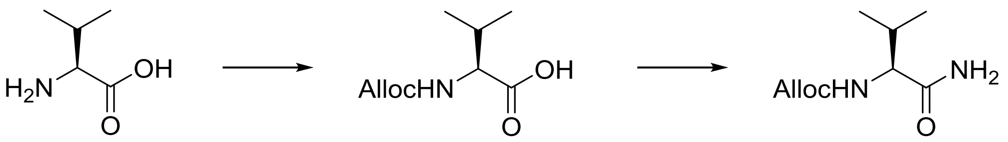

S12 **34**

Synthesis of [(allyloxy)carbonyl]-L-valine (S12)

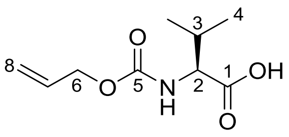

L-Valine (2.00 g, 17.1 mmol, 1.00 equiv) and K_2CO_3 (3.54 g, 25.6 mmol, 1.50 equiv) were dissolved in THF/H_2O (1:1; 80.0 mL) at 0 °C. Afterwards, allylchloroformiate (2.47 g, 20.5 mmol, 1.20 equiv) was added dropwise. The reaction mixture was strirred for 16 h at 0 °C → rt. Then, the solvent was removed under reduced pressure. The resulting residue was extracted with Et_2O (3x). The aqueous layer was acidified with HCl (conc.) towards pH ~ 2 and subsequently extracted with CH_2Cl_2 (5×). The combined organic layers were washed with brine and dried over $MgSO_4$. After filtration, the solvent was removed under reduced pressure. The desired product **S12** (3.44 g, 17.1 mmol, quantitative) was obtained as a colourless oil.

The spectroscopic data matches those reported in the literature.[S8]

$[\alpha]_D^{20}$ = -2.9 (*c* = 2.48, DMSO) {ref.[S9]: $[\alpha]_D^{20}$ = -10 (*c* = 1.00, EtOH)}; ^{1}H-NMR (400 MHz, DMSO-d$_6$, DMSO-d$_5$ = 2.50 ppm): δ = 12.55 (1H, brs, OH), 7.41 (1H, d, *J* = 8.5 Hz, NH), 5.90 (1H, m, H-7), 5.30 (1H, dd, *J* = 1.7, 17.4 Hz, H-8), 5.17 (1H, dd, *J* = 1.7, 10.6 Hz, H-8), 4.48 (2H, dt, *J* = 0.9, 5.1 Hz, H-6), 3.85 (1H, dd, *J* = 6.4, 8.5 Hz, H-2), 2.04 (1H, m, H-3), 0.88 (6H, t, *J* = 6.4 Hz, H-4) ppm; ^{13}C-NMR (100 MHz, DMSO-d$_6$, DMSO-d$_6$ = 39.52 ppm): δ = 173.3 (q, C-1), 156.2 (q, C-5), 133.6 (t, C-7), 117.0 (s, C-8), 64.5 (s, C-6), 59.5 (t, C-2), 29.5 (t, C-3), 19.2 (p, C-4), 18.0 (p, C-4) ppm; HRMS (ESI): *m/z* calculated for $C_9H_{15}NO_4Na$ [M + Na]$^+$: 224.0899; found 224.0900.

Synthesis of allyl (*S*)-1-(1-amino-3-methyl-1-oxobutan-2-yl)carbamate (34)

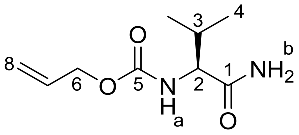

Alloc-L-valine (**S12**; 3.44 g, 17.1 mmol, 1.00 equiv) and *N*-methylmorpholine (1.87 g, 18.4 mmol, 1.08 equiv) were dissolved in 1,2-dimethoxyethane (87.0 mL) at 0 °C. Then, isobutylchloroformiate (2.52 g, 18.4 mmol, 1.08 equiv) was added dropwise. The reaction mixture was stirred for 15 min at 0 °C. Afterwards, an aqueous ammonia solution (25%; 7.60 mL, 112 mmol, 6.57 equiv) was added dropwise. The temperature was raised to rt while stirring. After 23 h, the reaction was terminated by adding HCl (1.0 M). The aqueous layer was extracted with EtOAc (6×). The combined organic layers were washed with HCl (0.1 M) and subsequently dried over $MgSO_4$, filtered and the solvent was removed under reduced pressure. Amide **34** (3.00 g, 15.0 mmol, 88%) was obtained as a colourless solid.

R_f = 0.18 (CH_2Cl_2/CH_3OH 98:2); mp = 147 – 149 °C; $[\alpha]_D^{20}$ = -5.9 (*c* = 1.39, CH_3OH); ^{1}H-NMR (400 MHz, DMSO-d$_6$, DMSO-d$_5$ = 2.50 ppm): δ = 7.32 (1H, brs, NH-b), 7.06 (1H, d, *J* = 9.0 Hz, NH-a), 7.01 (1H, brs, NH-b), 5.90 (1H, m, H-7), 5.29 (1H, ddd, *J* = 1.4, 3.1, 17.1 Hz, H-8), 5.17 (1H, ddd, *J* = 1.4, 1.4, 10.6 Hz, H-8), 4.47 (2H, dt, *J* = 1.4, 5.1 Hz, H-6), 3.78 (1H, dd, *J* = 6.8, 9.0 Hz, H-2), 1.94 (1H, m, H-3), 0.86 (3H, d, *J* = 6.8 Hz, H-4), 0.83 (3H, d, *J* = 6.8 Hz, H-4) ppm; ^{13}C-NMR (100 MHz, DMSO-d$_6$, DMSO-d$_6$ = 39.52 ppm): δ = 173.2 (q, C-1), 156.0 (q, C-5), 133.7 (t, C-7), 116.9 (s, C-8), 64.4 (s, C-6), 59.9 (t, C-2), 30.1 (t, C-3), 19.3 (p, C-4), 18.0 (p, C-4) ppm; HRMS (ESI): *m/z* calculated for $C_9H_{16}N_2O_3Na$ [M + Na]$^+$: 223.1059; found 223.1059.

Synthesis of methyl (2*R*,8*S*)-2-{3-[(*tert*-butoxycarbonyl)amino]propyl}-8-isopropyl-4,7,10-trioxo-5-(propan-2-ylidene)-11-oxa-3,6,9-triazatetradec-13-enoate (36)

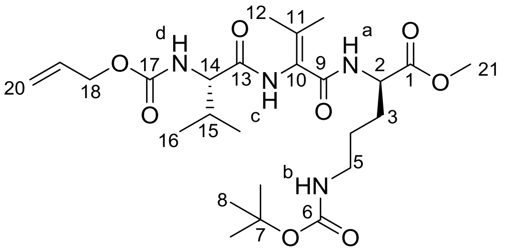

Alloc-L-Val-NH$_2$ (**34**; 35.3 mg, 0.18 mmol, 1.00 equiv) and vinyl iodide **29** (160 mg, 0.35 mmol, 2.00 equiv) were dissolved in 1,4-dioxane (degassed; 0.18 mL) at rt. Then, K_2CO_3 (48.7 mg, 0.35 mmol, 2.00 equiv), CuI (20.1 mg, 0.11 mmol, 0.60 equiv) and *trans*-*N*,*N*-dimethyl-1,2-cyclohexanediamine (101 mg, 0.71 mmol, 4.05 equiv) were added. The reaction mixture was heated to 70 °C. After 22 h, the reaction was terminated by adding a saturated NH_4Cl solution. Then, the aqueous layer was extracted with EtOAc (6×). The combined organic layers were washed with brine and subsequently dried over $MgSO_4$ as well as filtered. The solvent was removed under reduced pressure. The resulting residue was dissolved in CH_3OH (1.8 mL) and purified by preparative HPLC (C18) (gradient CH_3OH/H_2O 10:90 {5 min} → CH_3OH/H_2O = 100:0 {85 min} → CH_3OH/H_2O = 100:0 {10 min}, 15.0 mL/min) (t_R = 70.8 min). Dehydropeptide **36** (10.4 mg, 19.7 µmol, 11%) was obtained as a colourless oil.

^{1}H-NMR (400 MHz, $CDCl_3$, $CHCl_3$ = 7.26 ppm): δ = 7.64 (1H, brs, NH-c), 7.01 (1H, brd, NH-a), 5.90 (1H, m, H-19), 5.43 (1H, brs, NH-d), 5.35-5.27 (2H, m, H-20), 4.88 (1H, brm, NH-b), 4.56 (2H, m, H-18), 4.46 (1H, m, H-2), 3.94 (1H, t, *J* = 6.8 Hz, H-14), 3.71 (3H, s, H-21), 3.12 (2H, m, H-5), 2.17 (1H, m, H-15), 2.10 (3H, s, H-12), 1.91 (1H, m, H-3), 1.81 (3H, s, H-12), 1.79 (1H, m, H-3'), 1.53 (2H, m, H-4), 1.42 (9H, s, H-8), 0.92 (6H, m, H-16) ppm; HRMS (ESI): *m/z* calculated for $C_{25}H_{42}N_4O_8Na$ $[M + Na]^+$: 549.2900; found 549.2899.

Synthesis of methyl [(*R*)-2-{2-[(*S*)-2-{[(allyloxy)carbonyl]amino}-3-methylbutanamido]-3-methylbut-2-enamido}-5-[(*tert*-butoxycarbonyl)amino]pentanoyl]-D-valinate (37)

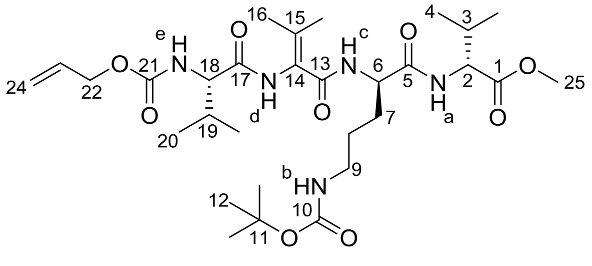

Alloc-L-Val-NH$_2$ (**34**; 28.0 mg, 0.14 mmol, 1.00 equiv) and vinyl iodide **35** (155 mg, 0.28 mmol, 2.00 equiv) were dissolved in 1,4-dioxane (degassed; 0.15 mL) at rt. Then, K_2CO_3 (38.7 mg, 0.28 mmol, 2.00 equiv), CuI (16.0 mg, 84.1 µmol, 0.60 equiv) and *trans*-*N*,*N*-dimethyl-1,2-cyclohexanediamine (80.6 mg, 0.57 mmol, 4.05 equiv) were added. The

reaction mixture was heated to 70 °C. After 22 h, the reaction was terminated by adding a saturated NH_4Cl solution. Then, the aqueous layer was extracted with EtOAc (6×). The combined organic layers were washed with brine and subsequently dried over $MgSO_4$ as well as filtered. The solvent was removed under reduced pressure. The resulting residue was dissolved in CH_3OH (1.8 mL) and purified by preparative HPLC (C18) (gradient CH_3OH/H_2O 10:90 {5 min} → CH_3OH/H_2O = 100:0 {85 min} → CH_3OH/H_2O = 100:0 {10 min}, 15.0 mL/min) (t_R = 73.8 min). Dehydropeptide **37** (11.5 mg, 18.4 µmol, 13%) was obtained as a colourless oil.

^{1}H-NMR (400 MHz, $CDCl_3$, $CHCl_3$ = 7.26 ppm): δ = 7.64 (1H, brs, NH-e), 7.06 (1H, brd, NH-c), 7.01 (1H, brd, NH-a), 5.90 (1H, m, H-23), 5.43 (1H, brs, NH-d), 5.31-5.22 (2H, m, H-24), 4.88 (1H, brm, NH-b), 4.61-4.50 (2H, m, H-22), 4.44-4.39 (2H, m, H-2, H-6), 3.95 (1H, t, *J* = 6.8 Hz, H-18), 3.71 (3H, s, H-25), 3.12 (2H, m, H-9), 2.17 (2H, m, H-3, H-19), 2.10 (3H, s, H-16), 1.92 (1H, m, H-7), 1.81 (3H, s, H-16), 1.79 (1H, m, H-7'), 1.55-1.50 (2H, m, H-8), 1.42 (9H, s, H-12), 0.98-0.92 (12H, m, H-4, H-20) ppm; HRMS (ESI): *m/z* calculated for $C_{30}H_{51}N_5O_9Na$ $[M + Na]^+$: 648.3584; found 648.3588.

Synthesis of compound 38 through intermediates S13-S15

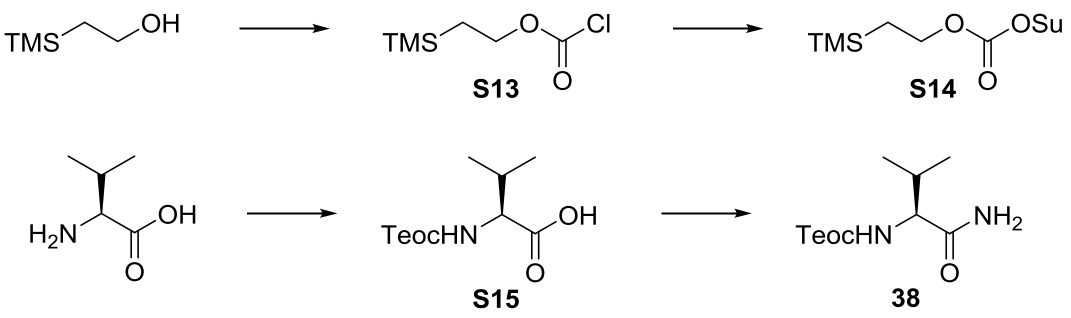

Synthesis of (2-(trimethylsilyl)ethoxycarbonyloxy)chloride (S13)

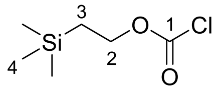

2-Trimethylsilylethanol (4.13 g, 34.9 mmol, 1.00 equiv) and K_2CO_3 (4.15 g, 30.0 mmol, 0.86 equiv) were dissolved in toluene (20.0 mL) at −10 °C. Afterwards, phosgene (20% in toluene; 24.0 mL, 45.4 mmol, 1.30 equiv) was added dropwise. The reaction mixture was stirred for 30 min at −10 °C, then warmed to rt and stirred for additional 60 min. Excess of phosgene was removed by passing a stream of nitrogen gas through the flask. Then, the flask was neutralized by washing it with a saturated K_2CO_3 solution. Then, the solvent was removed under reduced pressure (waterbath temperature max. 30 °C; pressure max. 45 mbar). The resulting residue was filtered over $MgSO_4$ and washed with Et_2O. Again, the solvent was removed under reduced pressure (water bath temperature max. 30 °C; pressure

max. 45 mbar). The product **S13** was used without further purifications. The spectroscopic data were in accordance with those reported in the literature.[S10]

^{1}H-NMR (400 MHz, $CDCl_3$, $CHCl_3$ = 7.26 ppm): δ = 4.41 (2H, m, H-2), 1.13 (2H, m, H-3), 0.06 (9H, s, H-4) ppm; ^{13}C-NMR (100 MHz, $CDCl_3$, $CDCl_3$ = 77.16 ppm): δ = 156.7 (q, C-1), 71.5 (s, C-2), 17.6 (s, C-3), -1.5 (3C, p, C-4) ppm.

Synthesis of 2,5-dioxopyrrolidin-1-yl [2(trimethylsilyl)ethyl] carbonate (S14)

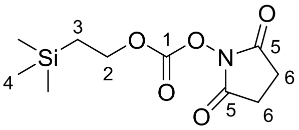

TeocCl reagent **S13** was dissolved in CH_3CN (105 mL) at 0 °C. Then, NHS (5.22 g, 45.4 mmol, 1.30 equiv) and Et_3N (4.59 g, 45.4 mmol, 1.30 equiv; in 11.0 mL CH_3CN) were added. The reaction mixture was stirred at 0 °C → rt. After 16 h, the reaction mixture was poured into H_2O. The aqueous layer was extracted with Et_2O (6×). The combined organic layers were washed with H_2O (2×), HCl (1.0 M), followed by H_2O and subsequent dried over $MgSO_4$, filtered a well as concentrated under reduced pressure. The desired TeocOSu **S14** (7.72 g, 29.8 mmol, 85% over two steps) was collected as colourless solid. The spectroscopic and physical data were in accordance with those reported in the literature.[S10]

R_f = 0.72 (petroleum ether/ethyl acetate = 1:1); mp = 92 – 97 °C (mp = 98 – 99 °C, ref.[S10]); ^{1}H-NMR (400 MHz, $CDCl_3$, $CHCl_3$ = 7.26 ppm): δ = 4.41 (2H, m, H-2), 2.83 (4H, s, H-6), 1.15 (2H, m, H-3), 0.07 (9H, s, H-4) ppm; ^{13}C-NMR (100 MHz, $CDCl_3$, $CDCl_3$ = 77.16 ppm): δ = 168.9 (2C, q, C-5), 151.6 (q, C-1), 70.8 (s, C-2), 25.6 (2C, s, C-6), 17.7 (s, C-3), -1.5 (3C, p, C-4) ppm.

Synthesis of [(2-(trimethylsilyl)ethoxy)carbonyl]-L-valine (S15)

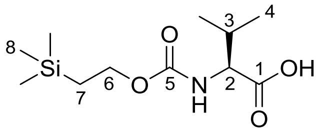

L-Valine (1.17 g, 10.0 mmol, 1.00 equiv) was dissolved in H_2O (10.0 mL) at rt. Then, Et_3N (1.52 g, 15.0 mmol, 1.50 equiv) in 1,4-dioxane (10.0 mL) was added, followed by TeocOSu **S14** (2.85 g, 11.0 mmol, 1.10 equiv). The reaction mixture was stirred for 15 h at rt, then diluted with H_2O and acidified with saturated $KHSO_4$ solution. Afterwards, the reaction mixture was extracted with EtOAc (6×). The combined organic layers were washed with H_2O (4x) and subsequently dried over $MgSO_4$, filtered as well as concentrated under reduced pressure. The product was directly used in the next step without further purifications.

^{1}H-NMR (200 MHz, CD_3OD, CD_3OH = 3.31 ppm): δ = 4.15 (2H, m, H-6), 4.05 (1H, d, J = 6.8 Hz, H-2), 2.15 (1H, m, H-3), 1.02 (2H, m, H-7), 0.98 (3H, d, J = 6.8 Hz, H-4), 0.94 (3H, d, J =

6.8 Hz, H-4), 0.05 (9H, s, H-8) ppm; HRMS (ESI): *m/z* calculated for $C_{11}H_{23}NO_4SiNa$ [M + Na]$^+$: 284.1294; found 284.1295.

Synthesis of 2-(trimethylsilyl)ethyl (*S*)-(1-amino-3-methyl-1-oxobutan-2-yl)carbamate (38)

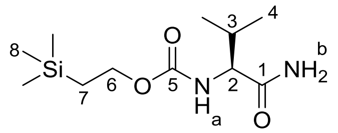

Teoc-L-valine **S15** and *N*-methylmorpholine (1.09 g, 10.8 mmol, 1.08 equiv) were dissolved in 1,2-dimethoxyethane (51.0 mL) at 0 °C. Then, isobutylchloroformiate (1.48 g, 10.8 mmol, 1.08 equiv) was added dropwise. The reaction mixture was stirred for 20 min at 0 °C. Afterwards, an aqueous ammonia solution (25%; 5.00 mL, 65.7 mmol 6.57 equiv) was added dropwise. The reaction mixture was warmed up to rt. After 23 h, the reaction was terminated by addition of HCl (1.0 M). The aqueous layer was extracted with EtOAc (7×). The combined organic layers were washed with HCl (0.1 M) and subsequently dried over $MgSO_4$, filtered and concentrated under reduced pressure. Amide **38** (2.60 g, 10.0 mmol, quantitative over two steps) was obtained as a colourless solid.
mp= 138 – 139 °C; $[\alpha]_D^{20}$= -4.5 (*c* 1.21, CH_3OH); ^{1}H-NMR (400 MHz, DMSO-d_6, DMSO-d_5 = 2.50 ppm): δ = 7.28 (1H, s, NH-b), 7.00 (1H, s, NH-b), 6.80 (1H, d, *J* = 8.8 Hz, NH-a), 4.03 (2H, m, H-6), 3.77 (1H, dd, *J* = 6.8, 8.8 Hz, H-2), 1.92 (1H, m, H-3), 0.92 (2H, t, *J* = 8.4 Hz, H-7), 0.85 (3H, d, *J* = 6.8 Hz, H-4), 0.82 (3H, d, *J* = 6.8 Hz, H-4), 0.02 (9H, s, H-8) ppm; ^{13}C-NMR (100 MHz, DMSO-d_6, DMSO-d_6 = 39.52 ppm): δ = 173.3 (q, C-1), 156.3 (q, C-5), 61.7 (s, C-6), 59.8 (t, C-2), 30.1 (t, C-3), 19.3 (s, C-7), 17.9 (p, C-4), 17.4 (p, C-4), -1.4 (3C, p, C-8) ppm; HRMS (ESI): *m/z* calculated for $C_{11}H_{24}N_2O_3SiNa$ [M + Na]$^+$: 283.1454; found 283.1457.

Synthesis of methyl (8*S*,14*R*)-14-{3-[(*tert*-butoxycarbonyl)amino]propyl}-8-isopropyl-2,2-dimethyl-6,9,12-trioxo-11-(propan-2-ylidene)-5-oxa-7,10,13-triaza-2-silapentadecan-15-oate (39)

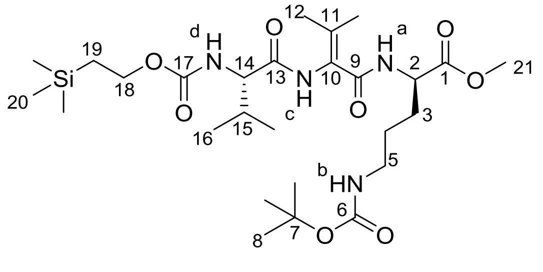

Teoc-L-Val-NH_2 (**38**; 30.7 mg, 0.12 mmol, 1.00 equiv) and vinyl iodide **29** (107 mg, 0.24 mmol, 2.00 equiv) were dissolved in 1,4-dioxane (degassed; 0.24 mL) at rt. Then, K_2CO_3 (32.6 mg, 0.24 mmol, 2.00 equiv), CuI (13.5 mg, 70.9 µmol, 0.60 equiv) and *trans*-

N,*N*-dimethyl-1,2-cyclohexanediamine (67.9 mg, 0.48 mmol, 4.05 equiv) were added. The reaction mixture was heated to 70 °C. After 18 h, the reaction mixture was terminated by adding a saturated NH_4Cl solution. Then, the aqueous layer was extracted with EtOAc (8×). The combined organic layers were washed with brine and dried over $MgSO_4$, filtered and concentrated under reduced pressure. The resulting residue was dissolved in CH_3OH (1.8 mL) and purified by preparative HPLC (C18) (gradient CH_3OH/H_2O 10:90 {5 min} → CH_3OH/H_2O = 100:0 {85 min} → CH_3OH/H_2O = 100:0 {10 min}, 15 mL/min) (t_R = 79.9 min). The desired tripeptide **39** (22.7 mg, 38.7 µmol, 33%) was obtained as a colourless foam.
$[\alpha]_D^{20}$ = -11.5 (*c* = 1.39, CH_2Cl_2); ^{1}H-NMR (400 MHz, $CDCl_3$, $CHCl_3$ = 7.26 ppm): δ = 7.52 (1H, brs, NH-c), 7.01 (1H, brm, NH-a), 5.24 (1H, brm, NH-d), 4.91 (1H, brm, NH-b), 4.59 (1H, m, H-2), 4.17 (2H, m, H-18), 3.95 (1H, t, *J* = 6.8 Hz, H-14), 3.71 (3H, s, H-21), 3.12 (2H, m, H-5), 2.15 (1H, m, H-15), 2.09 (3H, s, H-12), 1.91 (1H, m, H-3), 1.76 (3H, s, H-12), 1.73 (1H, m, H-3‘), 1.57 (2H, m, H-4), 1.42 (9H, s, H-8), 1.04-0.98 (8H, m, H-16, H-19), 0.03 (9H, s, H-20) ppm; ^{13}C-NMR (100 MHz, $CDCl_3$, $CDCl_3$ = 77.16 ppm): δ = 172.8 (q, C-1), 171.2 (q, C-13), 165.8 (q, C-9), 157.5 (q, C-17), 156.3 (q, C-6), 141.5 (q, C-10), 123.4 (q, C-11), 79.2 (q, C-7), 64.2 (s, C-18), 61.3 (t, C-14), 52.4 (p, C-21), 52.1 (t, C-2), 40.2 (s, C-5), 30.2 (t, C-15), 29.2 (s, C-3), 28.6 (3C, p, C-8), 25.9 (s, C-4), 21.6 (p, C-12), 21.0 (p, C-12), 19.6 (s, C-19), 18.2 (p, C-16), 17.9 (p, C-16), -1.4 (3C, p, C-20) ppm; HRMS (ESI): *m/z* calculated for $C_{27}H_{50}N_4O_8SiNa$ $[M + Na]^+$: 609.3296; found 609.3298.

Synthesis of methyl [(*R*)-5-{(*tert*-butoxycarbonyl)amino}-2-(3-methyl-2-[(*S*)-3-methyl-2-{[(2-(trimethylsilyl)ethoxy)carbonyl]amino}butanamido]but-2-enamido)pentanoyl]-D-valinate (40)

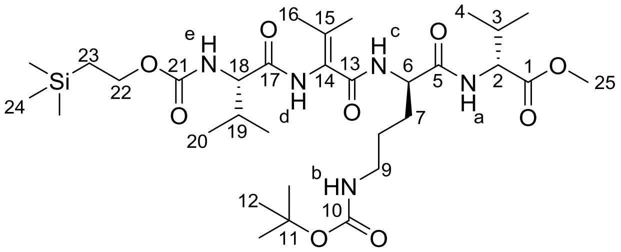

Teoc-L-Val-NH_2 (**38**; 31.6 mg, 0.12 mmol, 1.00 equiv) and vinyl iodide **35** (134 mg, 0.24 mmol, 2.00 equiv) were dissolved in 1,4-dioxane (degassed; 0.25 mL) at rt. Then, K_2CO_3 (33.5 mg, 0.24 mmol, 2.00 equiv), CuI (13.9 mg, 73.0 µmol, 0.60 equiv) and *trans*-*N*,*N*-dimethyl-1,2-cyclohexanediamine (69.9 mg, 0.49 mmol, 4.05 equiv) were added. The reaction mixture was heated to 70 °C. After 22 h, the reaction mixture was terminated by adding a saturated NH_4Cl solution. Then, the aqueous layer was extracted with EtOAc (8×). The combined organic layers were washed with brine and subsequently dried over $MgSO_4$, filtered and concentrated under reduced pressure. The resulting residue was dissolved in CH_3OH (1.8 mL) and purified by preparative HPLC (C18) (gradient CH_3OH/H_2O 10:90 {5

min} → CH_3OH/H_2O = 100:0 {85 min} → CH_3OH/H_2O = 100:0 {10 min}, 15 mL/min) (t_R = 81.9 min). The desired tetrapeptide **40** (38.7 mg, 56.4 µmol, 47%) was obtained as a colourless foam.

$[\alpha]_D^{20}$ = +3.5 (*c* = 1.00, CH_2Cl_2); ^{1}H-NMR (500 MHz, $CDCl_3$, $CHCl_3$ = 7.26 ppm): δ = 7.57 (1H, s, NH-d), 7.06 (1H, d, *J* = 7.5 Hz, NH-c), 7.03 (1H, d, *J* = 8.6 Hz, NH-a), 5.28 (1H, d, *J* = 6.8 Hz, NH-e), 4.88 (1H, brm, NH-b), 4.52 (1H, m, H-6), 4.44 (1H, dd, *J* = 5.3, 7.8 Hz, H-2), 4.15 (2H, m, H-22), 3.92 (1H, t, *J* = 6.8 Hz, H-18), 3.70 (3H, s, H-25), 3.13 (2H, m, H-9), 2.16 (2H, m, H-3, H-19), 2.10 (3H, s, H-16), 1.92 (1H, m, H-7), 1.78 (3H, s, H-16), 1.74 (1H, m, H-7‘). 1.59 (2H, m, H-8), 1.42 (9H, s, H-12), 1.03 (6H, d, *J* = 6.8 Hz, H-4), 0.98 (2H, d, *J* = 8.1 Hz, H-23), 0.91 (6H, d, *J* = 6.8 Hz, H-20), 0.03 (9H, s, H-24) ppm; ^{13}C-NMR (125 MHz, $CDCl_3$, $CDCl_3$ = 77.16 ppm): δ = 172.3 (q, C-1), 171.8 (q, C-5), 171.3 (q, C-17), 166.1 (q, C-13), 157.5 (q, C-21), 156.5 (q, C-10), 141.2 (q, C-14), 123.5 (q, C-15), 79.2 (q, C-11), 64.2 (s, C-22), 61.2 (t, C-18), 57.6 (t, C-2), 52.9 (t, C-6), 52.2 (p, C-25), 40.0 (s, C-9), 31.0 (t, C-3 o. C-19), 30.3 (t, C-3 o. C-19), 28.6 (3C, p, C-12), 28.4 (s, C-7), 26.2 (s, C-8), 21.7 (p, C-16), 21.0 (p, C-16), 19.7 (s, C-23), 19.2 (p, C-4), 18.3 (p, C-4), 18.2 (p, C-20), 17.8 (p, C-20), -1.3 (3C, p, C-24) ppm; HRMS (ESI): *m/z* calculated for $C_{32}H_{59}N_5O_9SiNa$ $[M + Na]^+$: 708.3980; found 708.3982.

Synthesis of methyl *N*²-(3-methyl-2-[(*S*)-3-methyl-2-{[(2-(trimethylsilyl)ethoxy)-carbonyl]amino}butanamido]but-2-enoyl)-*N*^ω^-[(2,2,4,6,7-pentamethyl-2,3-dihydrobenzo-furane-5-yl)sulfonyl]-D-arginyl-D-valinate (41)

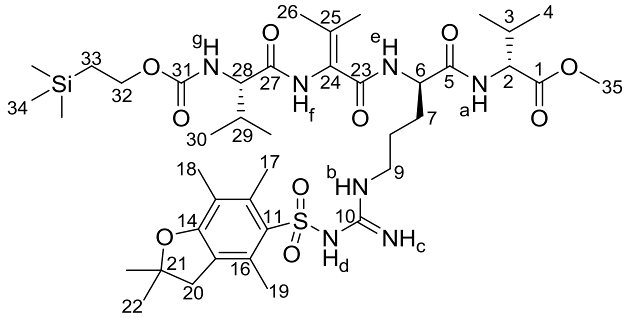

Teoc-L-Val-NH_2 (**38**; 43.5 mg, 0.17 mmol, 1.00 equiv) and vinyl iodide **27** (250 mg, 0.33 mmol, 2.00 equiv) were dissolved in 1,4-dioxane (degassed; 0.17 mL) at rt. Then, K_2CO_3 (46.2 mg, 0.33 mmol, 2.00 equiv), CuI (19.1 mg, 0.10 mmol, 0.60 equiv) and *trans*-*N*,*N*-dimethyl-1,2-cyclohexanediamine (96.3 mg, 0.68 mmol, 4.05 equiv) were added. The reaction mixture was heated to 70 °C. After 22 h, the reaction mixture was terminated by adding a saturated NH_4Cl solution. Then, the aqueous layer was extracted with EtOAc (6×). The combined organic layers were washed with brine and subsequently dried over $MgSO_4$, filtered and concentrated under reduced pressure. The resulting residue was dissolved in

CH_3OH (1.8 mL) and purified by preparative HPLC (C18) (Gradient CH_3OH/H_2O 10:90 {5 min} → CH_3OH/H_2O = 100:0 {85 min} → CH_3OH/H_2O = 100:0 {10 min}, 15.0 mL/min) (t_R = 85.5 min). The desired dehydropeptide **41** (3.70 mg, 4.20 µmol, 2.5%) was obtained as a colourless foam.

^{1}H-NMR (500 MHz, $CDCl_3$, $CHCl_3$ = 7.26 ppm): δ = 8.16 (1H, s, NH-f), 7.22 (1H, m, NH-a), 7.13 (1H, m, NH-e), 6.34 (2H, brs, NH-b, NH-d), 5.32 (1H, m, NH-g), 4.50 (1H, m, H-6), 4.37 (1H, m, H-2), 4.17 (1H, m, H-32), 4.06 (1H, m, H-32‘), 4.01 (1H, m, H-28), 3.70 (3H, s, H-35), 3.21 (2H, m, H-9), 2.95 (2H, s, H-20), 2.57 (3H, s, H-17 o. H-19), 2.51 (3H, s, H-17 o. H-19), 2.16 (2H, m, H-3, H-29), 2.09 (6H, s, H-18, H-26), 1.98 (1H, m, H-7), 1.80 (3H, s, H-26), 1.70-1.63 (3H, m, H-7‘, H-8), 1.46 (6H, s, H-22), 1.03-0.90 (14H, m, H-4, H-30, H-33), 0.02 (9H, s, H-34) ppm; HRMS (ESI): *m/z* calculated for $C_{41}H_{69}N_7O_{10}SSiNa$ $[M + Na]^+$: 902.4494; found 902.4488.

Signals of NH^c-proton could not be detected in the ^{1}H NMR spectrum.

Synthesis of methyl (*E*)-(2-iodo-3-methylpent-2-enoyl)-D-alaninate (42)

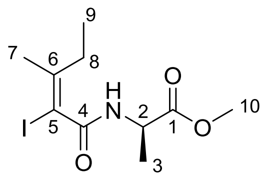

Acid **11** (268 mg, 1.12 mmol, 1.00 equiv) and D-alanine methylester hydrochloride (202 mg, 1.45 mmol, 1.30 equiv) were dissolved in DMF (6.10 mL) at 0 °C. Then, HOAt (162 mg, 1.19 mmol, 1.07 equiv) and PyAOP (610 mg, 1.17 mmol, 1.05 equiv) were added. Afterwards, DIPEA (662 mg, 5.12 mmol, 4.60 equiv) was added dropwise. The reaction mixture was warmed up to rt. After 22 h, the reaction was terminated by addition of a saturated NH_4Cl solution. The layers were separated and the aqueous layer was extracted with EtOAc (7×). The combined, organic layers were washed with brine and subsequently dried over Na_2SO_4, filtered and concentrated under reduced pressure. Purification by flash column chromatography over silica (petroleum ether/ ethyl acetate= 4:1) yielded the desired product **42** (210 mg, 0.65 mmol, 58%) as a colourless solid.

R_f = 0.73 (CH_2Cl_2/CH_3OH 98:2); mp = 58 – 60 °C; $[\alpha]_D^{20}$ = +12.5 (*c* = 1.00, CH_2Cl_2); ^{1}H-NMR (400 MHz, $CDCl_3$, $CHCl_3$ = 7.26 ppm): δ = 6.26 (1H, brm, NH), 4.59 (1H, m, H-2), 3.75 (3H, s, H-10), 2.38 (2H, q, *J* = 7.4 Hz, H-8), 1.96 (3H, s, H-7), 1.42 (3H, d, *J* = 7.2 Hz, H-3), 1.05 (3H, t, *J* = 7.4 Hz, H-9) ppm; ^{13}C-NMR (100 MHz, $CDCl_3$, $CDCl_3$ = 77.16 ppm): δ = 173.2 (q, C-1), 166.7 (q, C-4), 151.0 (q, C-6), 87.0 (q, C-5), 52.6 (p, C-10), 48.5 (t, C-2), 29.3 (p, C-7), 27.2 (s, C-8), 18.2 (p, C-3), 13.2 (p, C-9) ppm; HRMS (ESI): *m/z* calculated for $C_{10}H_{16}INO_3Na$ $[M + Na]^+$: 348.0073; found 348.0075.

Synthesis of *tert*-butyl (*R*)-1-(1-amino-3-methyl-1-oxobutan-2-yl)carbamate (43)

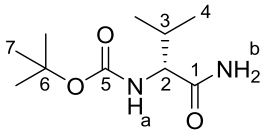

Boc-D-valine (2.00 g, 9.21 mmol, 1.00 equiv) and *N*-methylmorpholine (1.01 g, 9.94 mmol, 1.08 equiv) were dissolved in 1,2-dimethoxyethane (47.0 mL) at 0 °C. Then, isobutylchloroformiate (1.36 g, 9.94 mmol, 1.08 equiv) was added dropwise. The reaction mixture was stirred for 15 min at 0 °C. Afterwards, an aqueous ammonia solution (25%; 4.10 mL, 60.5 mmol, 6.57 equiv) was added dropwise. Now, the reaction mixture was stirred at 0 °C → rt. After 20 h, the reaction was terminated by addition of HCl (1.0 M). The aqueous layer was extracted with EtOAc (5×). The combined organic layers were washed with HCl (0.1 M) and dried over $MgSO_4$, filtered and concentrated under reduced pressure. Amide **43** (1.99 g, 9.21 mmol, quantitative) was obtained as a colourless solid. The analytical data were in accordance with those reported in the literature[S2].

R_f = 0.20 (CH_2Cl_2/CH_3OH 98:2); mp = 155 -157 °C (mp = 156 – 157 °C, ref.[S11]); $[\alpha]_D^{20}$ = +2.5 (*c* 1.14, CH_3OH) {$[\alpha]_D^{20}$ = +2.5 (*c* 1.05, CH_3OH) ref.[S12]}; ^{1}H-NMR (400 MHz, $CDCl_3$, $CHCl_3$ = 7.26 ppm): δ = 6.22 (1H, brs, NH-b), 5.82 (1H, brs, NH-b), 5.14 (1H, d, *J* = 8.5 Hz, NH-a), 3.98 (1H, dd, *J* = 6.6, 8.5 Hz, H-2), 2.12 (1H, m, H-3), 1.44 (9H, s, H-7), 0.98 (3H, d, *J* = 6.6 Hz, H-4), 0.94 (3H, d, *J* = 6.6 Hz, H-4) ppm; ^{13}C-NMR (100 MHz, $CDCl_3$, $CDCl_3$= 77.16 ppm): δ = 174.4 (q, C-1), 156.1 (q, C-5), 80.1 (q, C-6), 59.6 (t, C-2), 30.8 (t, C-3), 28.5 (3C, p, C-7), 19.4 (2C, p, C-4) ppm; HRMS (ESI): *m/z* calculated for $C_{10}H_{20}N_2O_3Na$ $[M + Na]^+$: 239.1372; found 239.1369.

Synthesis of methyl {(*E*)-2-[(*R*)-2-[(*tert*-butoxycarbonyl)amino]-3-methylbutanamido]-3-methylpent-2-enoyl}-D-alaninate (44)

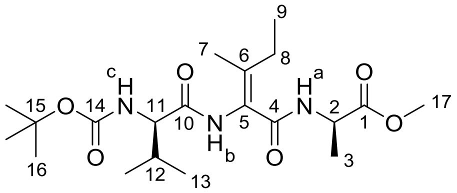

Boc-D-Val-NH_2 (**43**; 82.1 mg, 0.38 mmol, 1.00 equiv) and vinyl iodide **42** (245 mg, 0.76 mmol, 2.00 equiv) were dissolved in 1,4-dioxane (degassed; 0.38 mL) at rt. Then, K_2CO_3 (105 mg, 0.76 mmol, 2.00 equiv), CuI (43.4 mg, 0.23 mmol, 0.60 equiv) und *trans*-*N*,*N*-dimethyl-1,2-cyclohexanediamine (216 mg, 1.52 mmol, 4.05 equiv) were added. The reaction mixture was heated to 70 °C. After 20 h, the solvent was removed under reduced pressure. The resulting residue was diluted with a saturated NH_4Cl solution. Then, the aqueous layer was extracted with EtOAc (4×). The combined organic layers were washed with brine and dried over $MgSO_4$, filtered and concentrated under reduced pressure.

Purification by flash column chromatography over silica (100% CH_2Cl_2 → CH_2Cl_2/CH_3OH = 99:1 → CH_2Cl_2/CH_3OH = 98:2) gave the desired dehydropeptide **44** (75.4 mg, 0.18 mmol, 48%) as a colourless solid.

R_f = 0.27 (CH_2Cl_2/CH_3OH = 98:2); mp> 177 °C (decomposition); $[\alpha]_D^{20}$ = +21.2 (*c* 1.82, CH_2Cl_2); ^{1}H-NMR (400 MHz, $CDCl_3$, $CHCl_3$ = 7.26 ppm): δ = 7.47 (1H, brs, NH-b), 7.02 (1H, d, *J* = 6.2 Hz, NH-a), 5.08 (1H, d, *J* = 7.2 Hz, NH-c), 4.58 (1H, m, H-2), 3.91 (1H, m, H-11), 3.72 (3H, s, H-17), 2.48-2.30 (2H, m, H-8), 2.16 (1H, m, H-12), 1.72 (3H, s, H-7), 1.42-1.40 (12H, m, H-3, H-16), 1.09 (3H, t, *J* = 7.5 Hz, H-9), 1.00 (3H, d, *J* = 6.8 Hz, H-13), 0.96 (3H, d, *J* = 6.8 Hz, H-13) ppm; ^{13}C-NMR (100 MHz, $CDCl_3$, $CDCl_3$ = 77.16 ppm): δ = 173.4 (q, C-1), 171.5 (q, C-10), 165.9 (q, C-4), 156.2 (q, C-14), 144.5 (q, C-6), 123.8 (q, C-5), 80.4 (q, C-15), 60.6 (t, C-11), 52.5 (p, C-17), 48.4 (t, C-2), 30.5 (t, C-12), 28.4 (3C, p, C-16), 27.3 (s, C-8), 19.5 (2C, p, C-13), 18.2 (p, C-3), 18.0 (p, C-7), 12.9 (p, C-9) ppm; HRMS (ESI): *m/z* calculated for $C_{20}H_{35}N_3O_6Na$ $[M + Na]^+$: 436.2424; found 436.2425.

nOe (500 MHz, $CDCl_3$):

proton	nOe contact
H-2	H-8α, H-8β, NH-a
H-3	H-2, NH-a
H-7	H-8α, H-8β, H-9, NH-b
H-8α	H-7, H-8β, NH-a
H-8β	H-8α, NH-a
H-9	H-7, H-8α, H-8β, NH-a
H-11	H-12, H-13, NH-b, NH-c
H-12	H-11, H-13, NH-c
NH-a	H-2, H-8 (w)
NH-b	H-7, H-11, H-12 (w)

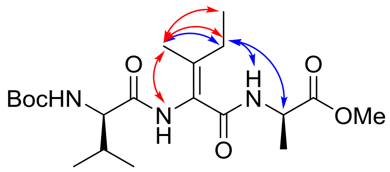

Figure S1: Structure relevante nOe contacts of **44**; red: contacts of methyl group, blue: contacts of methylene group.

References supporting information

[S1] Gottlieb, H. E.; Kotlyar, V.; Nudelman, A. *J. Org. Chem.* **1997**, *62*, 7512-7515.

[S2] Kuranaga, T.; Sesoko, Y.; Sakata, K.; Maeda, N.; Hayata, A.; Inoue, M. *J. Am. Chem. Soc.* **2013**, *135*, 5467-5474.

[S3] Gagnier, S. V.; Larock, R. C. *J. Org. Chem.* **2000**, *65*, 1525-1529.

[S4] Zapf, C. W.; Goodman, M. *J. Org. Chem.* **2003**, *68*, 10092-10097.

[S5] Feichtinger, K.; Zapf C. W.; Sings, H. L.; Goodman, M. *J. Org. Chem.* **1998**, *63*, 3804-3805.

[S6] Merritt, E. A.; Bagley, M. C. *Synthesis* **2007**, *22*, 3535-3541.

[S7] Mangette, J. E.; Johnson, M. R.; Le, V.-D.; Shenoy, R. A.; Roark, H.; Stier, M.; Belliotti, T.; Capiris, T.; . Guzzo, P. R.*Tetrahedron* **2009**, *65*, 9536-9541.

[S8] Debaene, F.; Mejias, L.; Harris, J. L.; Winssinger, N. *Tetrahedron* **2004**, *60*, 8677-8690.

[S9] Chinchilla, R.; Dodsworth, D. J.; Nájera, C.; Soriano, J. M. *Synlett* **2003**, *6*, 809-812.

[S10] Shute, R. E.; Rich, D. H. *Synthesis* **1987**, 346-349.

[S11] Xa, Z.; Smith, C. D. *J. Org. Chem.* **2001**, *66*, 3459-3466.

[S12] Ciufolini, M. A.; Shen, Y.–C. *Org. Lett.* **1999**, *1*, 1843-1846.

Copies of ^{1}H and ^{13}C-NMR spectra

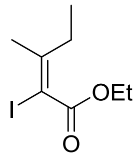

S1

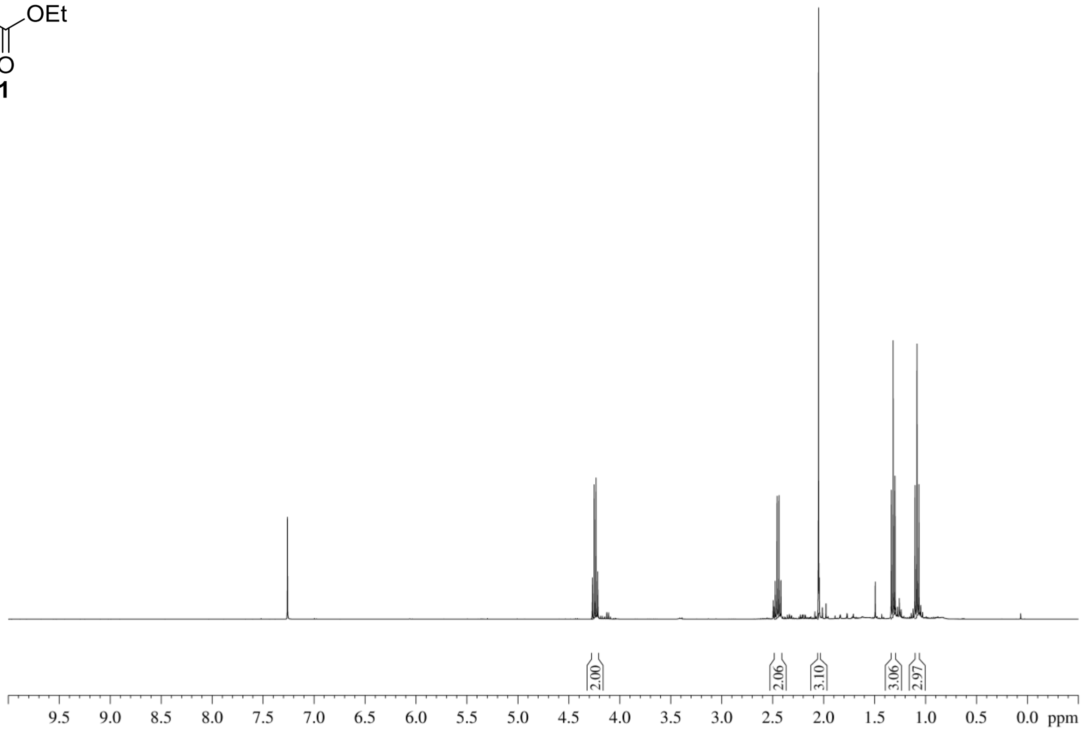

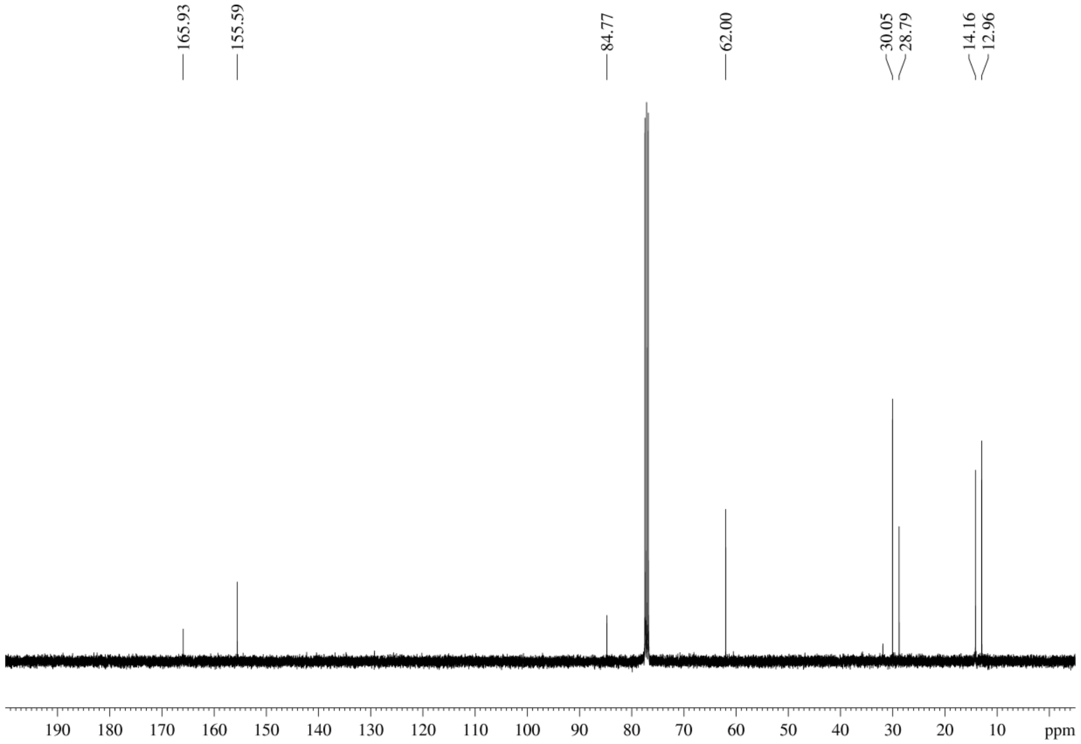

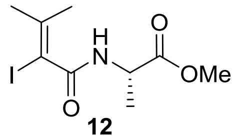
H
N
O
O
I
OMe

12

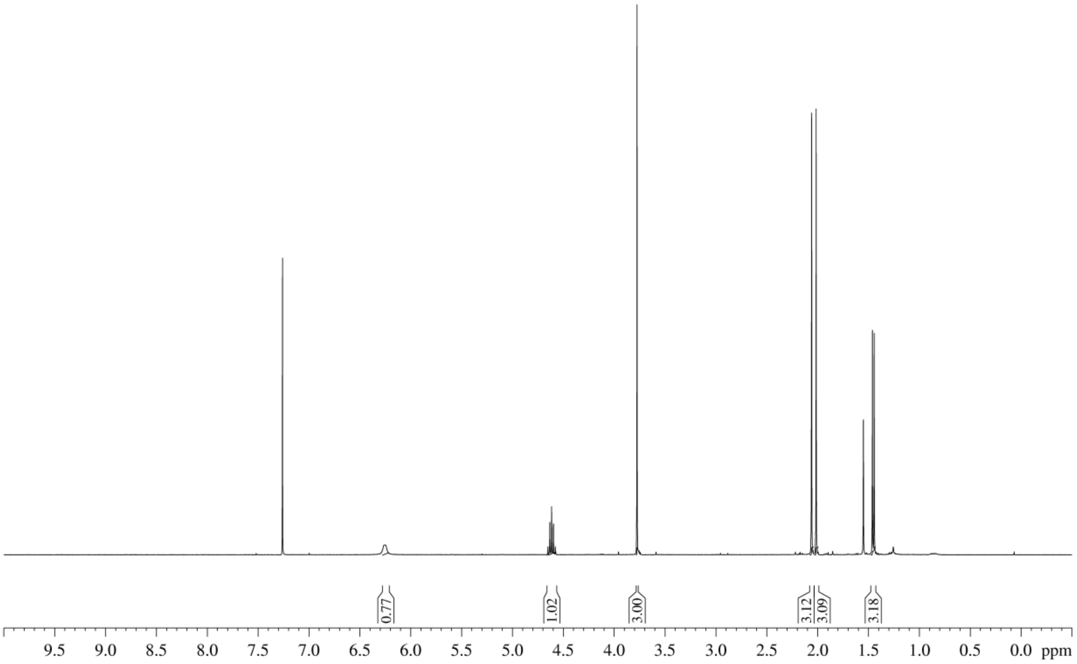
0.77
1.02
3.00
3.12
3.09
3.18
9.5 9.0 8.5 8.0 7.5 7.0 6.5 6.0 5.5 5.0 4.5 4.0 3.5 3.0 2.5 2.0 1.5 1.0 0.5 0.0 ppm

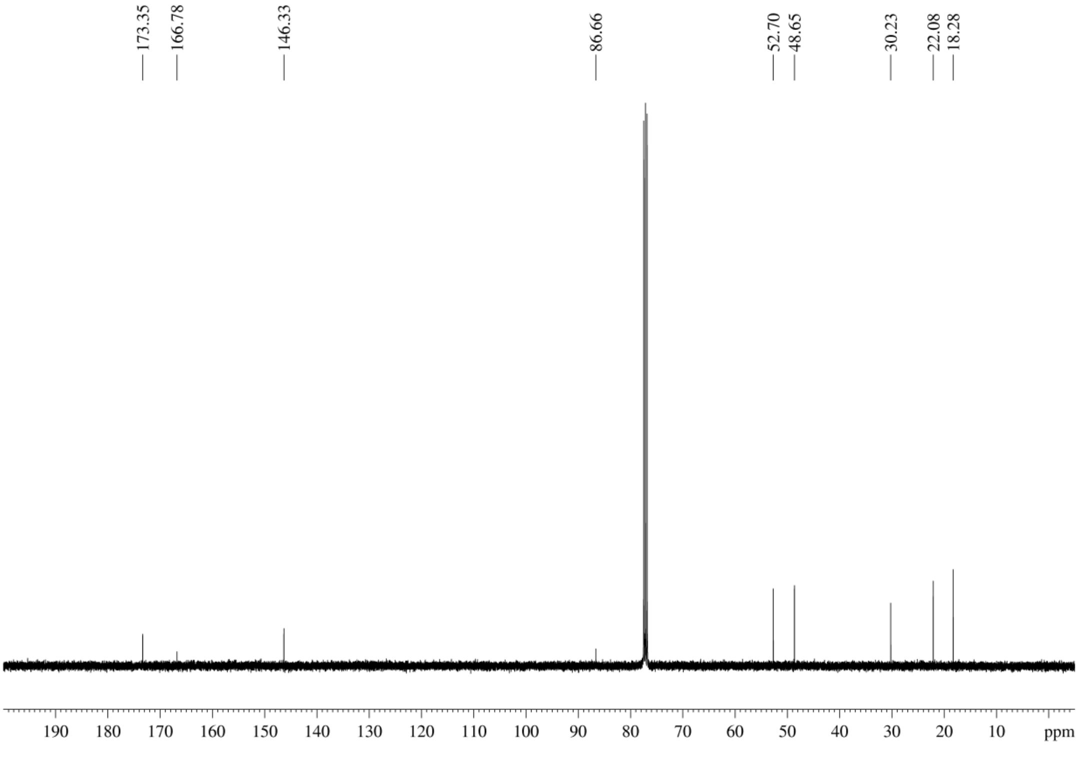
173.35
166.78
146.33
86.66
52.70
48.65
30.23
22.08
18.28
190 180 170 160 150 140 130 120 110 100 90 80 70 60 50 40 30 20 10 ppm

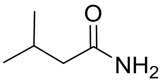
O
NH2

13

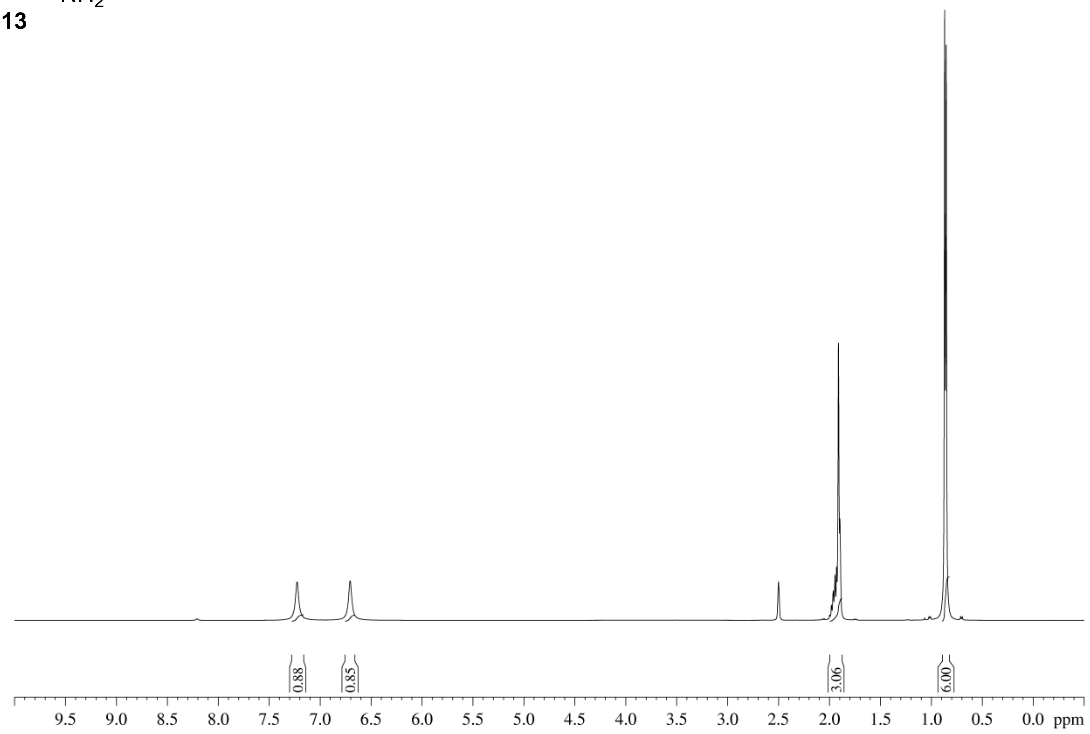
0.88
0.85
3.06
6.00
9.5 9.0 8.5 8.0 7.5 7.0 6.5 6.0 5.5 5.0 4.5 4.0 3.5 3.0 2.5 2.0 1.5 1.0 0.5 0.0 ppm

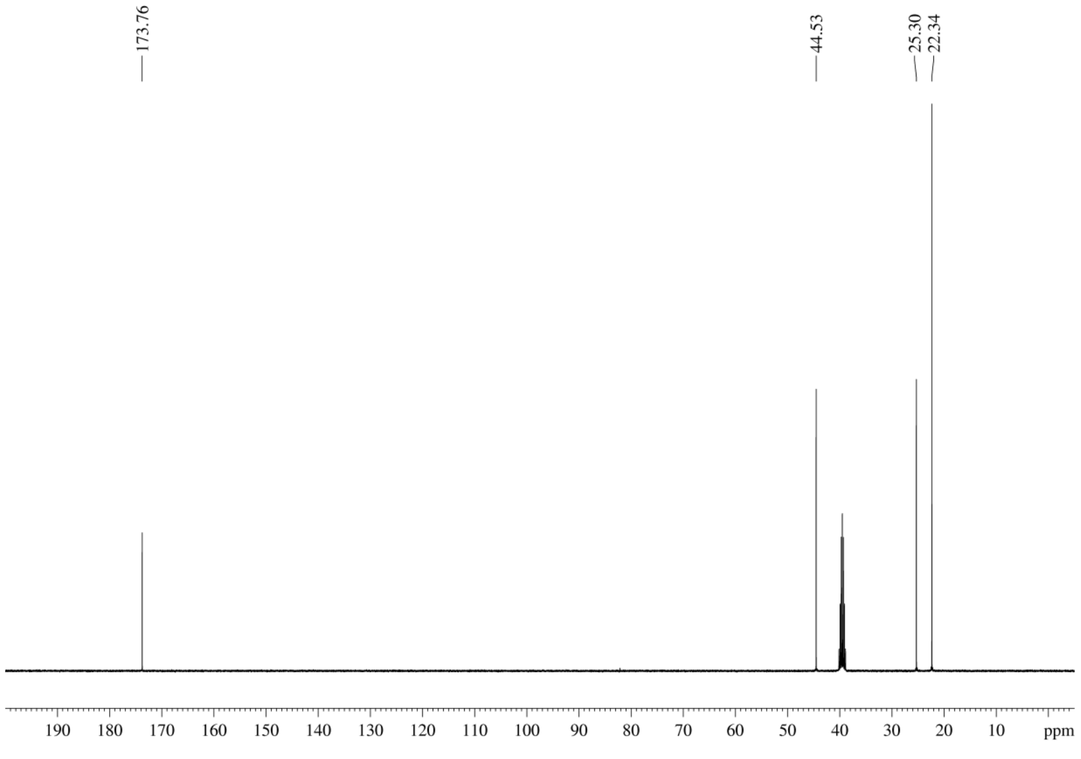
173.76
44.53
25.30
22.34
190 180 170 160 150 140 130 120 110 100 90 80 70 60 50 40 30 20 10 ppm

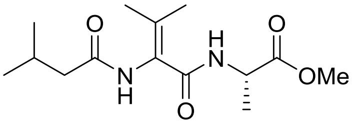
O
H
N
OMe
O
N
H
O

14

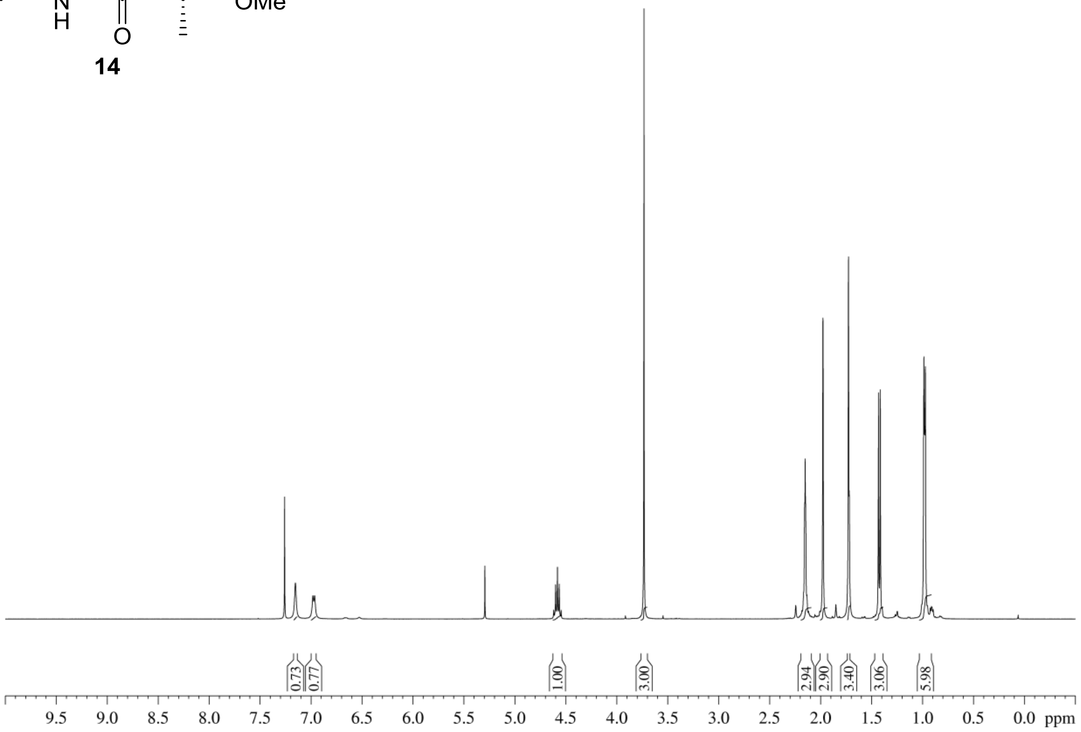
0.73
0.77
1.00
3.00
2.94
2.90
3.40
3.06
5.98
9.5 9.0 8.5 8.0 7.5 7.0 6.5 6.0 5.5 5.0 4.5 4.0 3.5 3.0 2.5 2.0 1.5 1.0 0.5 0.0 ppm

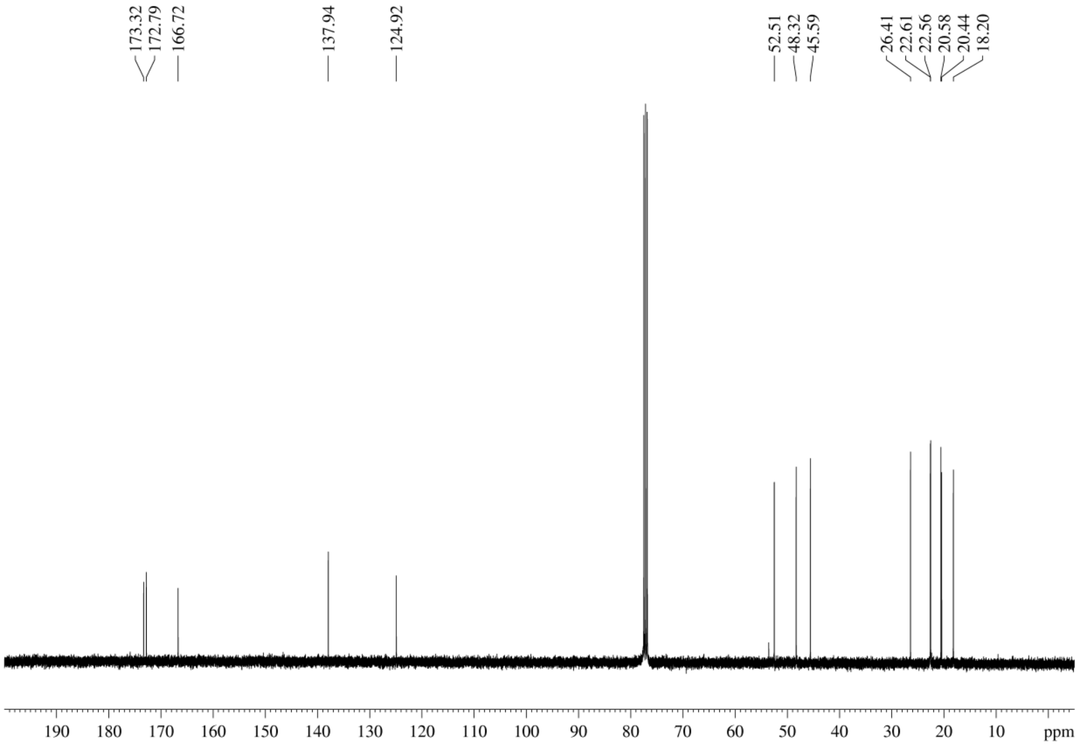
173.32
172.79
166.72
137.94
124.92
52.51
48.32
45.59
26.41
22.61
22.56
20.58
20.44
18.20
190 180 170 160 150 140 130 120 110 100 90 80 70 60 50 40 30 20 10 ppm

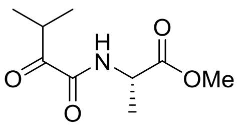
H
O
O
O
OMe

15

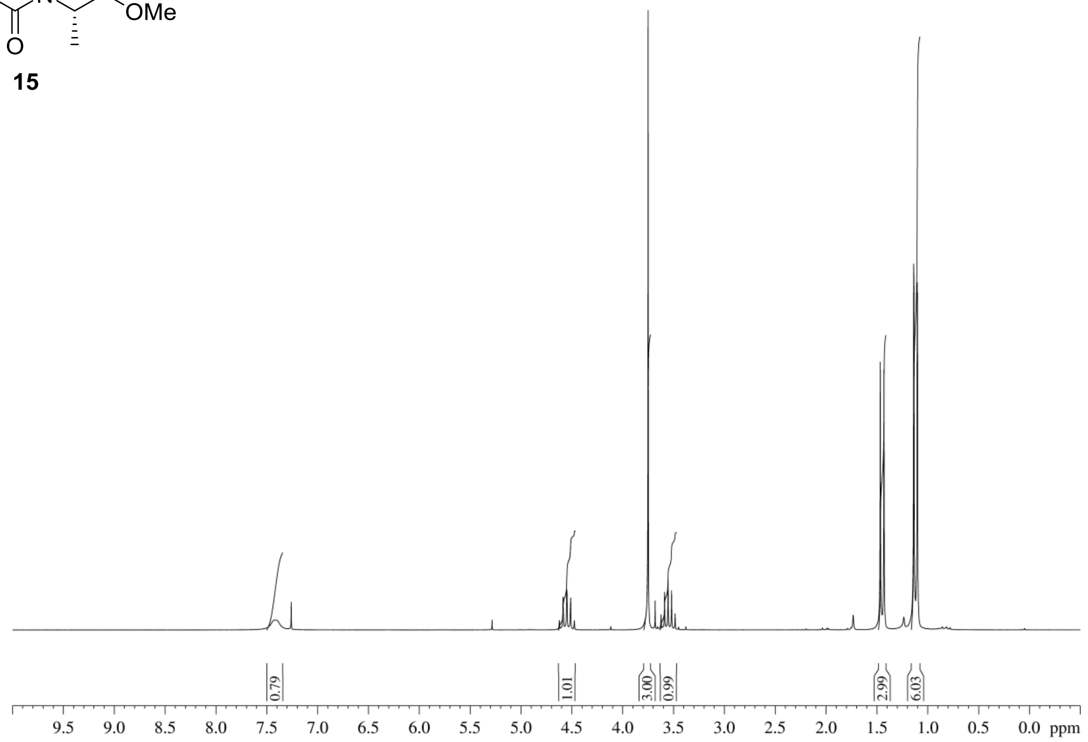
0.79
1.01
3.00
0.99
2.99
6.03
9.5 9.0 8.5 8.0 7.5 7.0 6.5 6.0 5.5 5.0 4.5 4.0 3.5 3.0 2.5 2.0 1.5 1.0 0.5 0.0 ppm

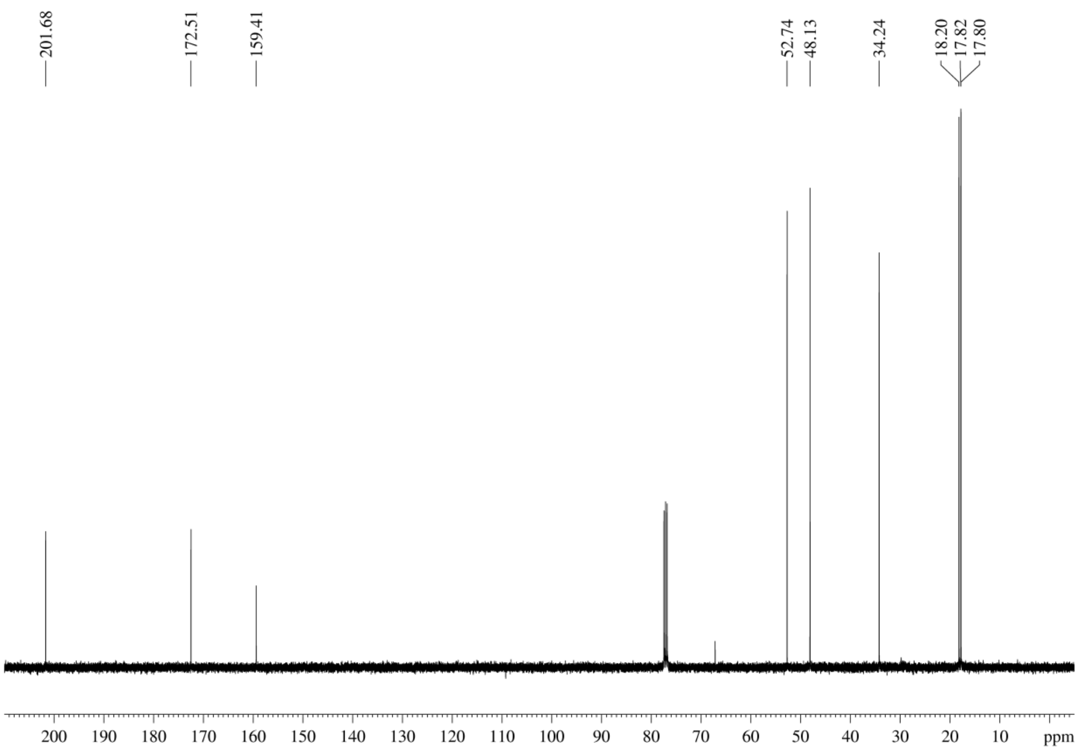
201.68
172.51
159.41
52.74
48.13
34.24
18.20
17.82
17.80
200 190 180 170 160 150 140 130 120 110 100 90 80 70 60 50 40 30 20 10 ppm

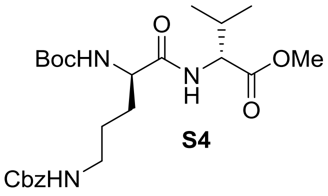
BocHN
OMe
N
H
O
O
CbzHN

S4

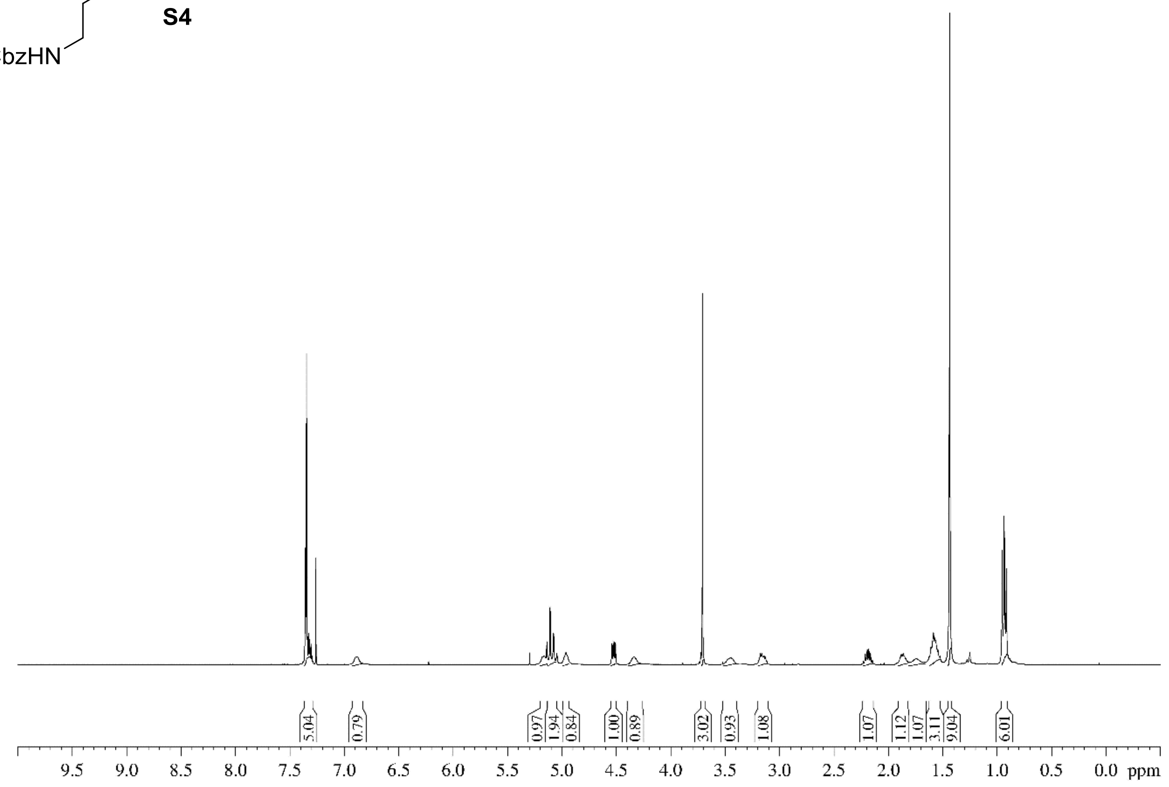
5.04
0.79
0.97
1.94
0.84
1.00
0.89
3.02
0.93
1.08
1.07
1.12
1.07
3.11
9.04
6.01
9.5 9.0 8.5 8.0 7.5 7.0 6.5 6.0 5.5 5.0 4.5 4.0 3.5 3.0 2.5 2.0 1.5 1.0 0.5 0.0 ppm

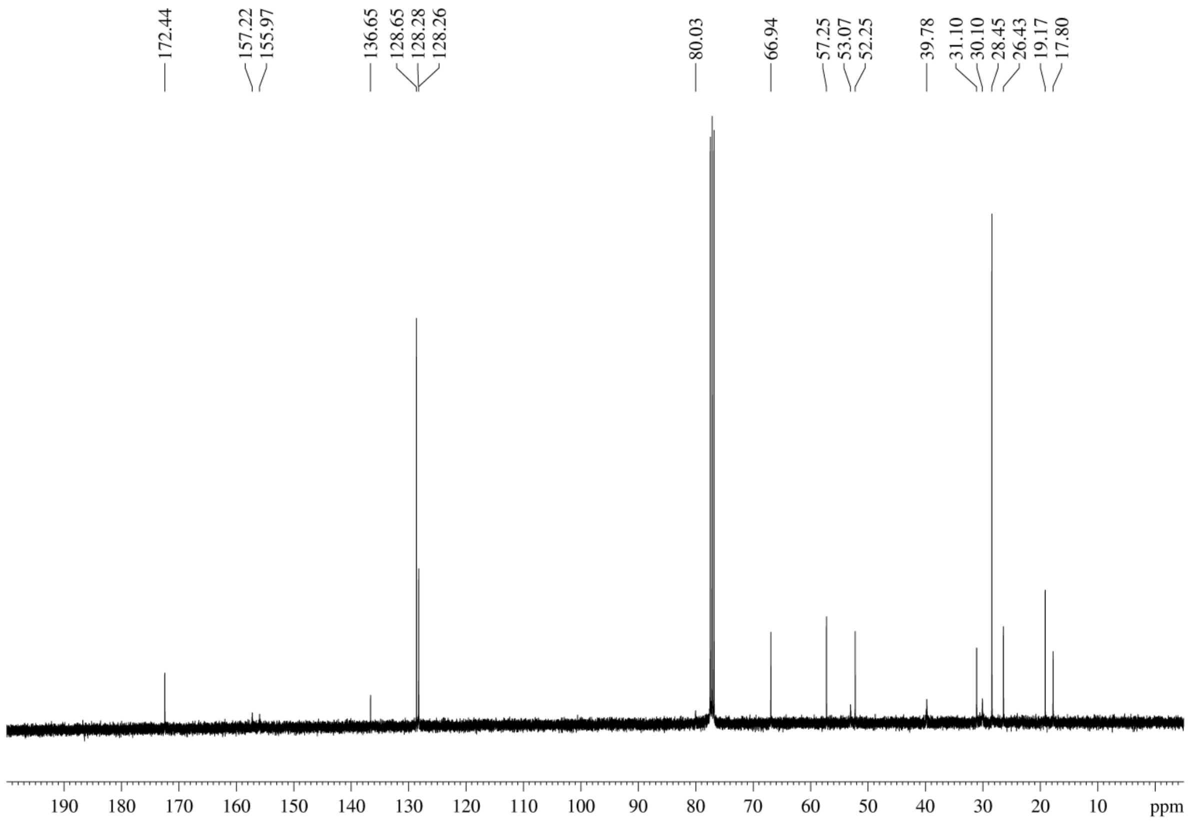
172.44
157.22
155.97
136.65
128.65
128.28
128.26
80.03
66.94
57.25
53.07
52.25
39.78
31.10
30.10
28.45
26.43
19.17
17.80
190 180 170 160 150 140 130 120 110 100 90 80 70 60 50 40 30 20 10 ppm

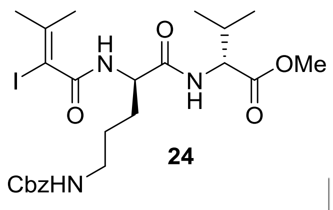
O
H
N
OMe
I
O
N
H
O
CbzHN

24

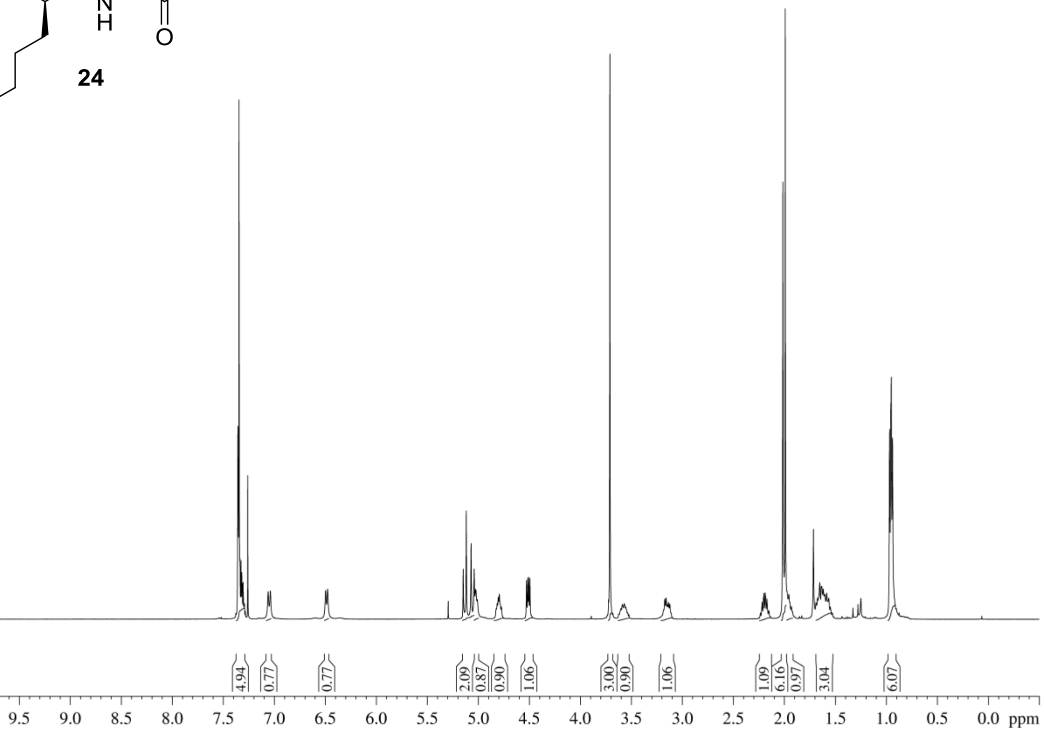
4.94
0.77
0.77
2.09
0.87
0.90
1.06
3.00
0.90
1.06
1.09
6.16
0.97
3.04
6.07
9.5 9.0 8.5 8.0 7.5 7.0 6.5 6.0 5.5 5.0 4.5 4.0 3.5 3.0 2.5 2.0 1.5 1.0 0.5 0.0 ppm

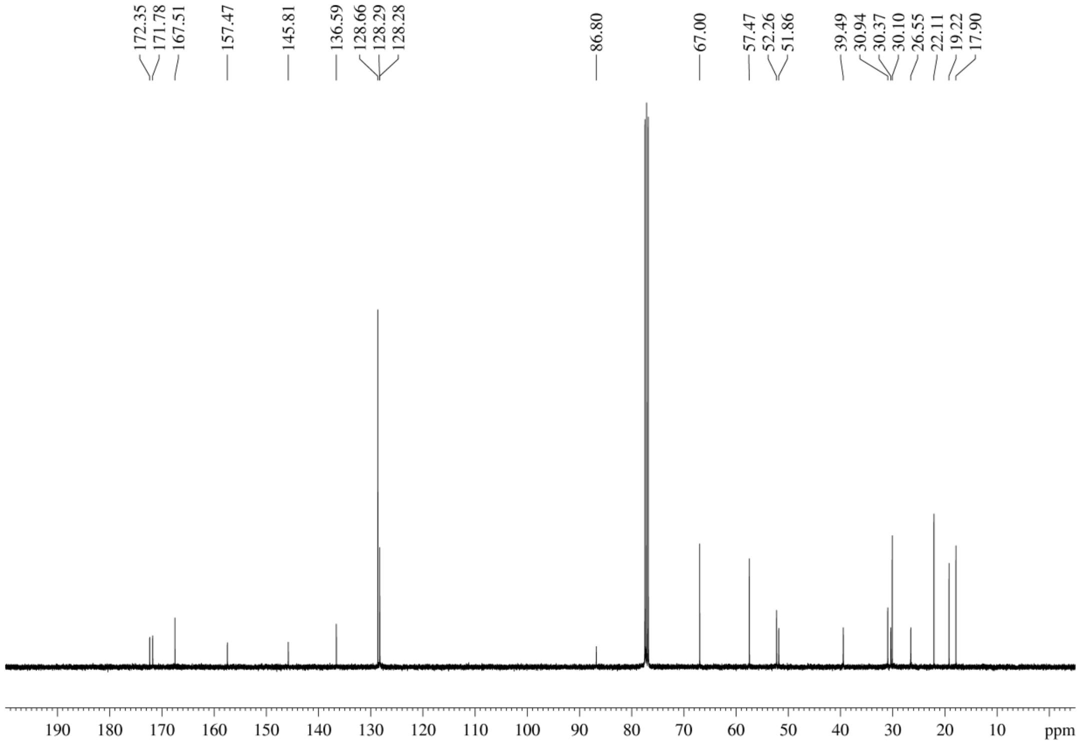
172.35
171.78
167.51
157.47
145.81
136.59
128.66
128.29
128.28
86.80
67.00
57.47
52.26
51.86
39.49
30.94
30.37
30.10
26.55
22.11
19.22
17.90
190 180 170 160 150 140 130 120 110 100 90 80 70 60 50 40 30 20 10 ppm

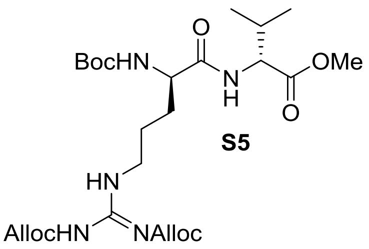
BocHN
O
N
H
OMe
O
HN
AllocHN
NAlloc

S5

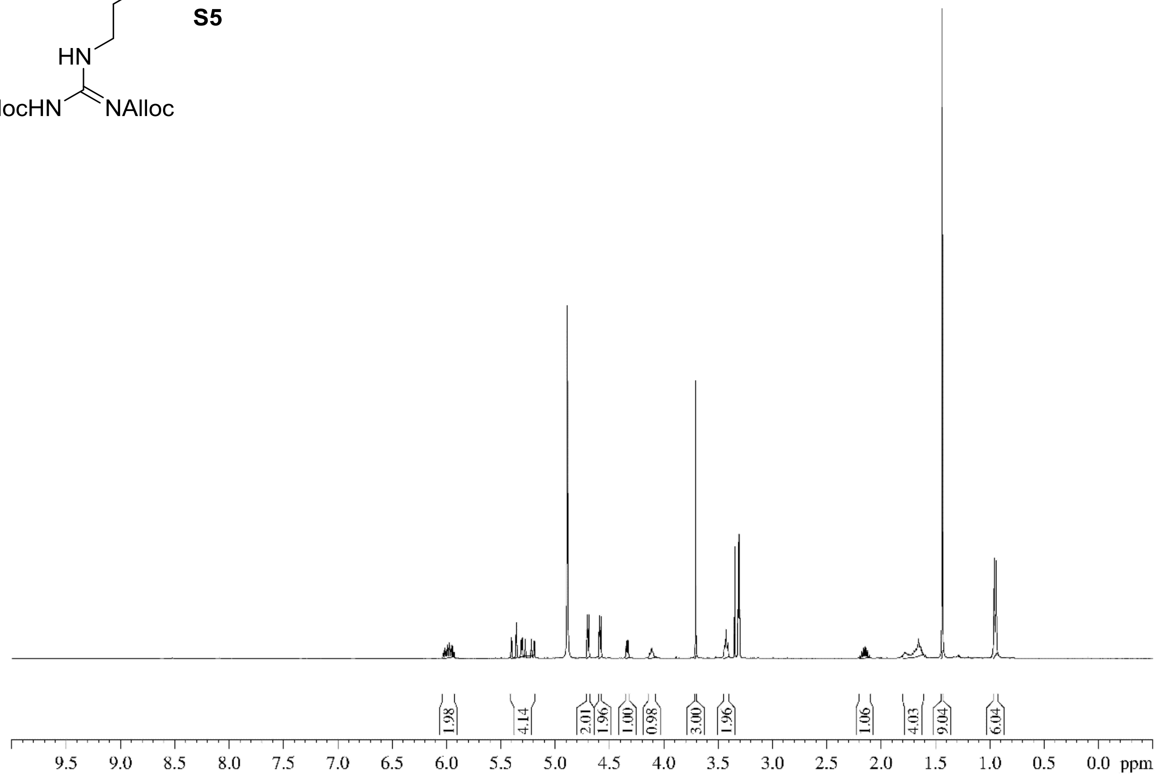
1.98
4.14
2.01
1.96
1.00
0.98
3.00
1.96
1.06
4.03
9.04
6.04
9.5 9.0 8.5 8.0 7.5 7.0 6.5 6.0 5.5 5.0 4.5 4.0 3.5 3.0 2.5 2.0 1.5 1.0 0.5 0.0 ppm

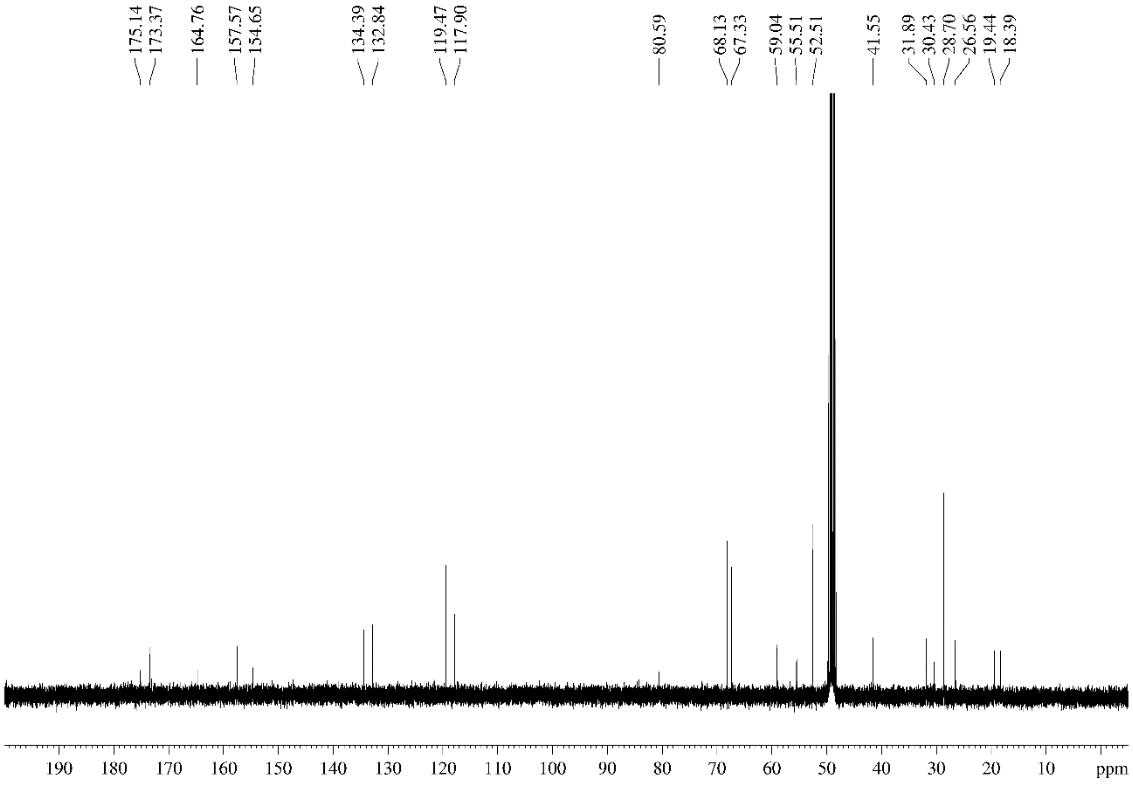
175.14
173.37
164.76
157.57
154.65
134.39
132.84
119.47
117.90
80.59
68.13
67.33
59.04
55.51
52.51
41.55
31.89
30.43
28.70
26.56
19.44
18.39
190 180 170 160 150 140 130 120 110 100 90 80 70 60 50 40 30 20 10 ppm

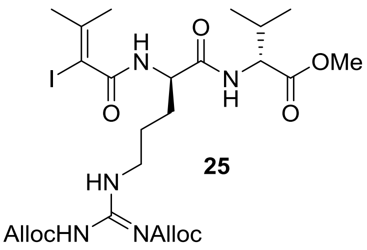

I
H
N
O
O
N
H
OMe
O
HN
AllocHN
NAlloc

25

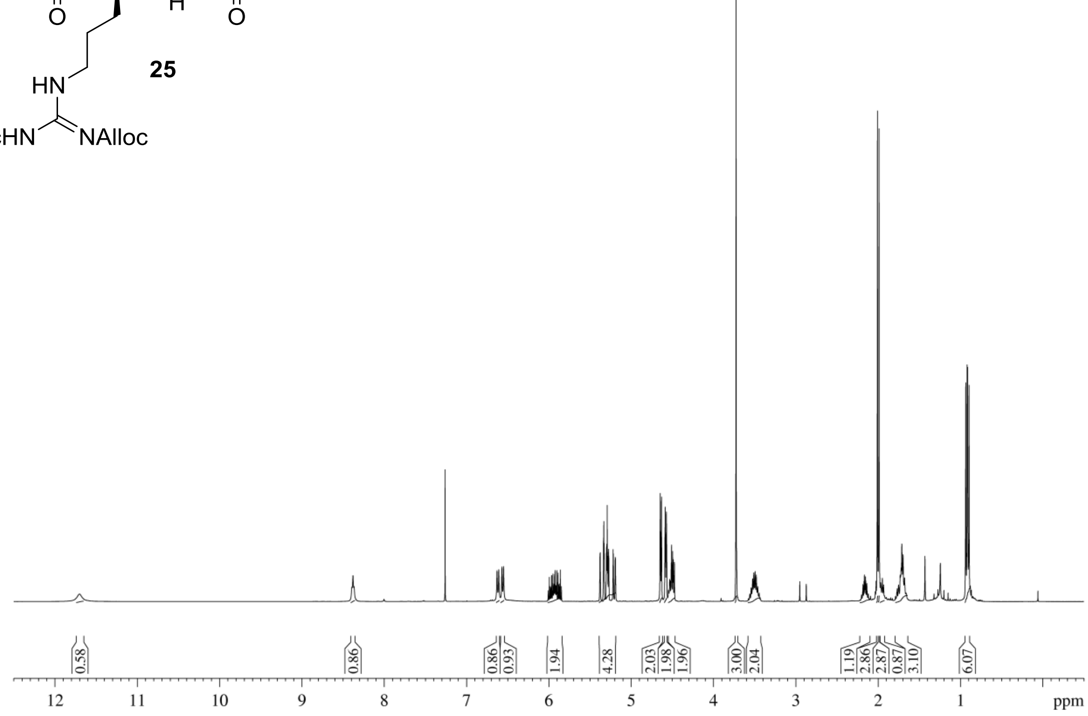

0.58
0.86
0.86
0.93
1.94
4.28
2.03
1.98
1.96
3.00
2.04
1.19
2.86
2.87
0.87
3.10
6.07
12 11 10 9 8 7 6 5 4 3 2 1 ppm

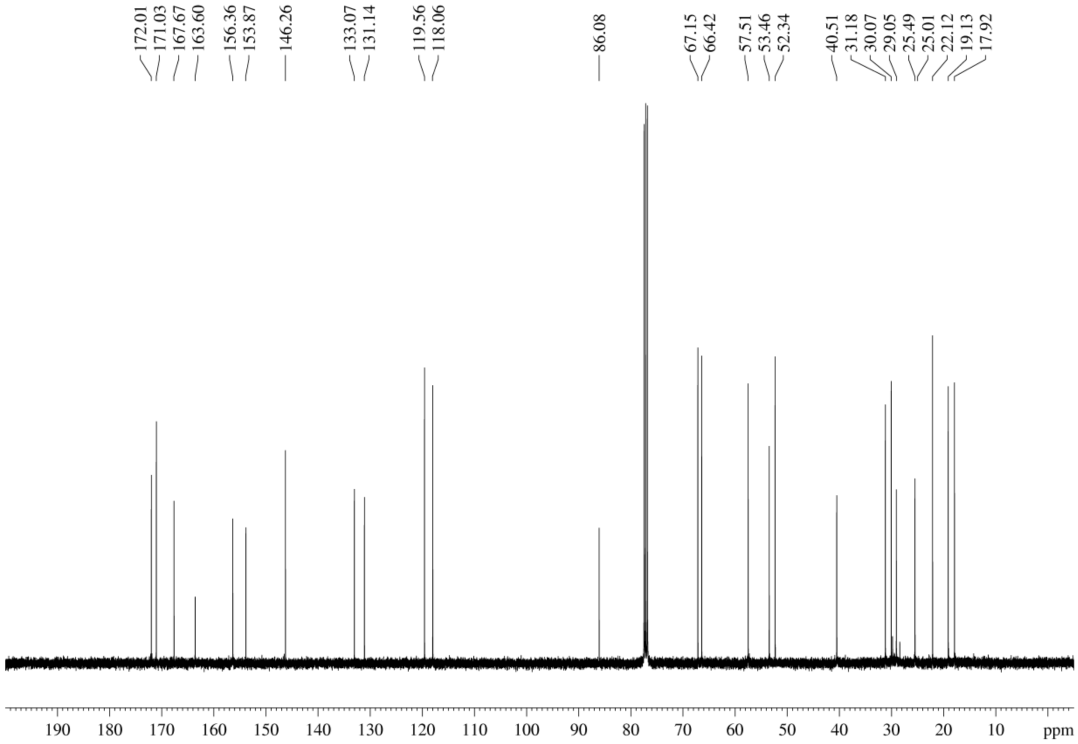

172.01
171.03
167.67
163.60
156.36
153.87
146.26
133.07
131.14
119.56
118.06
86.08
67.15
66.42
57.51
53.46
52.34
40.51
31.18
30.07
29.05
25.49
25.01
22.12
19.13
17.92
190 180 170 160 150 140 130 120 110 100 90 80 70 60 50 40 30 20 10 ppm

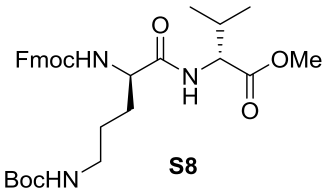
FmocHN
O
N
H
OMe
O
BocHN

S8

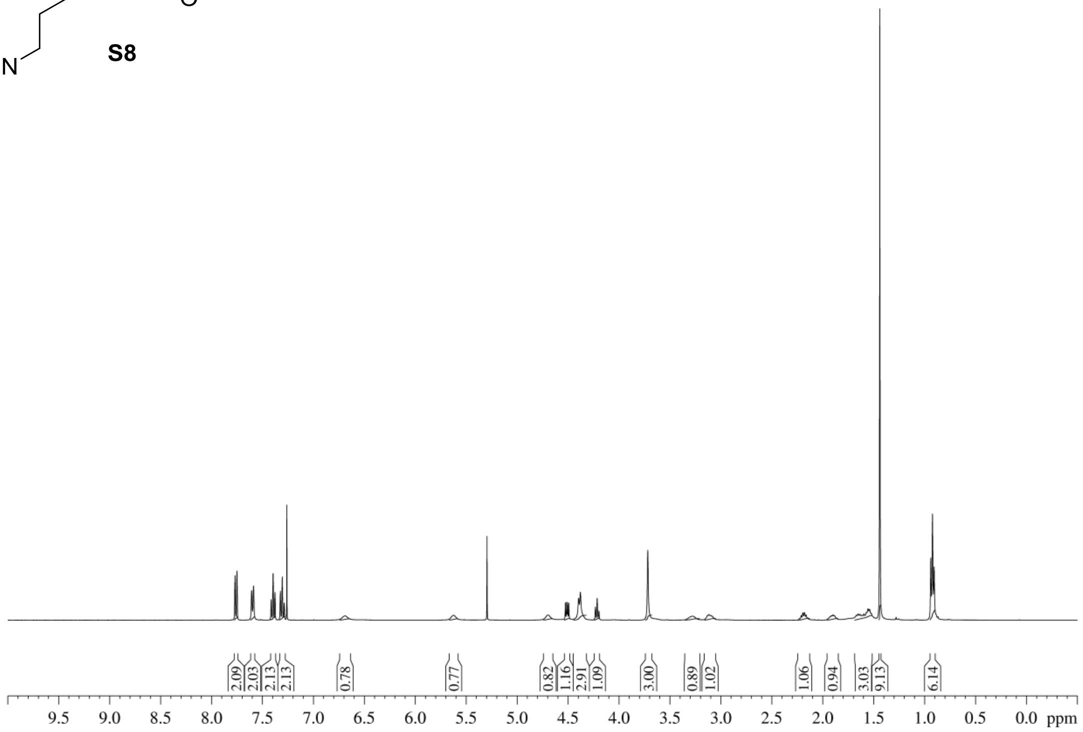
2.09
2.03
2.13
2.13
0.78
0.77
0.82
1.16
2.91
1.09
3.00
0.89
1.02
1.06
0.94
3.03
9.13
6.14
9.5 9.0 8.5 8.0 7.5 7.0 6.5 6.0 5.5 5.0 4.5 4.0 3.5 3.0 2.5 2.0 1.5 1.0 0.5 0.0 ppm

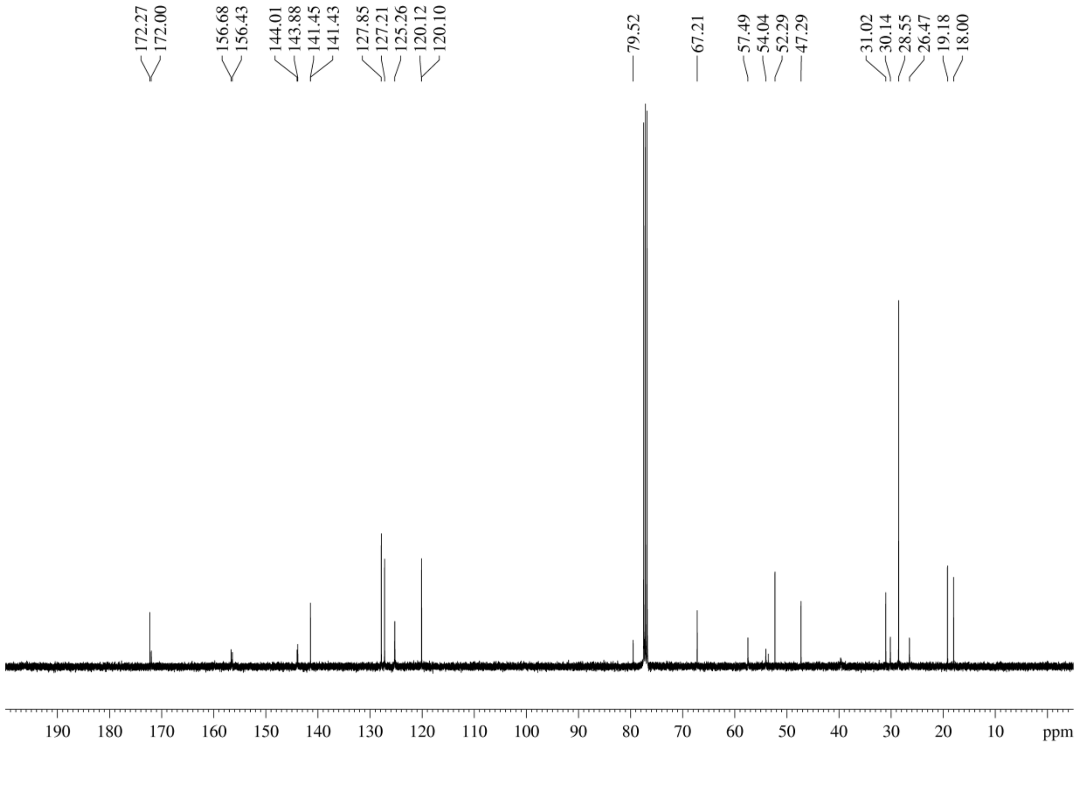
172.27
172.00
156.68
156.43
144.01
143.88
141.45
141.43
127.85
127.21
125.26
120.12
120.10
79.52
67.21
57.49
54.04
52.29
47.29
31.02
30.14
28.55
26.47
19.18
18.00
190 180 170 160 150 140 130 120 110 100 90 80 70 60 50 40 30 20 10 ppm

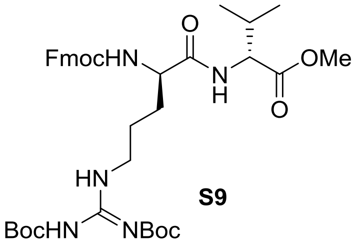

S9

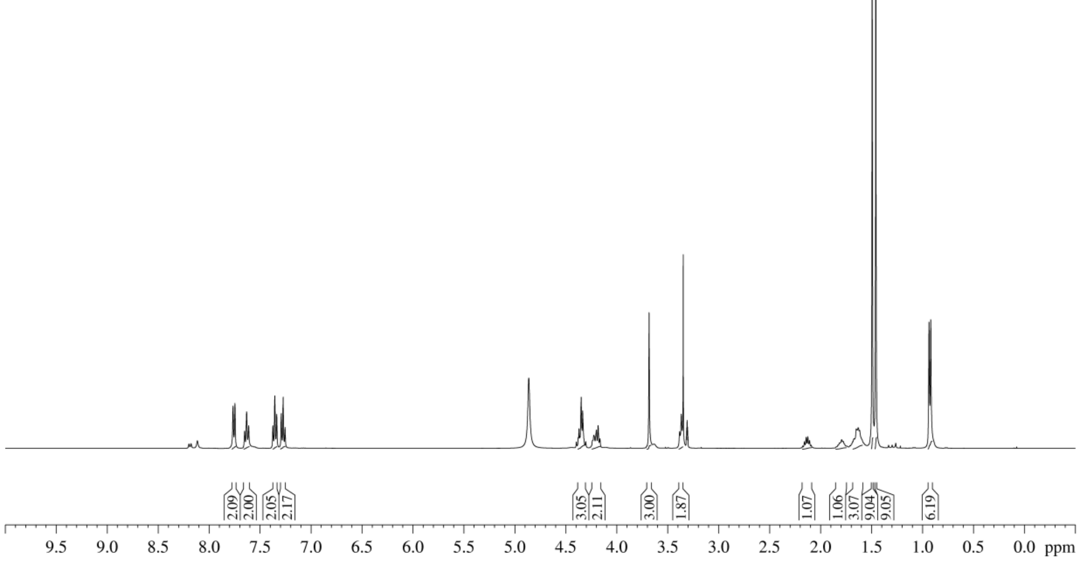

2.09
2.00
2.05
2.17
3.05
2.11
3.00
1.87
1.07
1.06
3.07
9.04
9.05
6.19
9.5 9.0 8.5 8.0 7.5 7.0 6.5 6.0 5.5 5.0 4.5 4.0 3.5 3.0 2.5 2.0 1.5 1.0 0.5 0.0 ppm

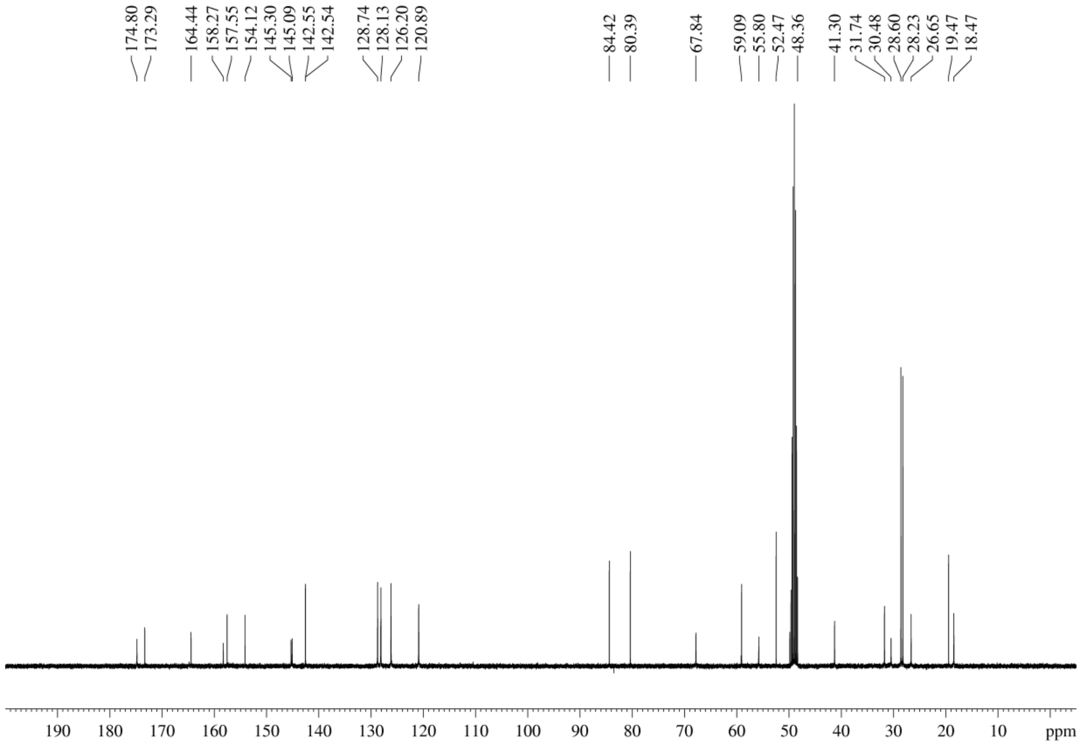

174.80
173.29
164.44
158.27
157.55
154.12
145.30
145.09
142.55
142.54
128.74
128.13
126.20
120.89
84.42
80.39
67.84
59.09
55.80
52.47
48.36
41.30
31.74
30.48
28.60
28.23
26.65
19.47
18.47
190 180 170 160 150 140 130 120 110 100 90 80 70 60 50 40 30 20 10 ppm

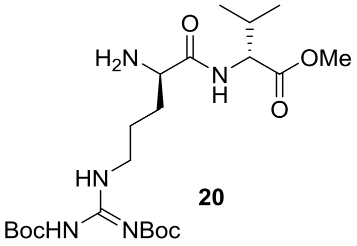

H2N
O
N
H
OMe
O
HN
BocHN
NBoc

20

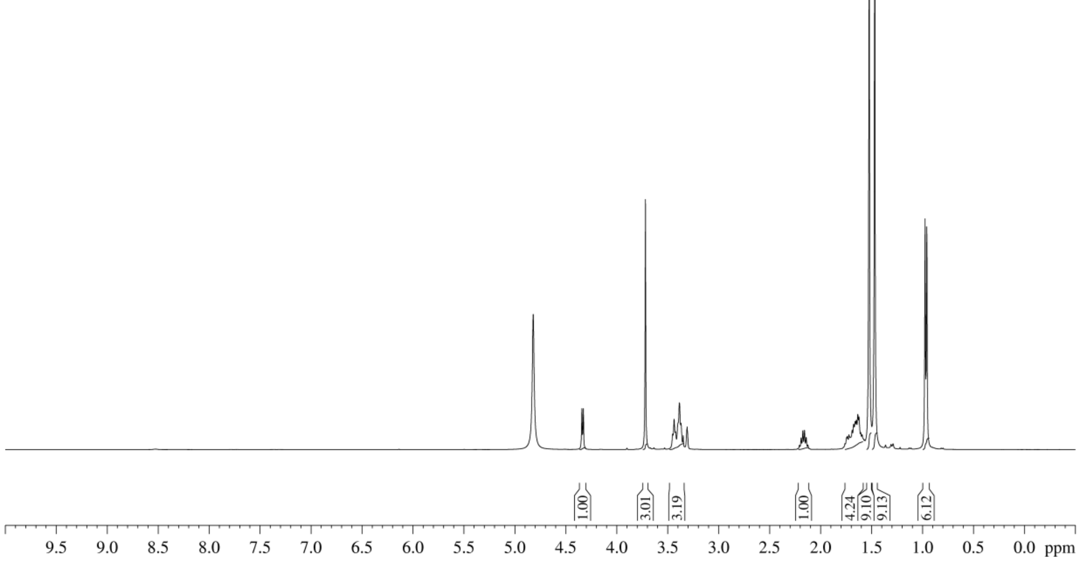

1.00
3.01
3.19
1.00
4.24
9.10
9.13
6.12
9.5 9.0 8.5 8.0 7.5 7.0 6.5 6.0 5.5 5.0 4.5 4.0 3.5 3.0 2.5 2.0 1.5 1.0 0.5 0.0 ppm

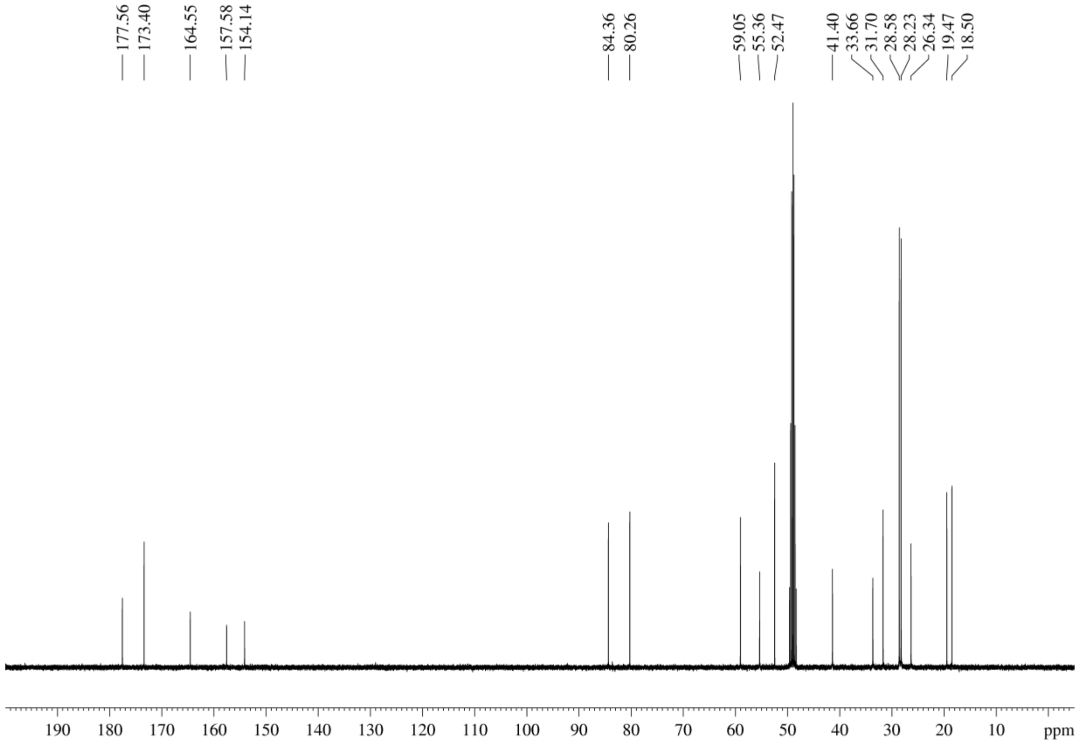

177.56
173.40
164.55
157.58
154.14
84.36
80.26
59.05
55.36
52.47
41.40
33.66
31.70
28.58
28.23
26.34
19.47
18.50
190 180 170 160 150 140 130 120 110 100 90 80 70 60 50 40 30 20 10 ppm

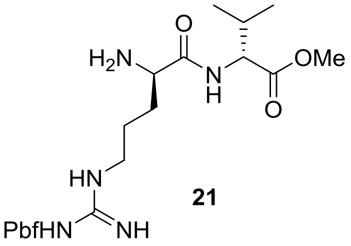
H2N
O
N
H
OMe
O
HN
PbfHN
NH

21

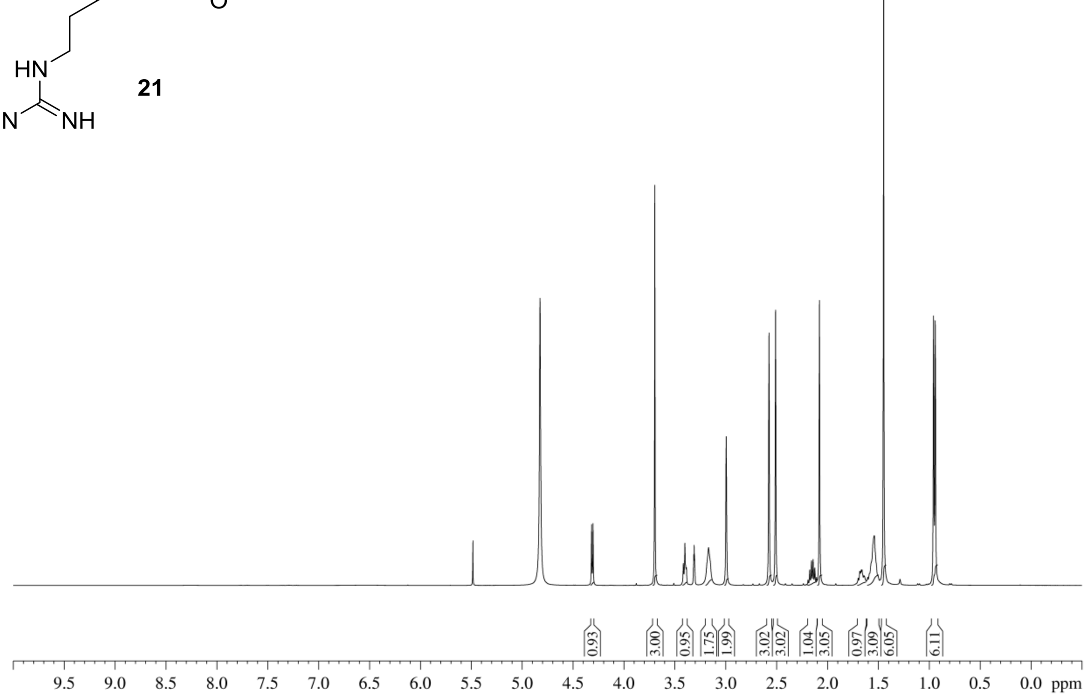
0.93
3.00
0.95
1.75
1.99
3.02
3.02
1.04
3.05
0.97
3.09
6.05
6.11
9.5 9.0 8.5 8.0 7.5 7.0 6.5 6.0 5.5 5.0 4.5 4.0 3.5 3.0 2.5 2.0 1.5 1.0 0.5 0.0 ppm

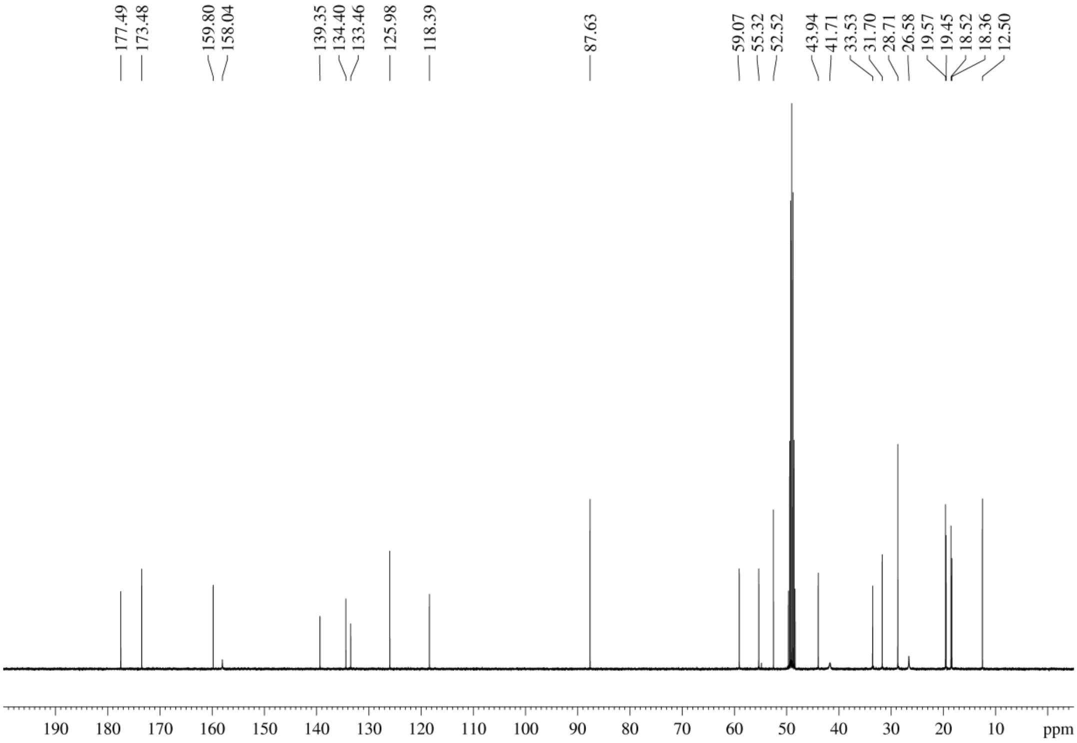
177.49
173.48
159.80
158.04
139.35
134.40
133.46
125.98
118.39
87.63
59.07
55.32
52.52
43.94
41.71
33.53
31.70
28.71
26.58
19.57
19.45
18.52
18.36
12.50
190 180 170 160 150 140 130 120 110 100 90 80 70 60 50 40 30 20 10 ppm

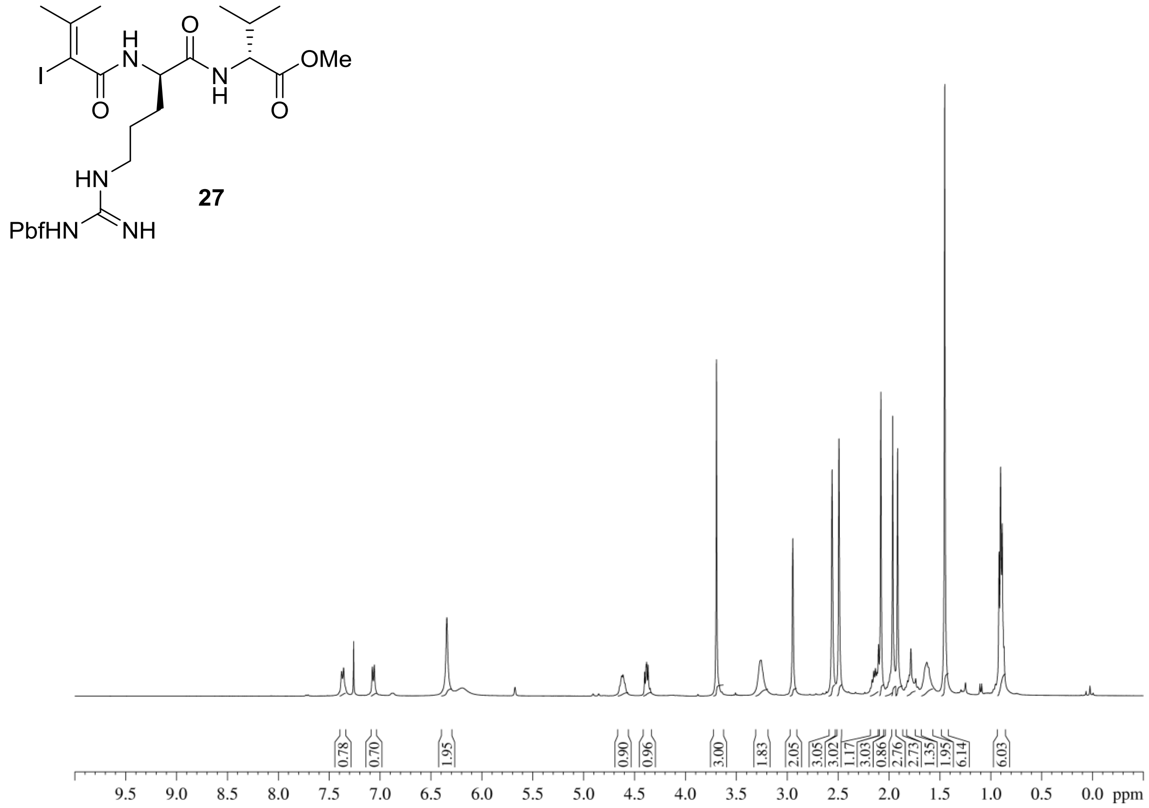

I
H
N
O
OMe
HN
PbfHN
NH
0.78
0.70
1.95
0.90
0.96
3.00
1.83
2.05
3.05
3.02
1.17
3.03
0.86
2.76
2.73
1.35
1.95
6.14
6.03
9.5 9.0 8.5 8.0 7.5 7.0 6.5 6.0 5.5 5.0 4.5 4.0 3.5 3.0 2.5 2.0 1.5 1.0 0.5 0.0 ppm

27

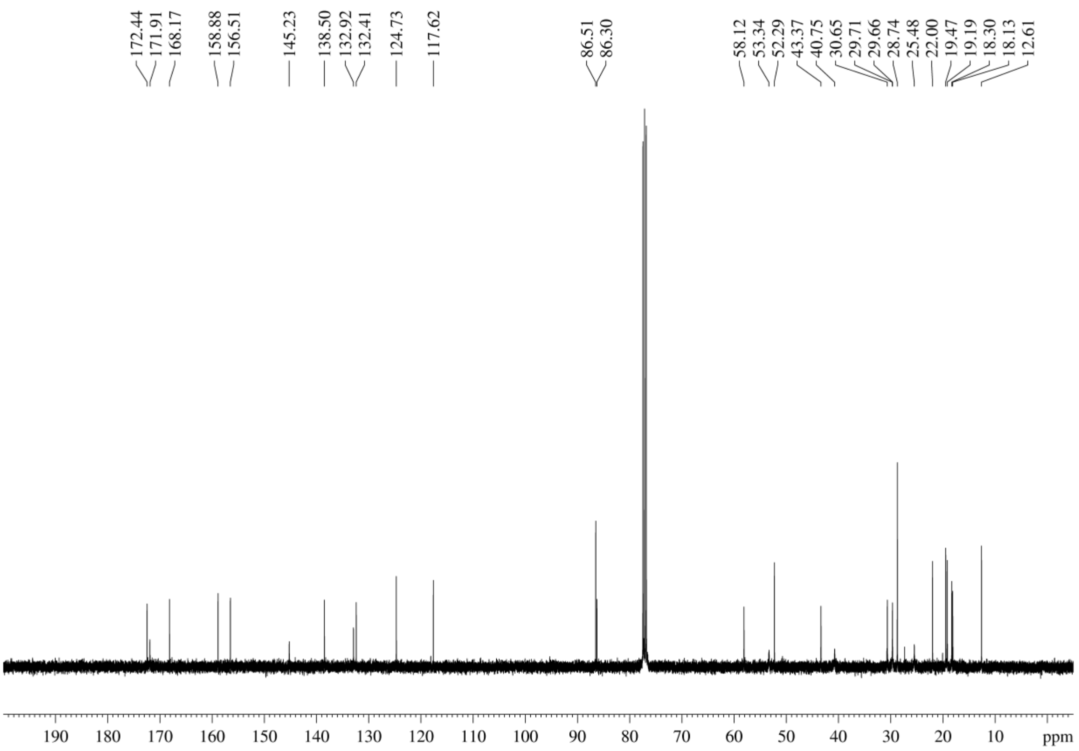

172.44
171.91
168.17
158.88
156.51
145.23
138.50
132.92
132.41
124.73
117.62
86.51
86.30
58.12
53.34
52.29
43.37
40.75
30.65
29.71
29.66
28.74
25.48
22.00
19.47
19.19
18.30
18.13
12.61
190 180 170 160 150 140 130 120 110 100 90 80 70 60 50 40 30 20 10 ppm

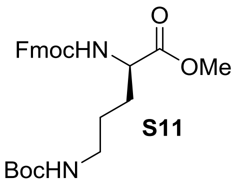
O
FmocHN
OMe
BocHN

S11

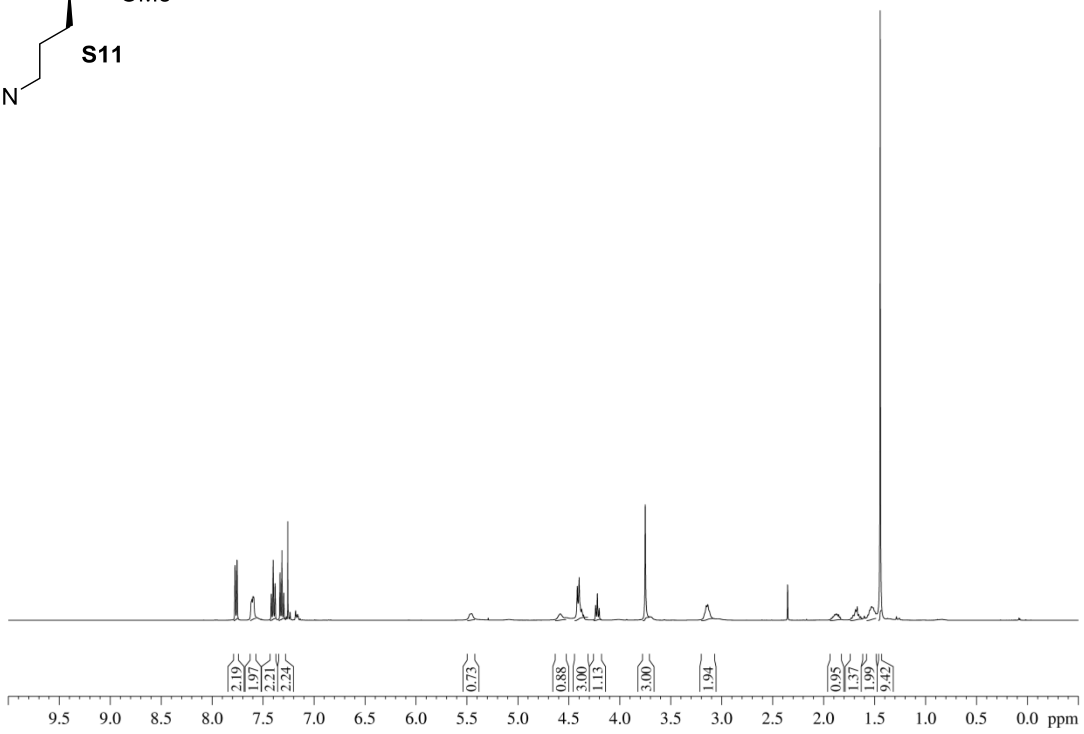
2.19
1.97
2.21
2.24
0.73
0.88
3.00
1.13
3.00
1.94
0.95
1.37
1.99
9.42
9.5 9.0 8.5 8.0 7.5 7.0 6.5 6.0 5.5 5.0 4.5 4.0 3.5 3.0 2.5 2.0 1.5 1.0 0.5 0.0 ppm

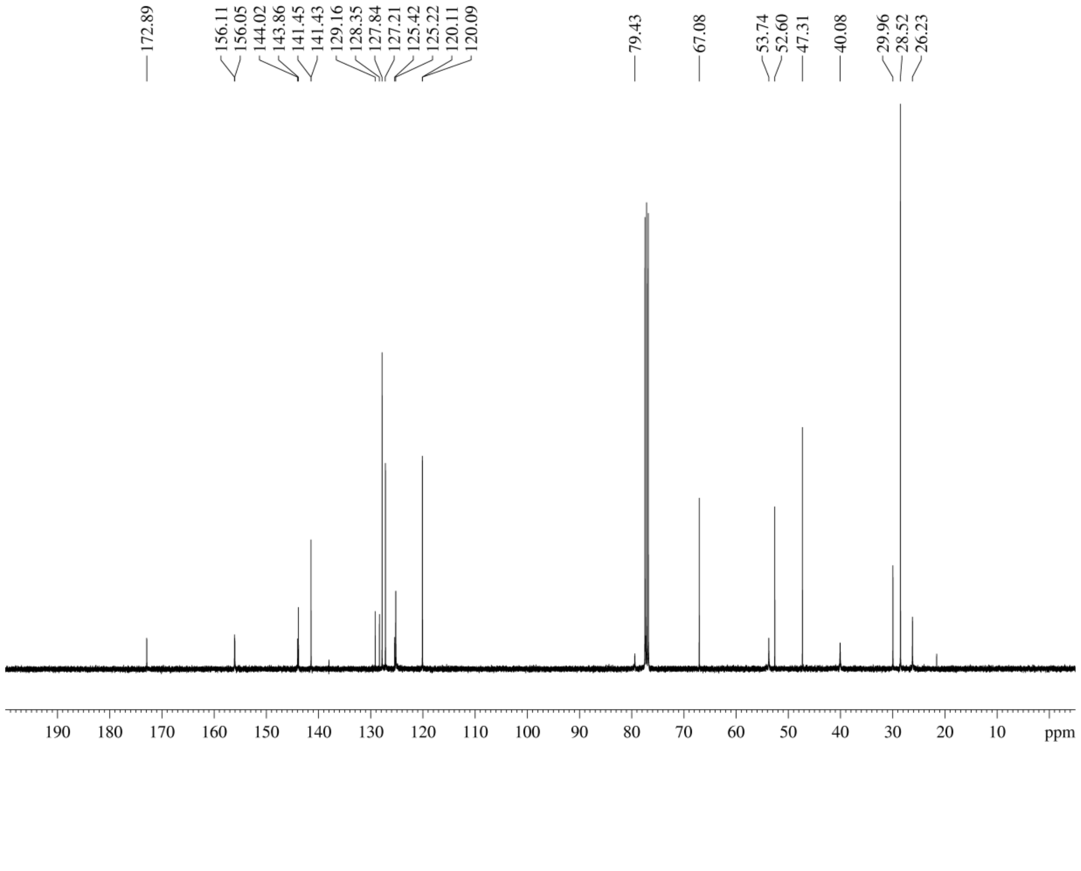
172.89
156.11
156.05
144.02
143.86
141.45
141.43
129.16
128.35
127.84
127.21
125.42
125.22
120.11
120.09
79.43
67.08
53.74
52.60
47.31
40.08
29.96
28.52
26.23
190 180 170 160 150 140 130 120 110 100 90 80 70 60 50 40 30 20 10 ppm

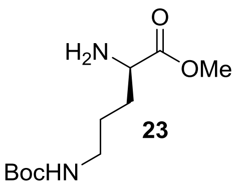
O
H2N
OMe
23
BocHN

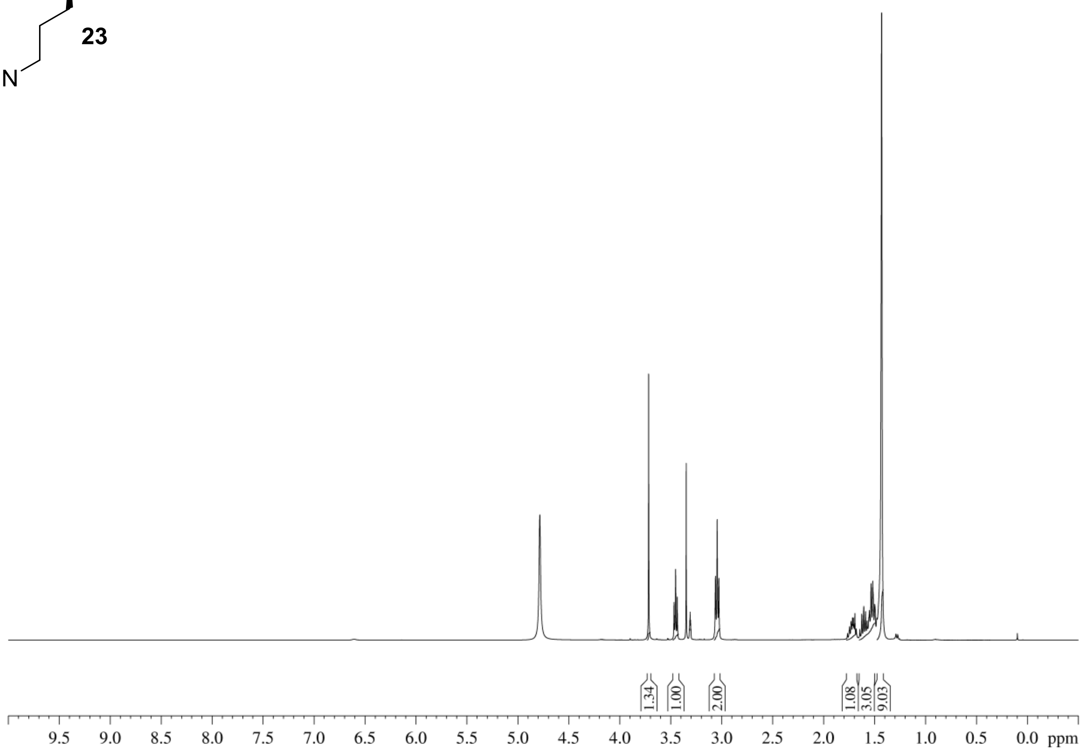
1.34
1.00
2.00
1.08
3.05
9.03
9.5 9.0 8.5 8.0 7.5 7.0 6.5 6.0 5.5 5.0 4.5 4.0 3.5 3.0 2.5 2.0 1.5 1.0 0.5 0.0 ppm

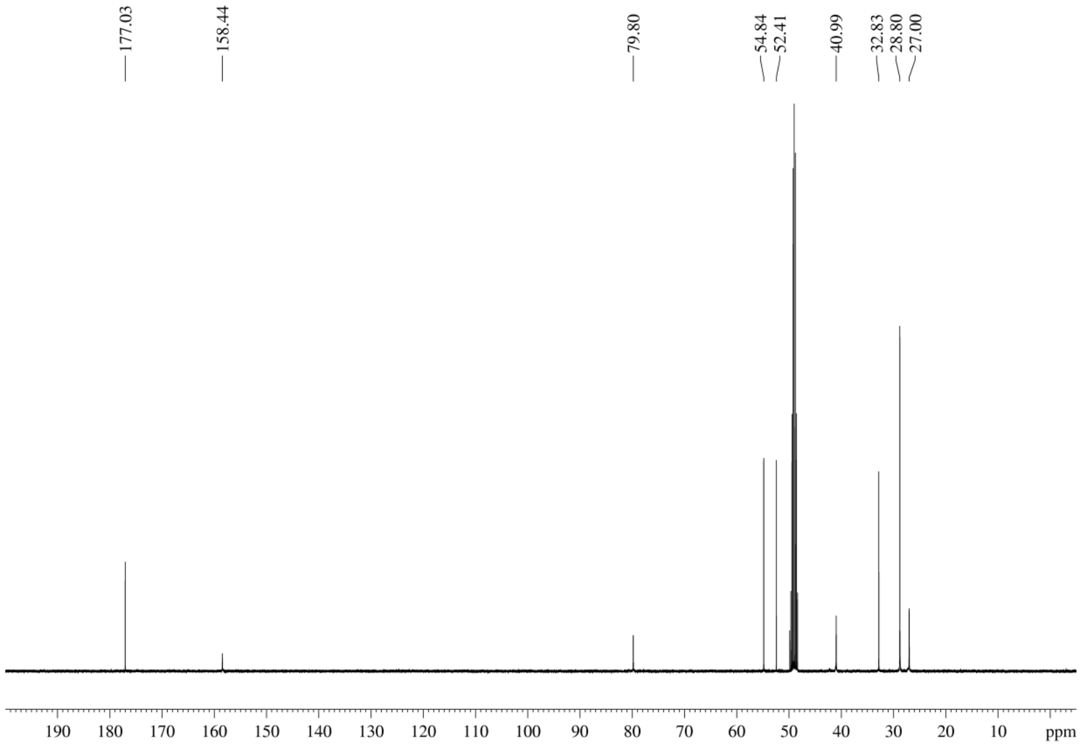
177.03
158.44
79.80
54.84
52.41
40.99
32.83
28.80
27.00
190 180 170 160 150 140 130 120 110 100 90 80 70 60 50 40 30 20 10 ppm

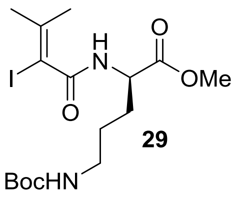
I
H
N
O
OMe
O
29
BocHN

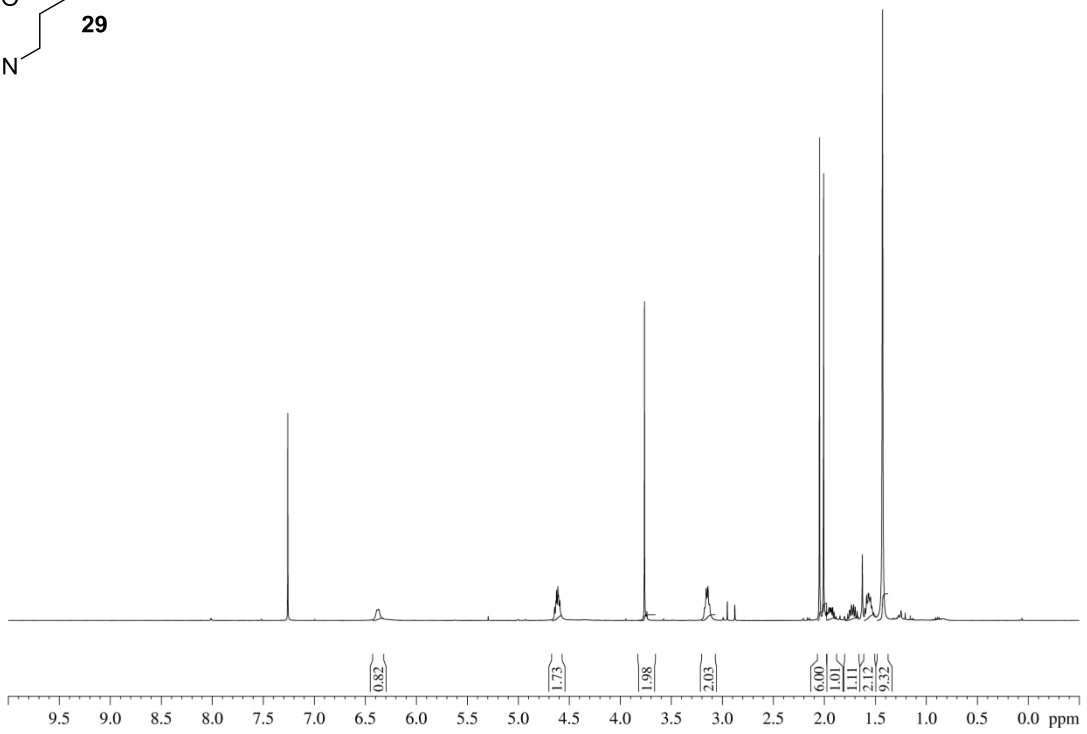
0.82
1.73
1.98
2.03
6.00
1.01
1.11
2.12
9.32
9.5 9.0 8.5 8.0 7.5 7.0 6.5 6.0 5.5 5.0 4.5 4.0 3.5 3.0 2.5 2.0 1.5 1.0 0.5 0.0 ppm

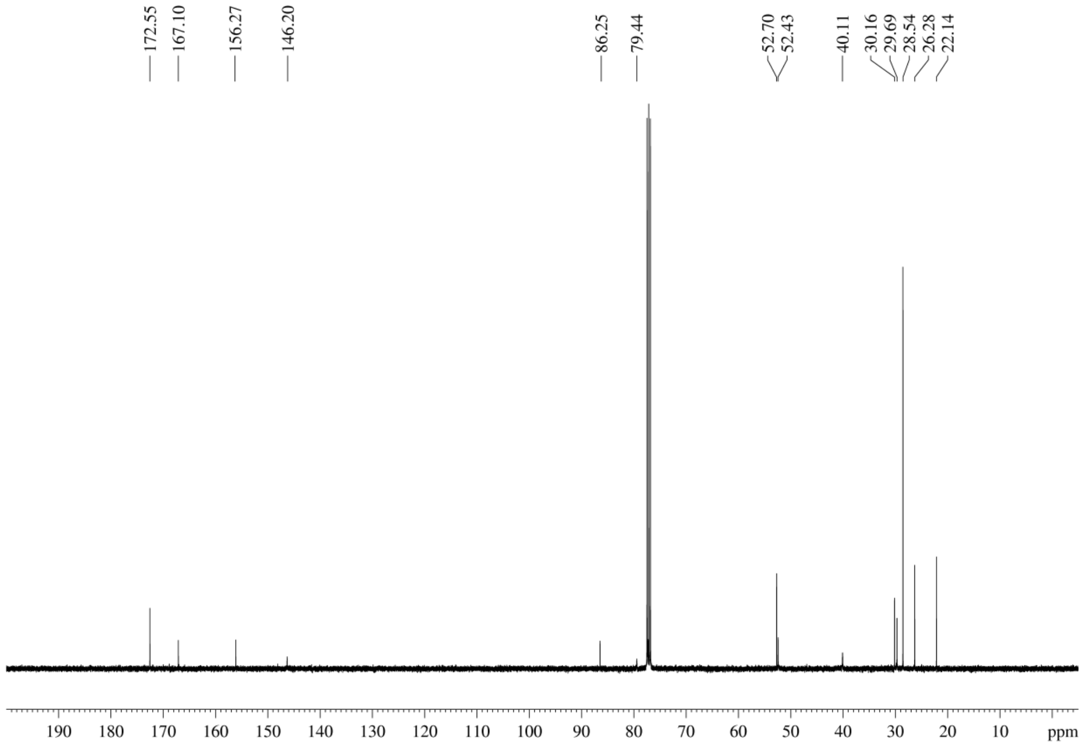
172.55
167.10
156.27
146.20
86.25
79.44
52.70
52.43
40.11
30.16
29.69
28.54
26.28
22.14
190 180 170 160 150 140 130 120 110 100 90 80 70 60 50 40 30 20 10 ppm

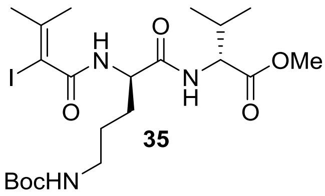
I
OMe
BocHN

35

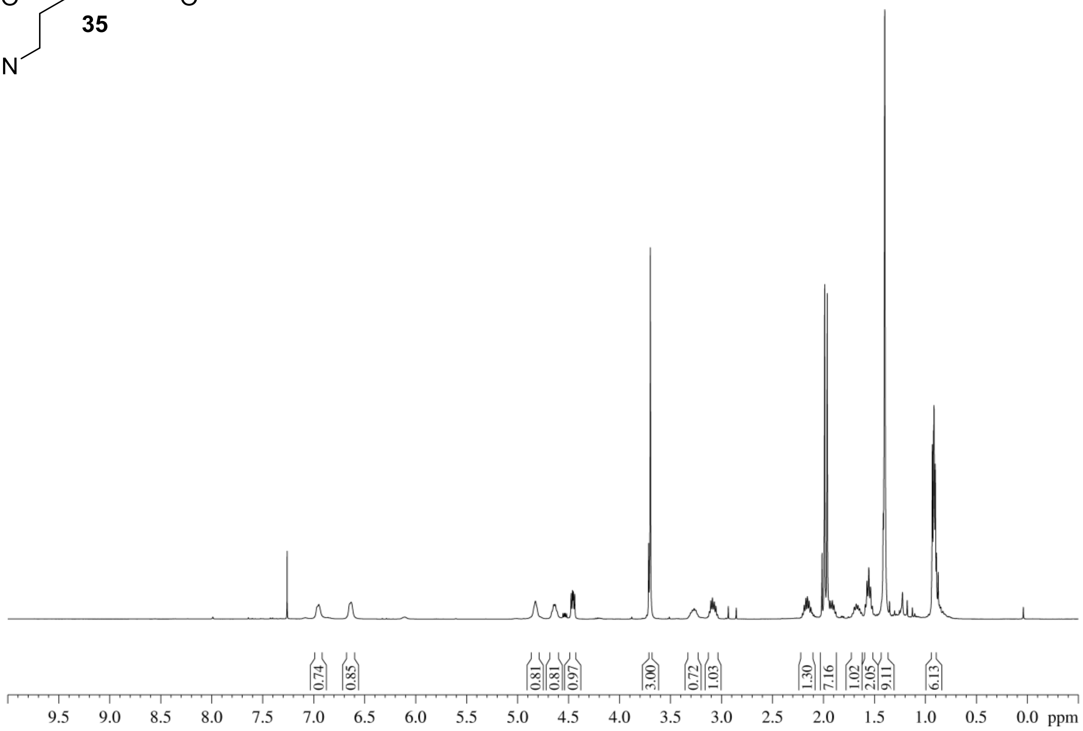
0.74
0.85
0.81
0.81
0.97
3.00
0.72
1.03
1.30
7.16
1.02
2.05
9.11
6.13
ppm

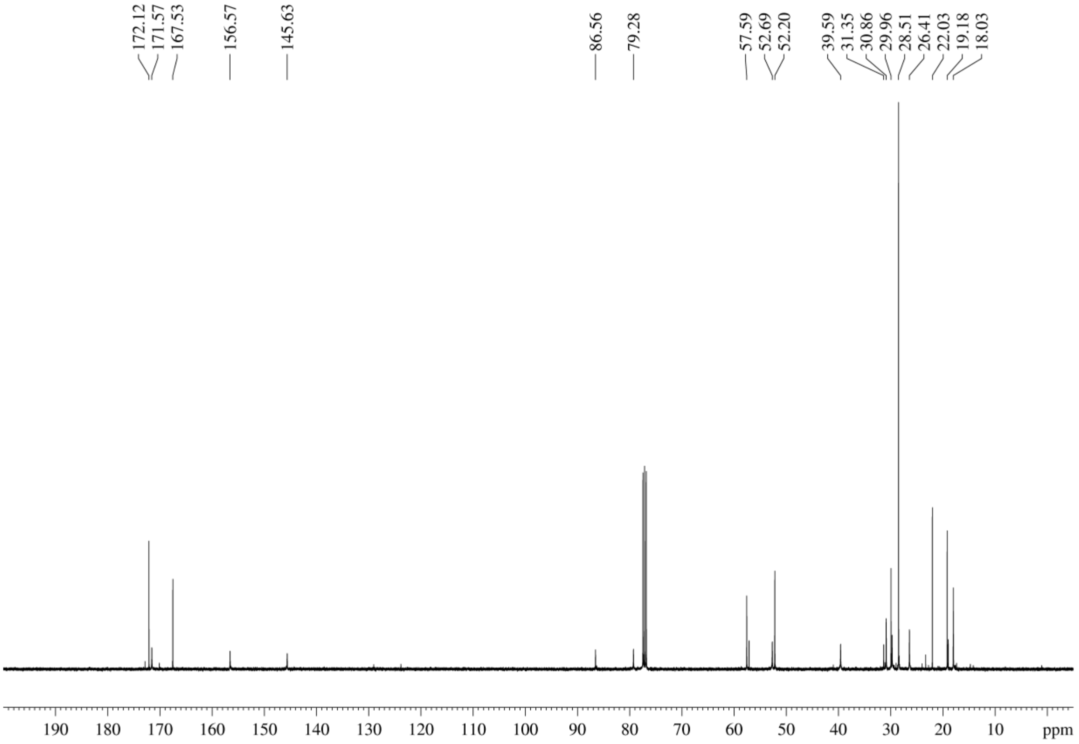
172.12
171.57
167.53
156.57
145.63
86.56
79.28
57.59
52.69
52.20
39.59
31.35
30.86
29.96
28.51
26.41
22.03
19.18
18.03
ppm

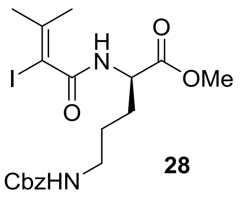

I
H
N
O
OMe
O
CbzHN

28

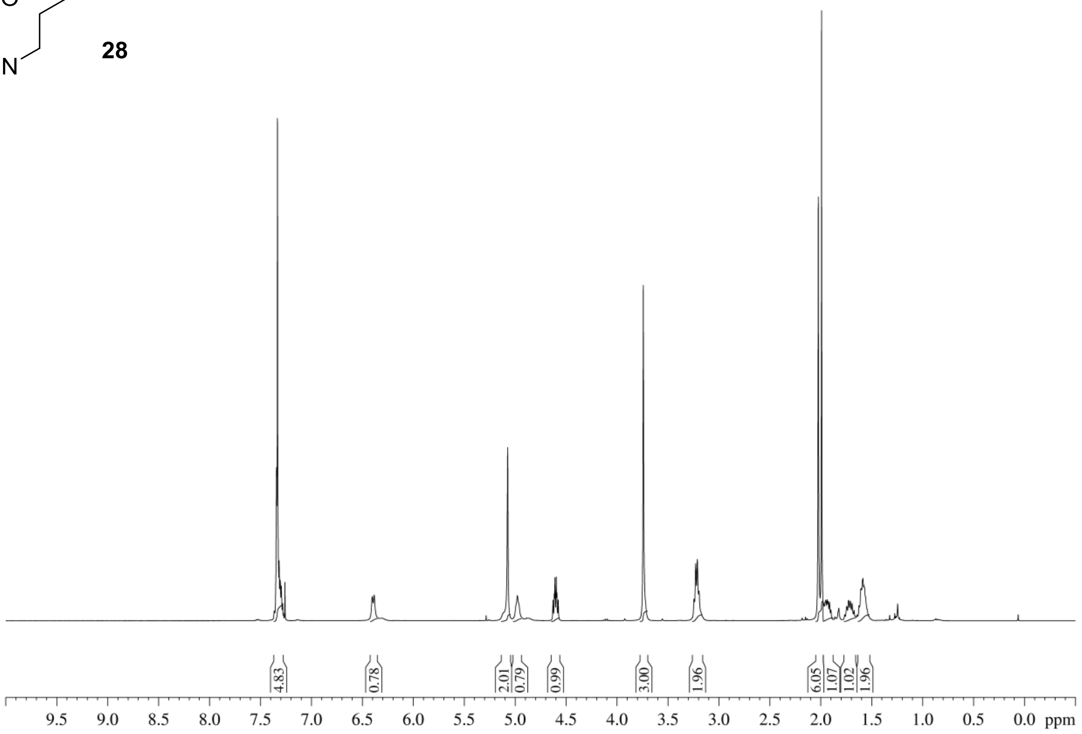

4.83
0.78
2.01
0.79
0.99
3.00
1.96
6.05
1.07
1.02
1.96
9.5 9.0 8.5 8.0 7.5 7.0 6.5 6.0 5.5 5.0 4.5 4.0 3.5 3.0 2.5 2.0 1.5 1.0 0.5 0.0 ppm

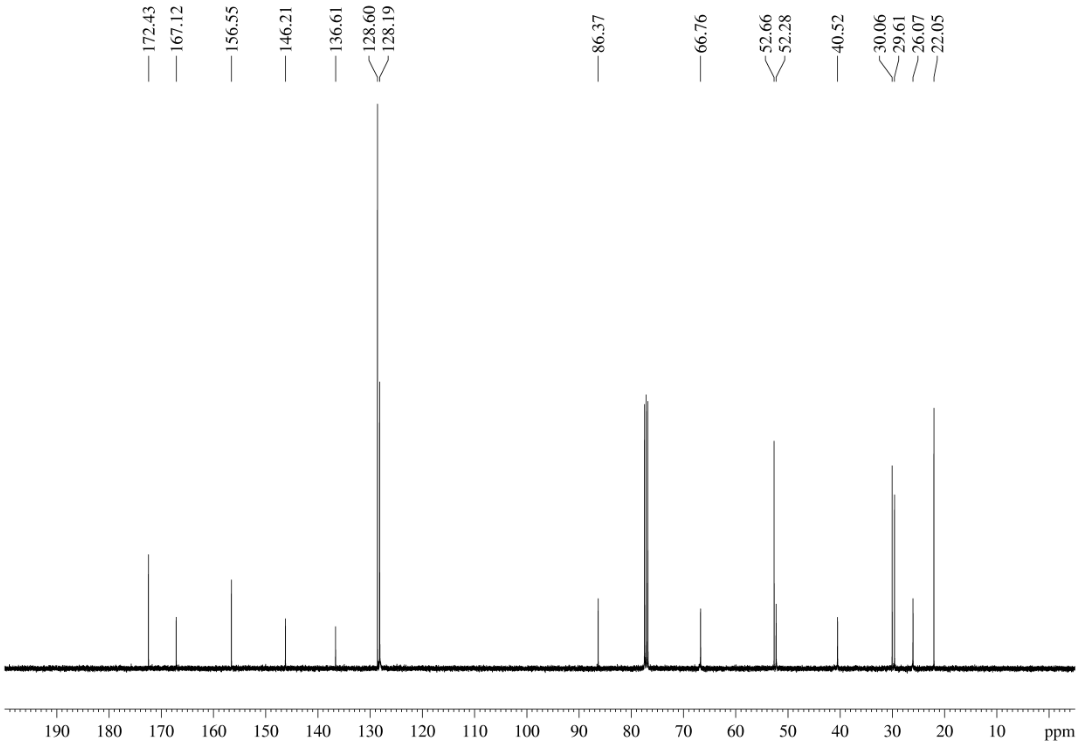

172.43
167.12
156.55
146.21
136.61
128.60
128.19
86.37
66.76
52.66
52.28
40.52
30.06
29.61
26.07
22.05
190 180 170 160 150 140 130 120 110 100 90 80 70 60 50 40 30 20 10 ppm

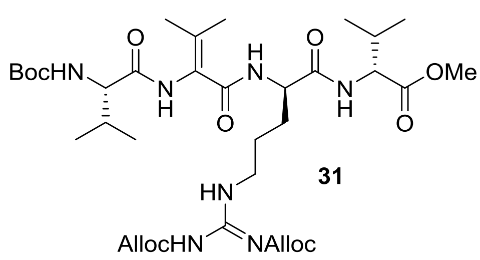
BocHN
OMe
AllocHN
NAlloc
31

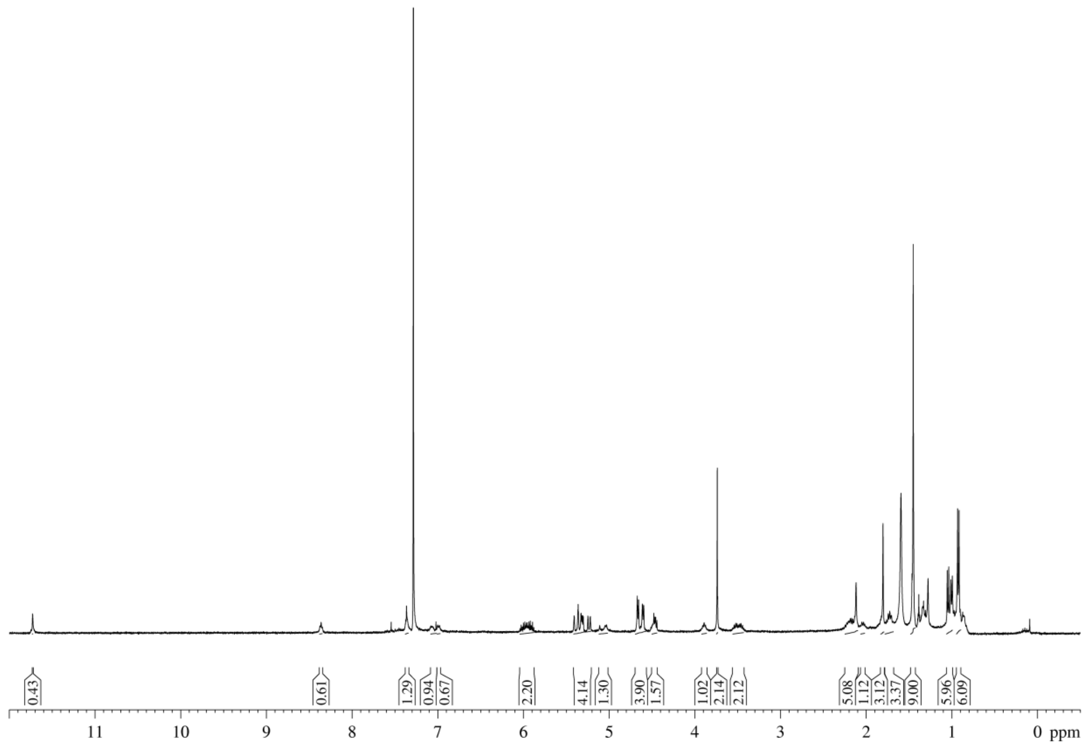
0.43
0.61
1.29
0.94
0.67
2.20
4.14
1.30
3.90
1.57
1.02
2.14
2.12
5.08
1.12
3.12
3.37
9.00
5.96
6.09
11
10
9
8
7
6
5
4
3
2
1
0 ppm

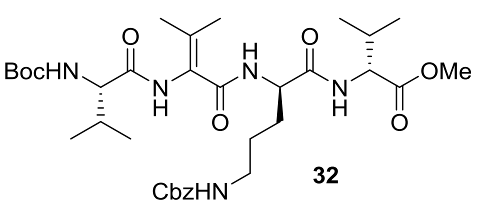
BocHN
OMe
CbzHN

32

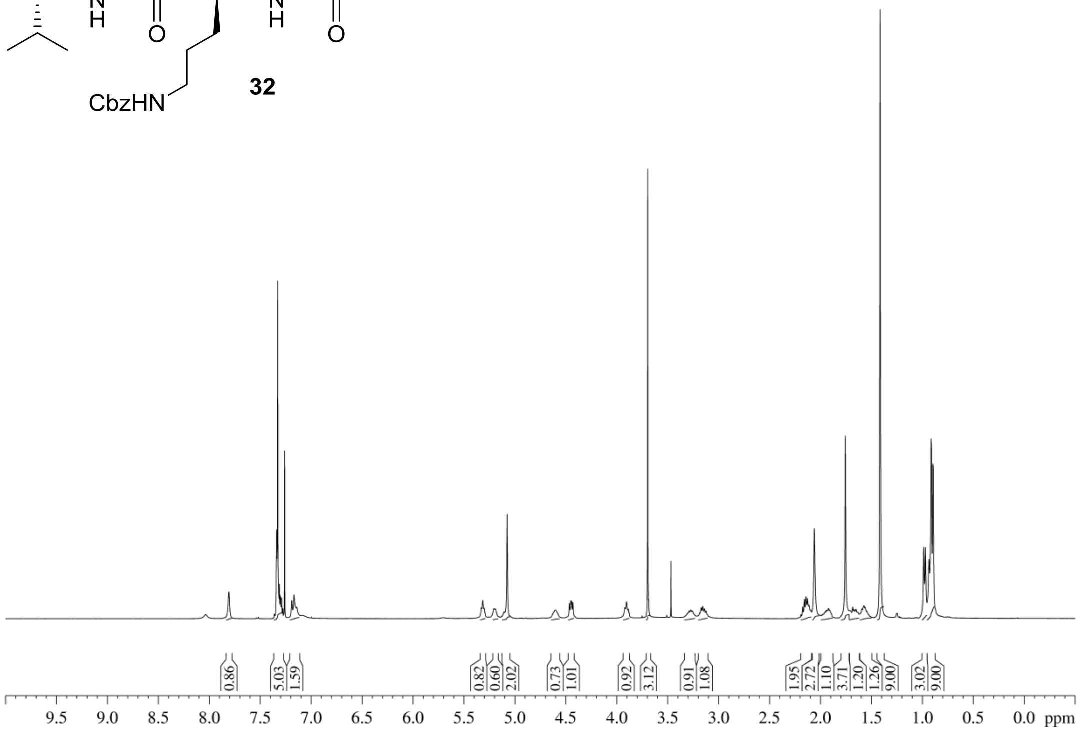
0.86
5.03
1.59
0.82
0.60
2.02
0.73
1.01
0.92
3.12
0.91
1.08
1.95
2.72
1.10
3.71
1.20
1.26
9.00
3.02
9.00
ppm

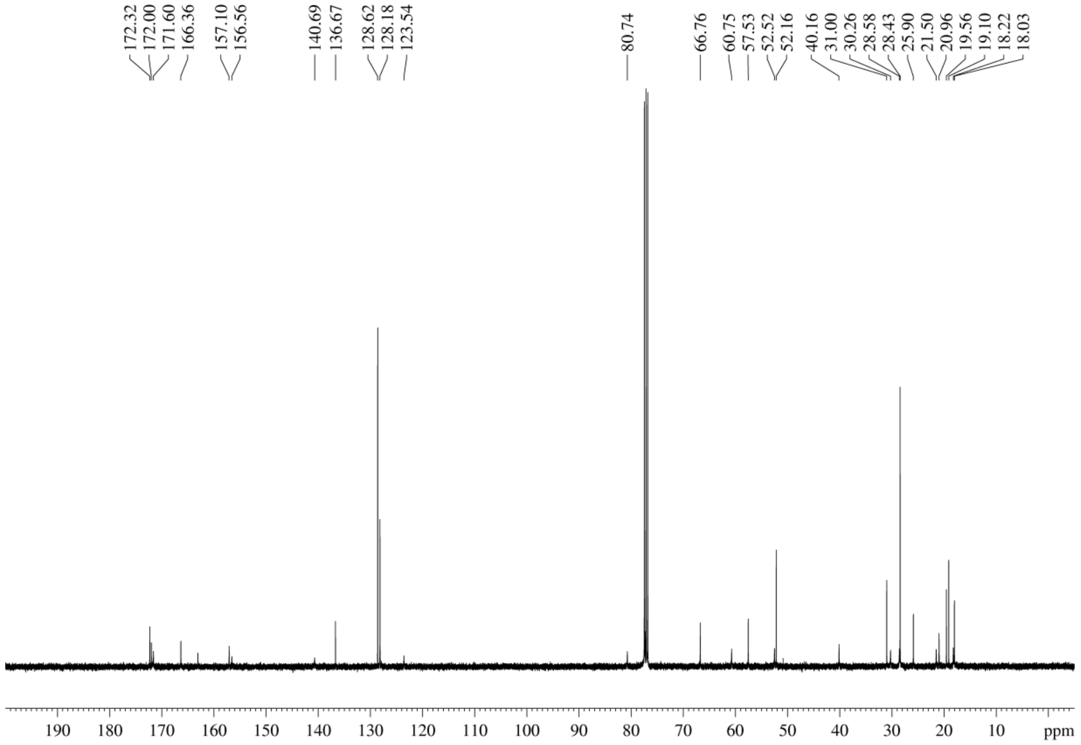
172.32
172.00
171.60
166.36
157.10
156.56
140.69
136.67
128.62
128.18
123.54
80.74
66.76
60.75
57.53
52.52
52.16
40.16
31.00
30.26
28.58
28.43
25.90
21.50
20.96
19.56
19.10
18.22
18.03
ppm

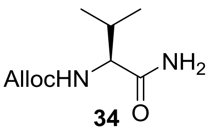
AllocHN
NH2
O
34

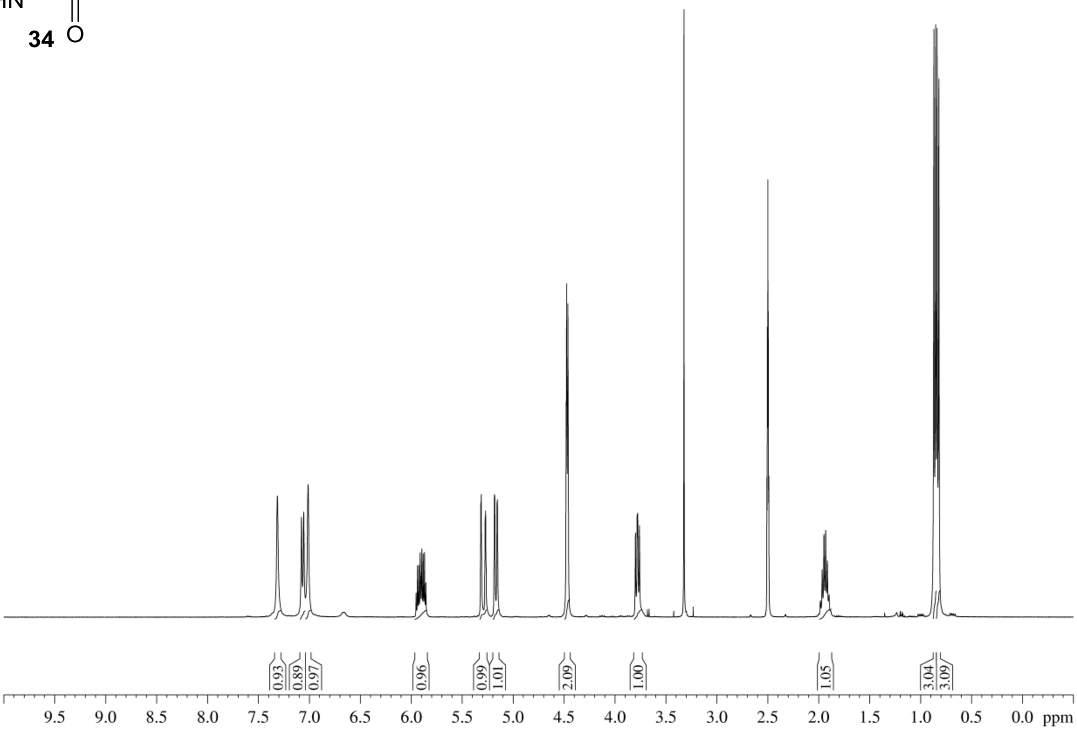
0.93 0.89 0.97 0.96 0.99 1.01 2.09 1.00 1.05 3.04 3.09
9.5 9.0 8.5 8.0 7.5 7.0 6.5 6.0 5.5 5.0 4.5 4.0 3.5 3.0 2.5 2.0 1.5 1.0 0.5 0.0 ppm

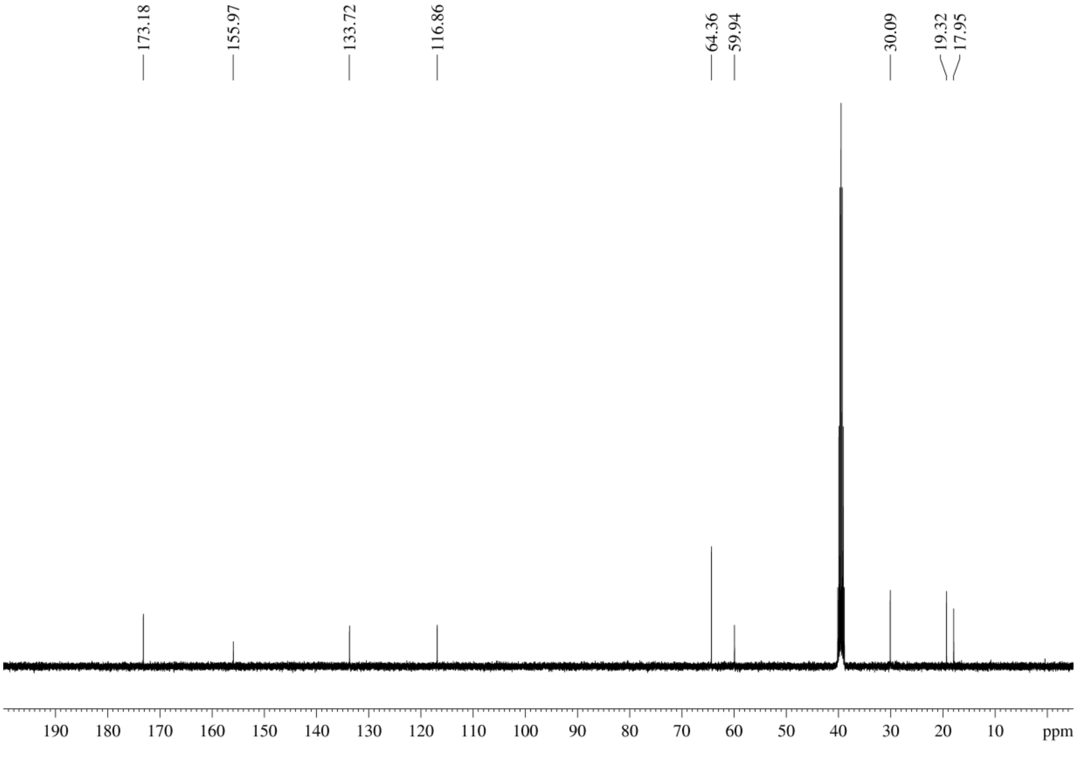
173.18
155.97
133.72
116.86
64.36
59.94
30.09
19.32
17.95
190 180 170 160 150 140 130 120 110 100 90 80 70 60 50 40 30 20 10 ppm

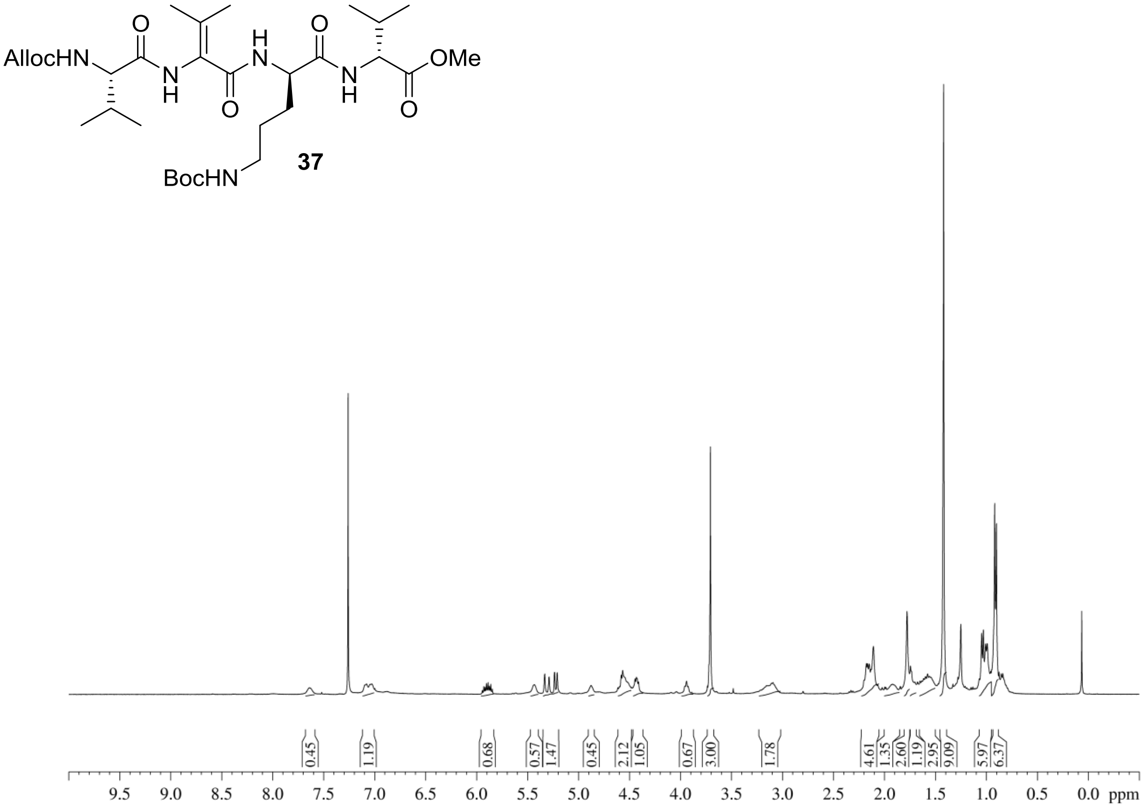
AllocHN
BocHN
OMe
37
ppm
9.5
9.0
8.5
8.0
7.5
7.0
6.5
6.0
5.5
5.0
4.5
4.0
3.5
3.0
2.5
2.0
1.5
1.0
0.5
0.0

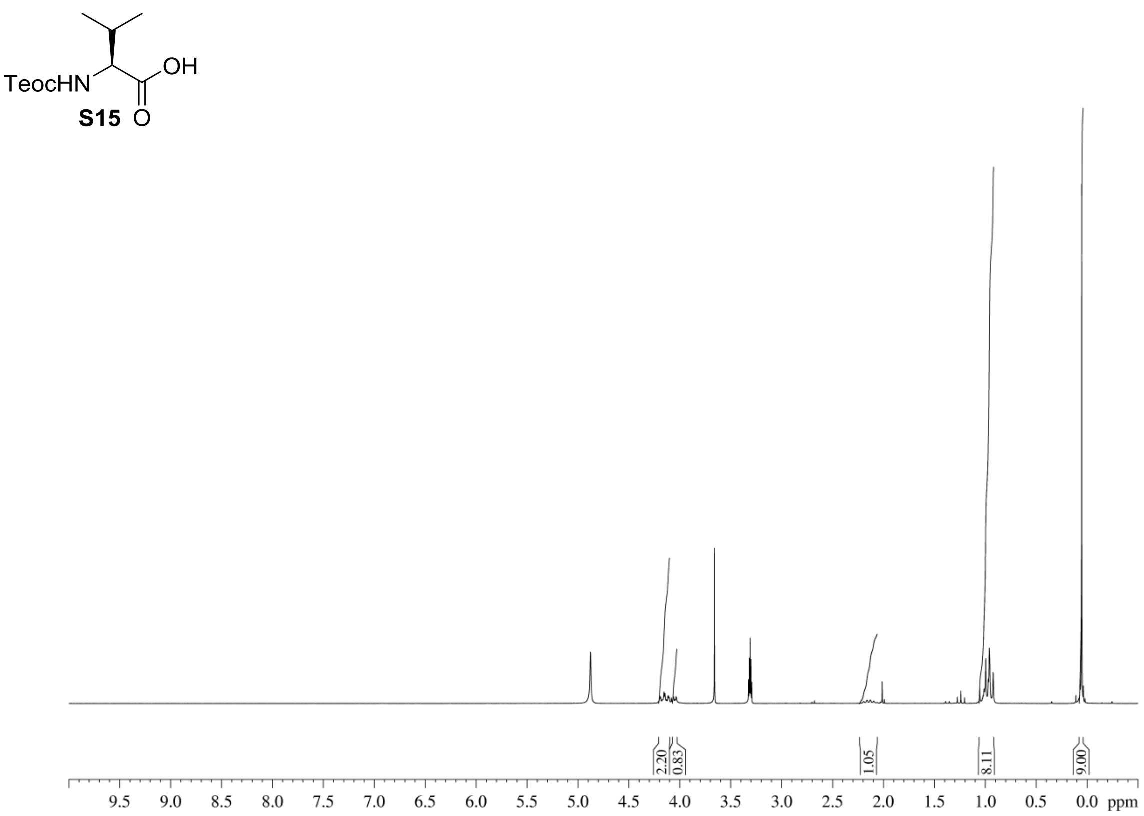
TeocHN
OH
O
S15
2.20
0.83
1.05
8.11
9.00
9.5 9.0 8.5 8.0 7.5 7.0 6.5 6.0 5.5 5.0 4.5 4.0 3.5 3.0 2.5 2.0 1.5 1.0 0.5 0.0 ppm

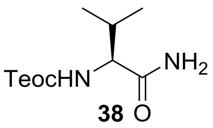
TeocHN
NH2
O

38

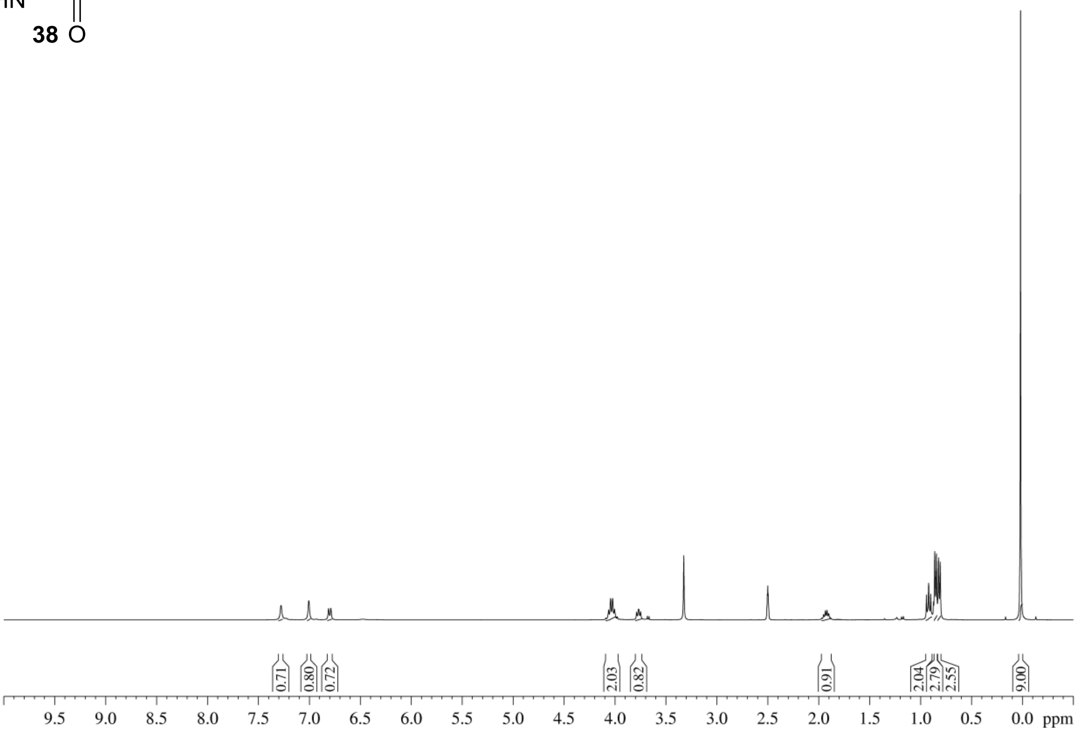
0.71
0.80
0.72
2.03
0.82
0.91
2.04
2.79
2.55
9.00
9.5
9.0
8.5
8.0
7.5
7.0
6.5
6.0
5.5
5.0
4.5
4.0
3.5
3.0
2.5
2.0
1.5
1.0
0.5
0.0 ppm

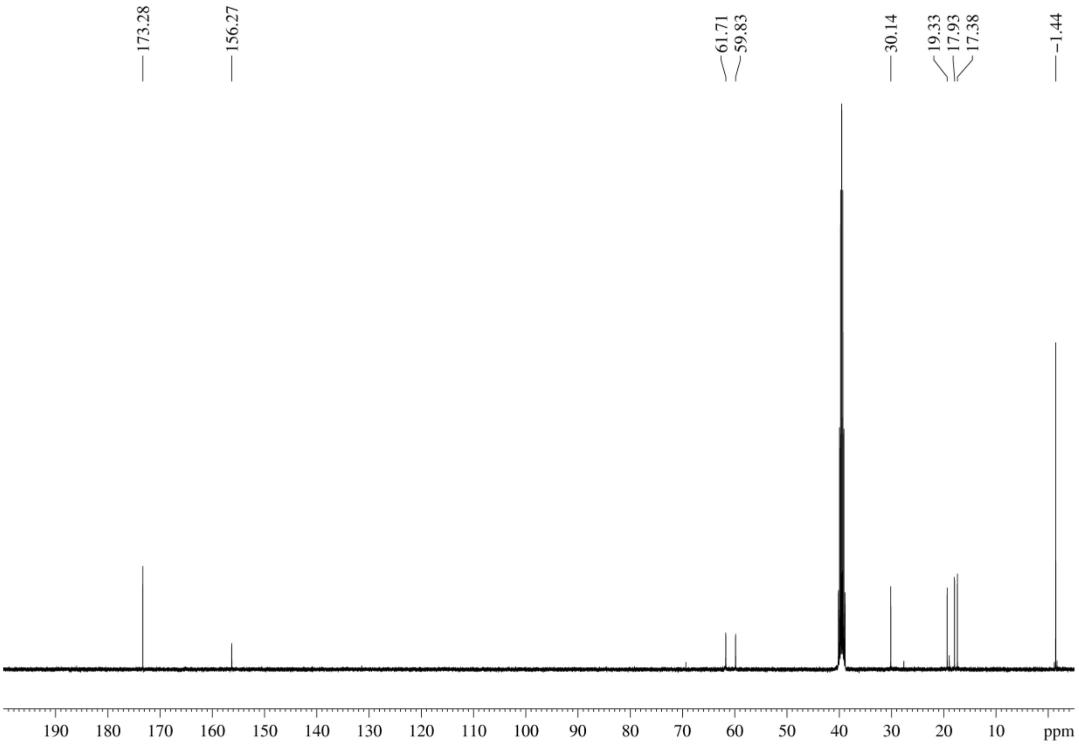
173.28
156.27
61.71
59.83
30.14
19.33
17.93
17.38
-1.44
190
180
170
160
150
140
130
120
110
100
90
80
70
60
50
40
30
20
10
ppm

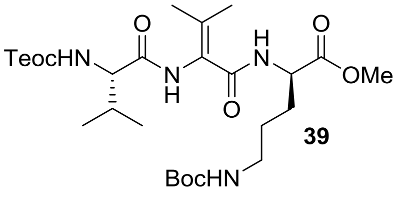
TeocHN
O
N
H
H
N
O
O
OMe
39
BocHN

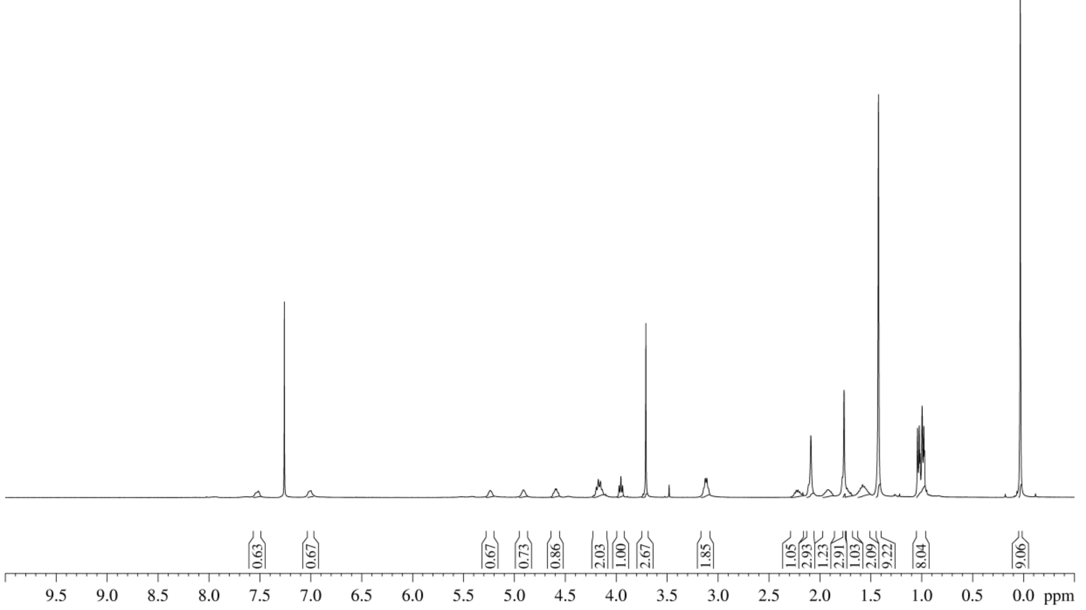
0.63
0.67
0.67
0.73
0.86
2.03
1.00
2.67
1.85
1.05
2.93
1.23
2.91
1.03
2.09
9.22
8.04
9.06
9.5 9.0 8.5 8.0 7.5 7.0 6.5 6.0 5.5 5.0 4.5 4.0 3.5 3.0 2.5 2.0 1.5 1.0 0.5 0.0 ppm

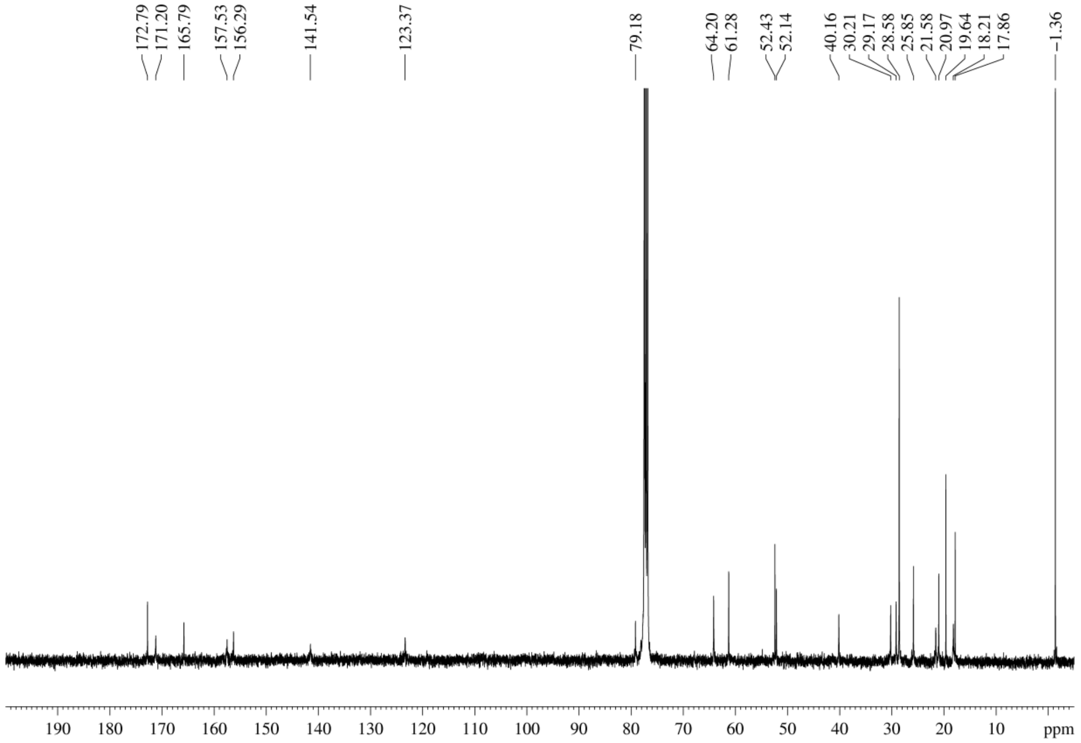
172.79
171.20
165.79
157.53
156.29
141.54
123.37
79.18
64.20
61.28
52.43
52.14
40.16
30.21
29.17
28.58
25.85
21.58
20.97
19.64
18.21
17.86
-1.36
190 180 170 160 150 140 130 120 110 100 90 80 70 60 50 40 30 20 10 ppm

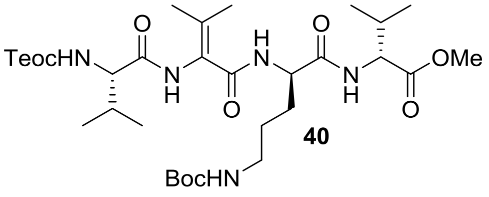

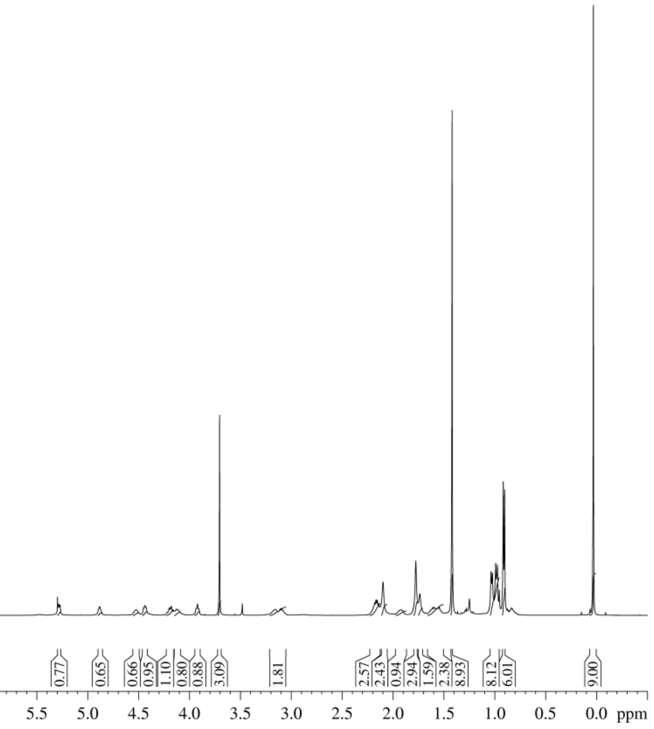

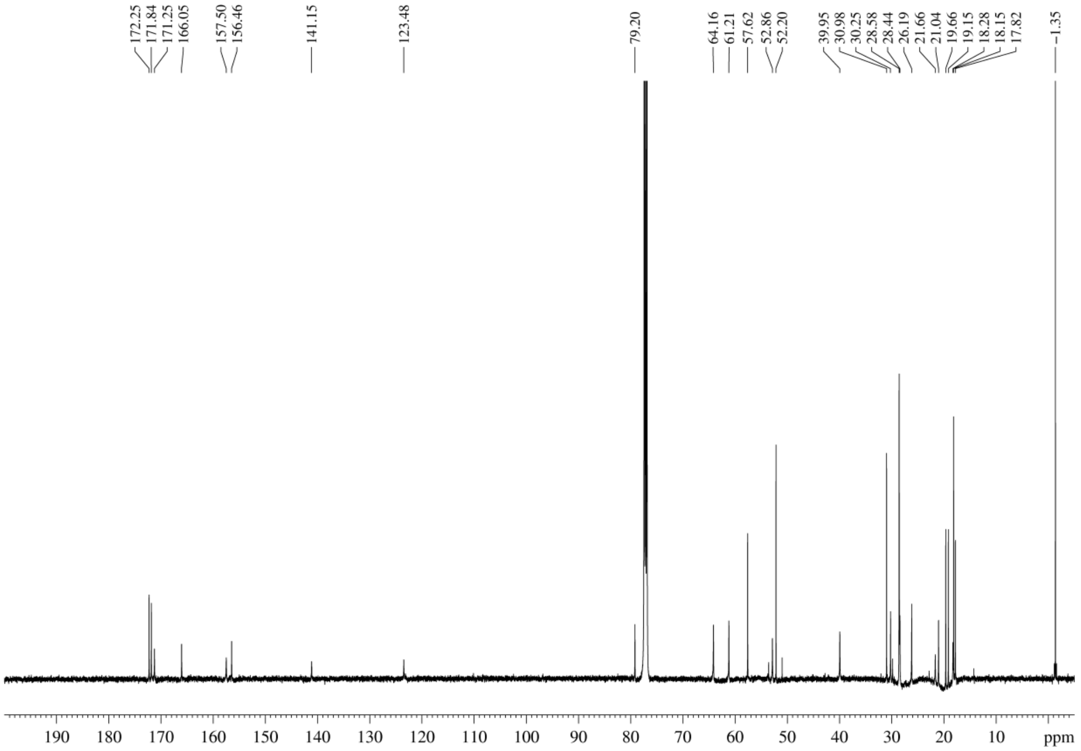

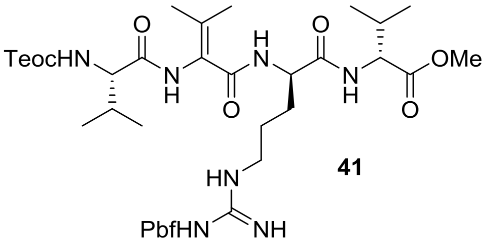
TeocHN
OMe
PbfHN
NH
HN

41

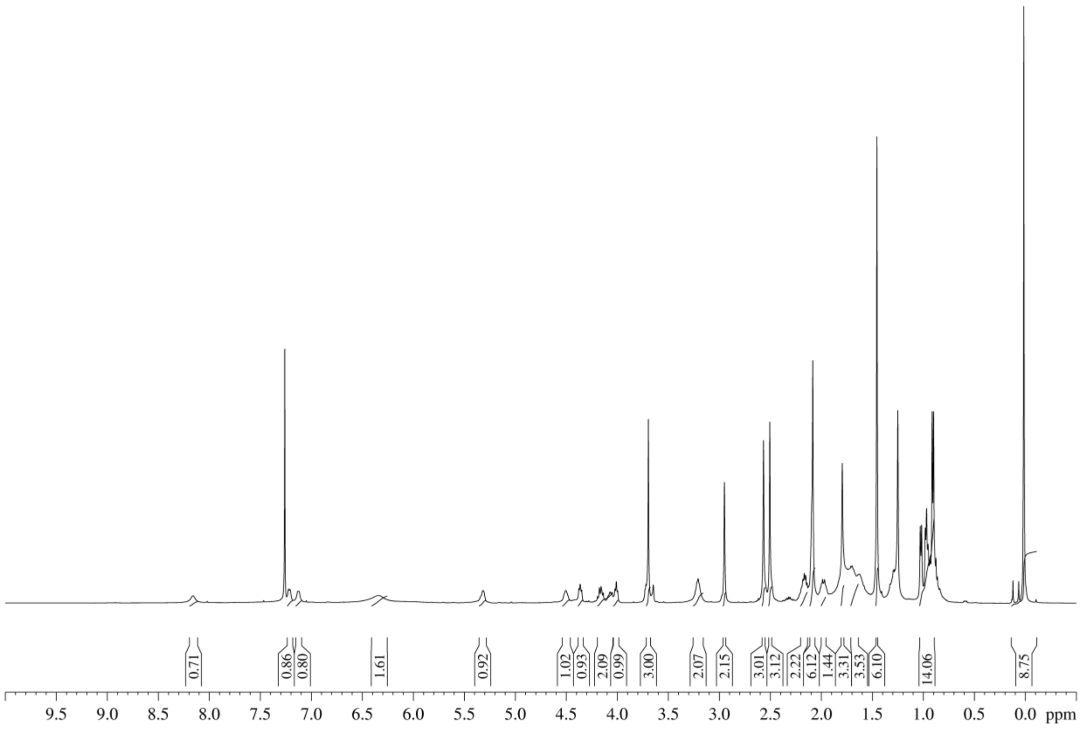
0.71
0.86
0.80
1.61
0.92
1.02
0.93
2.09
0.99
3.00
2.07
2.15
3.01
3.12
2.22
6.12
1.44
3.31
3.53
6.10
14.06
8.75
9.5 9.0 8.5 8.0 7.5 7.0 6.5 6.0 5.5 5.0 4.5 4.0 3.5 3.0 2.5 2.0 1.5 1.0 0.5 0.0 ppm

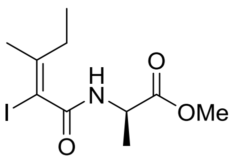

O
H
N
OMe
I
O

42

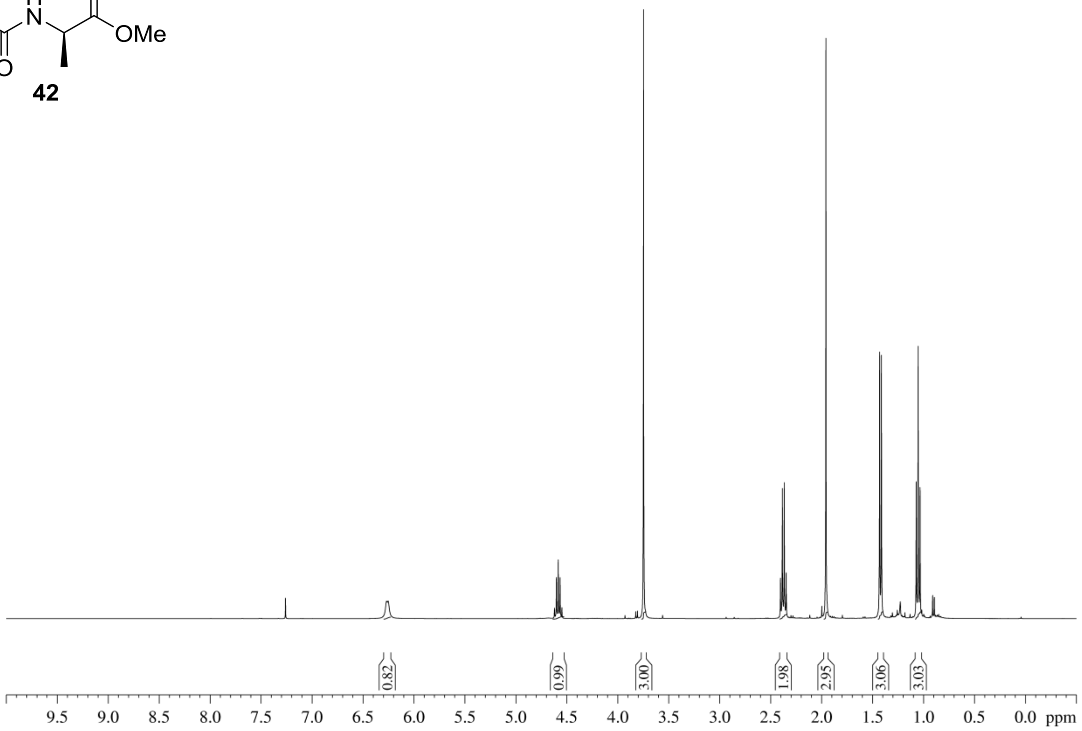

0.82
0.99
3.00
1.98
2.95
3.06
3.03
9.5 9.0 8.5 8.0 7.5 7.0 6.5 6.0 5.5 5.0 4.5 4.0 3.5 3.0 2.5 2.0 1.5 1.0 0.5 0.0 ppm

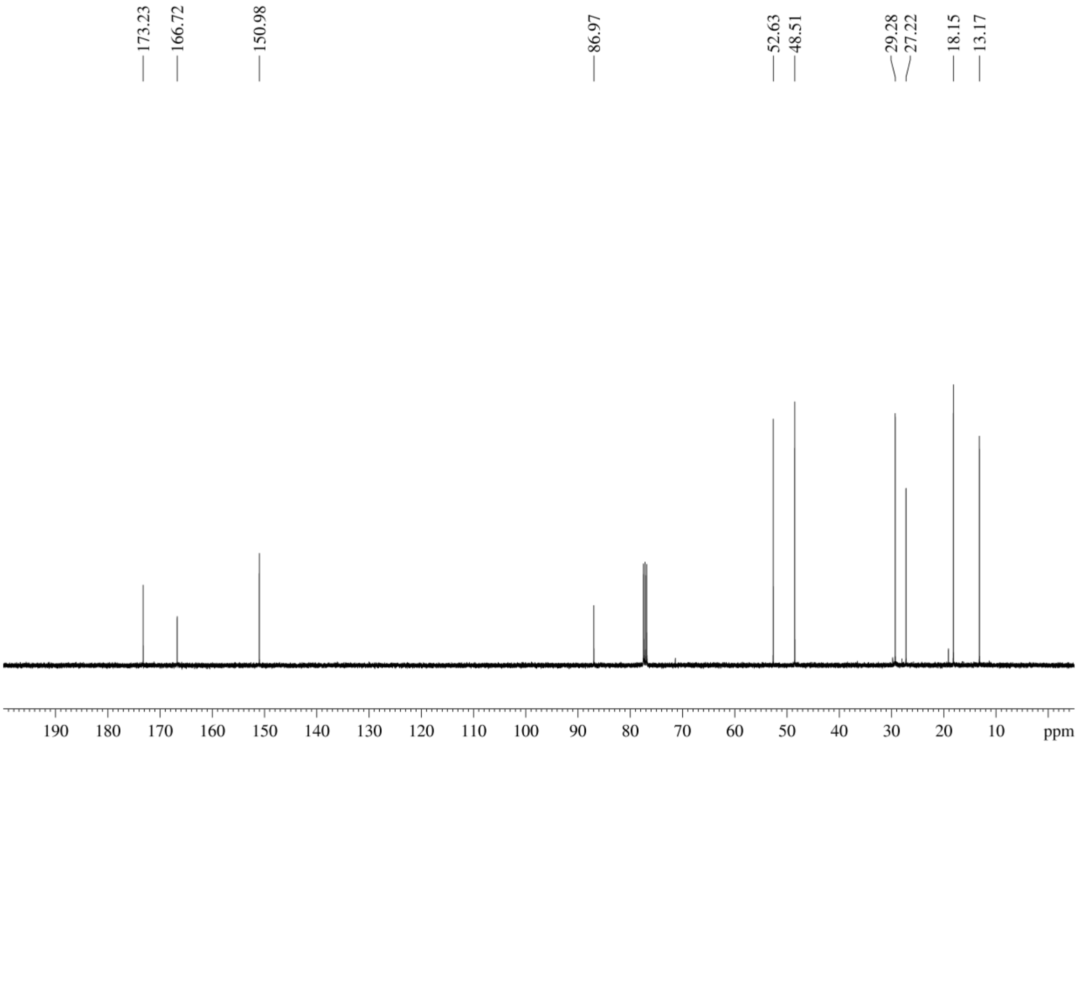

173.23
166.72
150.98
86.97
52.63
48.51
29.28
27.22
18.15
13.17
190 180 170 160 150 140 130 120 110 100 90 80 70 60 50 40 30 20 10 ppm

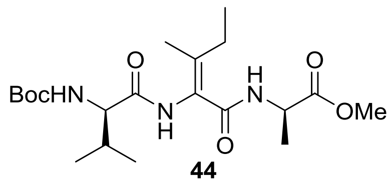
BocHN
O
H
N
N
H
O
O
OMe

44

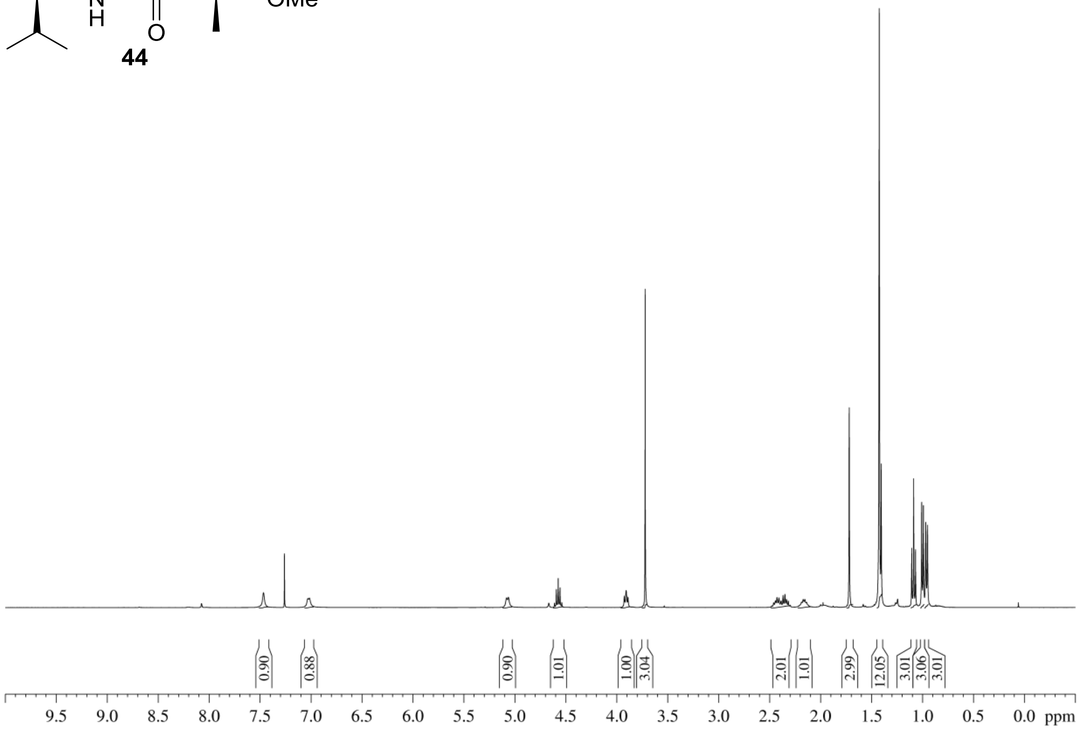
0.90
0.88
0.90
1.01
1.00
3.04
2.01
1.01
2.99
12.05
3.01
3.06
3.01
9.5 9.0 8.5 8.0 7.5 7.0 6.5 6.0 5.5 5.0 4.5 4.0 3.5 3.0 2.5 2.0 1.5 1.0 0.5 0.0 ppm

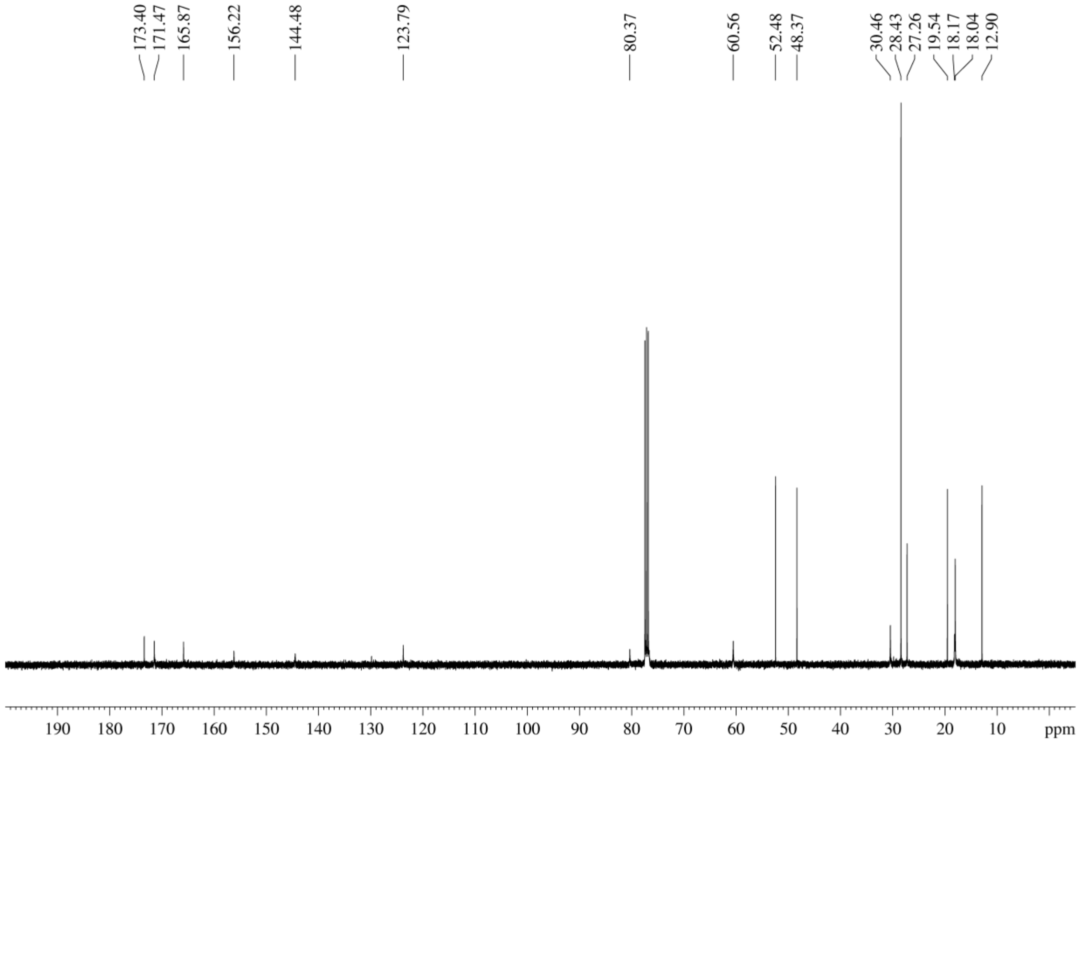
173.40
171.47
165.87
156.22
144.48
123.79
80.37
60.56
52.48
48.37
30.46
28.43
27.26
19.54
18.17
18.04
12.90
190 180 170 160 150 140 130 120 110 100 90 80 70 60 50 40 30 20 10 ppm
